# Physical Activity, Exerkines, and Their Role in Cancer Cachexia

**DOI:** 10.3390/ijms26168011

**Published:** 2025-08-19

**Authors:** Jan Bilski, Aleksandra Szlachcic, Agata Ptak-Belowska, Tomasz Brzozowski

**Affiliations:** 1 Department of Biomechanics and Kinesiology, Institute of Physiotherapy, Faculty of Health Sciences, Jagiellonian University Medical College, 31-008 Cracow, Poland; 2 Department of Physiology, Faculty of Medicine, Jagiellonian University Medical College, 31-531 Cracow, Poland; aleksandra.szlachcic@uj.edu.pl (A.S.); agata.ptak-belowska@uj.edu.pl (A.P.-B.)

**Keywords:** cancer cachexia, gastrointestinal cancers, physical activity, exerkines, skeletal muscle, systemic inflammation, metabolic dysfunction, context-dependent signaling

## Abstract

Cancer-associated cachexia is a multifaceted wasting syndrome characterized by progressive loss of skeletal muscle mass, systemic inflammation, and metabolic dysfunction and is particularly prevalent in gastrointestinal cancers. Physical activity has emerged as a promising non-pharmacological intervention capable of attenuating key drivers of cachexia. Exercise modulates inflammatory signaling (e.g., IL-6/STAT3 and TNF-α/NF-κB), enhances anabolic pathways (e.g., IGF-1/Akt/mTOR), and preserves lean body mass and functional capacity. Exercise-induced signaling molecules, known as exerkines, are key mediators of these benefits, which are released during physical activity and act in an autocrine, paracrine, and endocrine manner. However, many of these molecules also exhibit context-dependent effects. While they exert protective, anti-inflammatory, or anabolic actions when transiently elevated after exercise, the same molecules may contribute to cachexia pathogenesis when chronically secreted by tumors or in systemic disease states. The biological effects of a given factor depend on its origin, timing, concentration, and physiological milieu. This review presents recent evidence from clinical and experimental studies to elucidate how physical activity and exerkines may be harnessed to mitigate cancer cachexia, with particular emphasis on gastrointestinal malignancies and their unique metabolic challenges.

## 1. Introduction

Cancer-associated cachexia (CAC) is a complex wasting syndrome characterized by involuntary loss of body mass and skeletal muscle tissue. This condition may or may not involve the loss of adipose tissue and is accompanied by systemic inflammation and metabolic disturbances that cannot be fully reversed with standard nutritional support [1]. This syndrome is caused by reduced nutrient intake (anorexia) and metabolic alterations caused by tumor-induced factors [1,2]. The incidence of cachexia depends on the type of cancer and may affect approximately 70% of cancer patients and account for more than 20% of deaths among cancer patients [3]. It is particularly common in patients with gastrointestinal (GI) tract cancers, particularly those of the esophagus, stomach, and pancreas. For example, cachexia affects as many as 87% of pancreatic and gastric cancer patients, contributing to poor tolerance of therapy and roughly 20–30% of cancer-related deaths [4,5].

GI tract cancers represent a significant global health challenge, constituting approximately 26% of global cancer cases and 35% of cancer-associated mortalities. This group of cancers, including esophageal, gastric, and pancreatic cancers, often exhibits a challenging clinical course complicated by metabolic disturbances [6,7]. Importantly, CAC is one of the most devastating metabolic complications of GI cancers [1]. This syndrome results from both reduced nutritional intake (anorexia) and metabolic changes caused by tumor-induced factors [1,2].

Physical activity has been proposed as a potential intervention for cancer patients [8,9,10]. Although patients with advanced GI cancers often suffer from fatigue and functional decline, emerging evidence suggests that exercise is feasible and may offer benefits to this population [8,9,10,11,12,13,14,15,16,17]. The molecular mechanisms underlying the beneficial effects of exercise in cancer remain unclear. However, there is evidence indicating that physical activity and exercise-related substances (called “exerkines”) may have an essential role in regulating the systemic inflammatory milieu involved with cachexia [17,18].

In CAC, exerkines and related molecules may have dual roles: some drive the pathology of cachexia, whereas others, exercise-induced factors, may counteract cachectic wasting. Recent studies have elucidated the molecular mechanisms by which these factors influence the pathogenesis of cachexia, identified their potential as biomarkers of cachexia progression, and explored them as therapeutic targets or adjuncts to treatment [1,9,14].

In this review we focus on cancer cachexia linked to upper GI cancers, specifically esophageal, gastric, and pancreatic tumors as these malignancies are characterized by a high prevalence of cachexia and distinct metabolic and inflammatory profiles. We considered both preclinical and clinical studies, focusing on publications from the past decade, giving special consideration to randomized controlled trials (RCTs), meta-analyses, and mechanistic studies that provide insights into the molecular pathways involved in cachexia and its modulation through PA.

This review aimed to examine how physical activity and exercise-induced signaling molecules, known as exerkines, affect the pathogenesis and progression of cachexia in these cancers. We discuss the dual roles of these molecules, which can be either protective or harmful, depending on their release context, and highlight their therapeutic potential. We aimed to address critical knowledge gaps, including the limited understanding of exerkine-specific mechanisms in cancer cachexia, variability in patient responses to exercise interventions, and methodological inconsistencies in current research. We propose future directions to improve exercise-based treatments and utilise exerkines for their therapeutic benefits in this high-risk patient population by combining knowledge from multiple fields.

## 2. Cancer Cachexia: Mechanisms and Clinical Implications

### 2.1. Defining Cancer Cachexia

The term “cachexia” comes from the Greek “kakos” and “hexis,” meaning “bad physical condition.” Cancer cachexia is a major syndrome of multifactorial host-phagocytic-dependent waste, which is associated with a progressive loss of skeletal muscle mass (with or without loss of fat mass) [5,19]. This condition is often accompanied by anorexia, systemic inflammation, metabolic disturbances, and impaired myogenesis (Table 1). Cancer cachexia differs from simple malnutrition or sarcopenia in that it involves a more profound system-wide catabolic state driven by both tumor-derived factors and host responses that include metabolic, immunological, and neurological alterations [5,19].

According to the international consensus reached in 2011 [19], cancer cachexia is diagnosed by fulfilling established criteria that include unintentional weight loss exceeding 5% over the previous 6 months or weight loss of more than 2% in patients with a BMI of less than 20 kg/m^2^.

Cachexia develops through three phases: a pre-cachectic phase with early metabolic changes, anorexia, and glucose intolerance (weight loss ≤ 5%); a cachectic phase with persistent weight loss > 5% together with systemic inflammation and reduced food intake; and a refractory cachectic stage characterized by an increased degree of catabolism, with poor performance status (ECOG < 3) and a life expectancy of less than 3 months [19].

Based on the increasing prevalence of overweight and obesity, especially in elderly cancer patients, the European Society of Clinical Nutrition (ESPEN) recommended a higher cut-off value of 22 kg/m^2^ for defining cancer cachexia in this population. This change takes into account the masking effect of weight loss in obese patients, which has led to the underdiagnosis of cancer cachexia in this expanding population [5].

Cachexia is particularly common in patients with GI cancers such as pancreatic, gastric, and esophageal cancers [20,21]. The prevalence of cachexia is very high in patients with advanced cancer, particularly pancreatic and gastric cancers, with rates as high as 85% and 87%, respectively [20,22]. A recent study by Tao et al. [23] confirmed that cachexia is found in 75% of patients with gastric cancer. Cachexia also affects a considerable proportion of the patients diagnosed with EC. Estimates suggest that cachexia may be present in approximately 50–80% of patients at diagnosis, particularly among those with advanced stages of the disease [20,21,24,25]. This multifactorial condition is particularly pronounced because EC often affects nutritional intake, leading to a cycle of weight loss and increased cachexia severity [25,26].

### 2.2. Pathophysiology of Cachexia: Molecular Mechanisms

**Figure 1:**
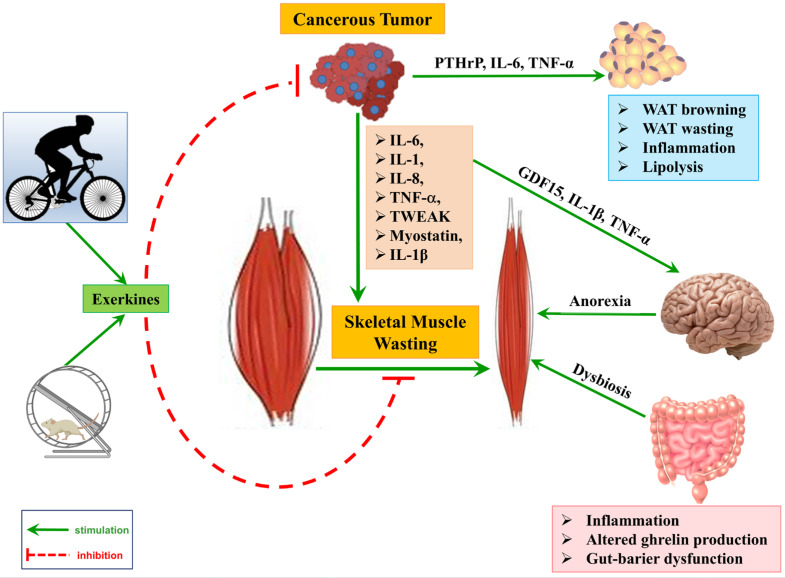
Mechanisms of cancer-induced skeletal muscle wasting and modulatory role of exercise. PTHrP: Parathyroid Hormone-related Protein; IL-1: Interleukin-1; IL-6: Interleukin-6; IL-8: Interleukin-8; IL-1β: Interleukin-1 beta; TNF-α: Tumor Necrosis Factor-alpha; GDF15: Growth Differentiation Factor 15; WAT: White Adipose Tissue.

The pathophysiology of cachexia is complex and involves multiple interactions between the tumor and the host’s metabolic, immune, and neurological systems [1]. These interactions ultimately lead to an abnormal metabolic state, which is defined by muscle wasting, anorexia, and systemic inflammation (Figure 1). These processes are mediated by cytokines and tumor-derived factors, and inflammatory markers are largely responsible for muscle degradation. In addition, changes in the hypothalamic control of food intake and energy expenditure worsen this state [1,24].

#### 2.2.1. Pro-Inflammatory Cytokine Network

The pro-inflammatory cytokine network plays an important role in the tumor-induced systemic catabolic state, which is responsible for muscle wasting, anorexia, and metabolic disorders in cancer cachexia [1]. These pro-inflammatory cytokines, such as IL-6 and TNF-α, are released by both tumor and host immune cells and initiate a cascade of intracellular signaling pathways that culminate in skeletal muscle degradation and other systemic dysregulation [1].

The IL-6/STAT3 signaling pathway is critical for cancer cachexia-induced muscle loss. Elevated levels of this pro-inflammatory cytokine, IL-6, are observed in many cancers, as well as in preclinical models, and IL-6 is a major pro-inflammatory factor involved in the systemic inflammatory condition that occurs in cachexia [27,28]. IL-6 activates the Janus kinase (JAK)-STAT pathway by binding to its glycoprotein 130 (gp130) receptor to specifically activate Signal Transducer and Activator of Transcription 3 (STAT3). STAT3 subsequently translocates to the nucleus and initiates the transcription of a variety of genes associated with muscle atrophy, collectively referred to as atrogenes [27].

Atrogenes such as muscle-specific RING finger protein 1 (MuRF1) and atrogin-1 are key regulators of muscle protein degradation. These E3 ubiquitin ligases help mark muscle proteins for degradation via the ubiquitin-proteasome pathway (UPS). This leads to the breakdown of proteins in skeletal muscle fibers, contributing to the muscle wasting and atrophy seen in cachexia [27]. Furthermore, IL-6-induced STAT3 activation appears to augment the proteolytic process and interfere with muscle cell differentiation and repair, thereby hindering muscle regeneration and leading to chronic muscle loss [27].

Another active pathway involved in the pathogenesis of cancer cachexia is the TNF-α/NF-κB pathway. TNF-α, also known as cachectin, is a pro-inflammatory cytokine that plays a crucial role in cachexia, and its production is linked to muscle loss and systemic inflammation [1,3]. TNF-α, via TNFR1 and TNFR2 receptors, triggers the NF-κB signaling pathway [1,3] and promotes protein degradation via ubiquitin proteasome E3 ligases, specifically MurF1 and Atrogin1 [29], which are common in cancer patients and preclinical models of cachexia, where they facilitate the degradation of skeletal muscle proteins [30,31]. Additionally, TNF-α contributes to weight loss by stimulating lipolysis, inhibiting lipogenesis [29], and inducing anorexia [32]. TNF-α elevates corticotropin-releasing hormone (CRH) levels, which subsequently diminishes appetite and food intake [29].

Activin A and myostatin, both members of the TGF-β superfamily, and TNF-like weak inducer of apoptosis (TWEAK), a cytokine belonging to the TNF superfamily, have all been implicated in muscle wasting [33,34]. They interact with either type I or type II activin receptors in the skeletal muscle, resulting in the activation of Smad signaling pathways [33,34,35,36,37]. Some tumors actively produce and secrete Activin A leading to elevated circulating levels in cancer patients with cachexia, which correlates with muscle wasting and weight loss [38]. Activin A promotes muscle wasting by inducing endothelial dysfunction in skeletal muscle vasculature and activating catabolic pathways that lead to muscle atrophy [38,39].

TWEAK performs its biological function by binding to the TWEAK receptor (TweakR) or fibroblast growth factor-inducible 14 (Fn14) with physiological affinity [40]. The TWEAK-Fn14 system functions as a vital signaling pathway that controls skeletal muscle mass in different physiological and pathological states [41].

The TWEAK–Fn14 signaling pathway leads to skeletal muscle wasting in cancer cachexia via various molecular pathways [42,43]. TWEAK activates NF-κB in myocytes through pathways that are similar to TNFα and IL-1, which results in the transcription In cancer cachexia, TWEAK–Fn14 signaling contributes to skeletal muscle wasting through multiple molecular mechanisms. A key downstream effect is the activation of NF-κB in myocytes: TWEAK, similar to TNFα and IL-1, induces the transcription of muscle-specific E3 ubiquitin ligases such as MuRF1 (TRIM63) and Atrogin-1 (MAFbx) via NF-κB and p38/CCAAT–enhancer-binding protein β pathways. E3 ligases mark structural muscle proteins for destruction, which accelerates proteasomal proteolysis and leads to muscle atrophy [42,43].

At the same time, pro-inflammatory signaling (NF-κB, cytokines) suppresses anabolic pathways by inhibiting the insulin/AKT/mTOR axis, further tipping the balance toward protein breakdown and reducing protein synthesis [42]. Recent studies have demonstrated that TWEAK–Fn14 signaling can also trigger endoplasmic reticulum (ER) stress in muscles [43]. In a pancreatic cancer cachexia model, Fn14 activation in muscle fibers led to the upregulation of the protein kinase R-like endoplasmic reticulum kinase (PERK) and inositol-requiring protein (1α IRE1α) arms of the unfolded protein response, which correlated with suppressed protein synthesis [43]. The pharmacological inhibition of PERK TWEAK performs its biological function by binding to the TWEAK receptor (TweakR) or fibroblast growth factor-inducible 14 (Fn14) with physiological affinity [40]. The TWEAK-Fn14 system functions as a vital signaling pathway that controls skeletal muscle mass in different physiological and pathological states [41].

Pharmacological PERK inhibition leads to enhanced protein synthesis and increased myotube diameters in muscle cells treated with TWEAK, thus indicating that ER stress plays a role in TWEAK-mediated muscle degradation [43]. TWEAK’s effects on muscle are not limited to proteolysis; they also promote local and systemic inflammation. TWEAK binding to Fn14 on muscle cells has been shown to cause an NF-κB–dependent increase in Pax7 [42], thereby blunting muscle repair and regeneration. Cachexia involves broader metabolic disturbances beyond muscles, and TWEAK–Fn14 signalling likely contributes to adipose tissue wasting and systemic metabolic alterations as well [44].

Pancreatic ductal adenocarcinoma (PDAC) has one of the highest incidences of cachexia among cancers; up to ~80% of PDAC patients develop cachexia, often severe [45]. Emerging studies in recent years have highlighted the pivotal role of the TWEAK–Fn14 axis in pancreatic cancer-induced cachexia. A breakthrough study [46] revealed a novel tumor–immune cell crosstalk that initiates cachexia in pancreatic cancer. In PDAC mouse models, tumor-associated macrophages have been shown to “license” tumor cells to secrete TWEAK. Mechanistically, pancreatic tumor cells recruit CCR2^+^ macrophages via CCL2; which in turn secrete CCL5, which activates the TRAF6/NF-κB pathway in the tumor cells, inducing high secretion of [46]. The tumor-derived TWEAK circulates to the muscle, where it binds Fn14 on muscle fibers and triggers atrophy by upregulating MuRF1 and initiating muscle protein breakdown. Depleting macrophages or disrupting the CCL2–CCR2 and CCL5–CCR5 signaling loops between macrophages and tumor cells significantly attenuated muscle wasting in these model [46]. This study revealed a feed-forward loop in which pancreatic tumors “hijack” immune cells that drive TWEAK production, thereby causing paracrine muscle destruction. The study also found that TWEAK expression was elevated in human PDAC patients with cachexia and that, higher TWEAK levels were associated with weight loss and cachectic status, underscoring TWEAK’s relevance as a cachexia mediator in pancreatic cancer [46].

Cachexia is also associated with excessive levels of IL-1β, which cause muscle wasting and anorexia in affected patients. IL-1β acts on the hypothalamus, where it promotes neuropeptide Y (NPY) levels but suppresses the expression of known appetite-promoting factors such as agouti-related peptide (AgRP), thereby decreasing food intake. This reduced appetite can lead to the energy deficit characteristic of cachexia [47,48]. In addition to its effects on appetite, IL-1β enhances muscle degradation by stimulating signaling pathways in muscle tissues (i.e., such as the NF-κB and mitogen-activated protein kinase (MAPK) pathways). This activation results in increased protein degradation via the UPS and autophagy-lysosomal pathways. Additionally, IL-1β inhibits the IGF-1/Akt pathway, an anabolic pathway that normally stimulates muscle growth and repair. IL-1β promotes muscle loss and blocks muscle regeneration by blocking this pathway [47]. Remarkably, pancreatic cancer patients experiencing weight loss showed significantly increased serum levels of IL-6 and TNF-α compared to those whose weight remained stable [49].

Interferon-gamma (IFN-γ), a pro-inflammatory cytokine mainly secreted by T cells and natural killer (NK) cells, plays a pivotal role in cancer cachexia by triggering the JAK-STAT signaling pathway in muscle cells. When IFN-γ binds to its receptor, it activates JAK1 and JAK2 kinases, which then phosphorylate STAT1, a member of the STAT family [27,48]. STAT1 subsequently enters the nucleus, where it promotes the activation of genes related to inflammation and immune control. The IFN-γ pathway also increases the ubiquitylation-dependent out-of-control (runaway) degradation of IFN-γ in muscle cells via the ubiquitin-proteasome system. IFN-γ also markedly represses muscle protein synthesis by suppressing mechanistic target of rapamycin (mTOR) signaling, which is critical for muscle hypertrophy. This two-fold effect contributes to muscle loss in cachexia by facilitating catabolism and suppressing anabolism. In addition, IFN-γ-induced inflammation destroys muscle mitochondria and leads to energy depletion, resulting in muscle atrophy [27,48].

#### 2.2.2. Key Proteolytic Systems in Muscle Wasting

Proteolysis of skeletal muscle proteins is the principal hallmark of cancer cachexia and is mediated by several proteolytic systems that synergistically degrade myofibrillar and other cellular proteins that contribute to muscle wasting. The activation of these proteolytic systems (the ubiquitin-proteasome system [UPS], the autophagy-lysosomal pathway, and the calpain system) is highly regulated and changes dramatically in cachexia [47].

In cancer cachexia, the ubiquitin-proteasome system (UPS) is the main pathway for muscle protein degradation [47,48]. This system is particularly important for the targeted degradation of myofibrillar proteins, which are structural proteins of the muscle fibers. The UPS consists of a group of enzymes, such as E1-ubiquitin-activated enzyme, E2-ubiquitin-conjugating enzyme, and E3-ubiquitin ligases, which mediate the binding of proteins to be broken down [47]. In cachexia, there is upregulation of specific E3 ubiquitin ligases, such as muscle-specific RING-finger protein 1 (MuRF1) and muscle atrophy F-box protein, also known as atrogin-1 (MAFbX), which are strongly linked to muscle wasting. These ligases catalyze the attachment of ubiquitin molecules to proteins, marking them for recognition and degradation by the 26S proteasome [1,47].

In addition to these, other ubiquitin ligases have emerged as key players in muscle catabolism during cachexia, including the tumor necrosis factor (TNF) receptor-associated factor 6 (TRAF6) [50] and the muscle ubiquitin ligase of SCF complex in atrophy-1 (MUSA1) [51].

TRAF6 is a RING-family E3 ubiquitin ligase and adaptor protein which enables signaling pathways to transmit downstream signals from TNF receptor superfamily and Toll-like receptors [52,53,54]. It has been suggested that TRAF6 functions as a key factor in skeletal muscle atrophy, including cancer cachexia [53]. Data from both mouse models of cancer cachexia and muscle specimens from human cancer patients have shown that TRAF6 expression and activity are enhanced in atrophying skeletal muscle. A study on patients with gastric cancer suffering from cachexia found a significant upregulation of TRAF6 in the skeletal muscle, which was associated with increased ubiquitin expression and weight loss [55].

MUSA1, along with other muscle-specific E3 ligases like MuRF1 and atrogin-1, is involved in the protein degradation processes that drive muscle atrophy. The activation of E3 ligases like MUSA1 has been linked to various signaling pathways, including the NF-κB pathway. Inhibition of this pathway has been shown to reduce the expression of multiple E3 ligases, including MUSA1, suggesting that MUSA1 expression is downstream of key inflammatory signals that are central to cancer cachexia.

MUSA1, is a muscle-specific F-box protein that functions as part of an SCF ubiquitin ligase complex [54]. MUSA1 was identified relatively recently as an atrogene upregulated in conditions of muscle disuse. In mouse models of denervation or immobilization [56], Musa1 expression is strongly induced, which correlates with increased proteolysis and muscle fiber atrophy [54]. Importantly, MUSA1 appears to mediate a specific atrophy program distinct from the canonical MuRF1/Atrogin-1 pathway [54].

Although MUSA1 was initially characterized in disuse atrophy, accumulating evidence indicates that it is also involved in cancer cachexia across different tumor types. Transcriptomic analyses of muscle from cachectic cancer patients have revealed elevated levels of MUSA1 in comparison to non-cachectic controls [57].

In cancer cachexia, components of the UPS are activated by the inflammatory cytokines TNF-α and IL-6 via the NF-κB and STAT3 pathways, which upregulate MuRF1 and atrogin-1 expression. Such pathways promote a hypercatabolic state in the muscle, causing protein degradation and contributing to the systemic symptoms of cachexia [1,47].

Autophagy is a lysosomal process that involves degradation of damaged organelles, misfolded proteins, and other cell debris. It is important for cellular homeostasis, particularly in the skeletal muscle, which requires cellular turnover and constant remodeling of proteins for muscle maintenance. Cachexia is associated with inappropriately upregulated autophagy, resulting in the excessive degradation of muscle proteins and organelles [1,47]. Several important pathways, most notably the mechanistic target of rapamycin (mTOR) pathway, also play a key role in regulating autophagy. In the catabolic state of cachexia, mTOR activity is reduced and autophagy is increased. Moreover, increased autophagy has been linked to increased oxidative stress, leading to greater muscle damage. The combined effects of autophagy and UPS-mediated degradation facilitate sustained loss of muscle mass in cachexia [1,47].

The calpain system, consisting of calcium-dependent proteases, plays a significant role in the initiation of muscle proteolysis. These proteases, particularly calpain-1 and calpain-2, which are activated by high intracellular calcium concentrations, promote the dissociation of myofibrillar proteins in skeletal muscle, allowing their subsequent degradation by the UPS system. This phenomenon is observed in cachexia, in which intracellular calcium levels are elevated because of mitochondrial dysfunction and oxidative stress in the muscle. Calpain activation also causes muscle membrane disruption, leakage of intracellular contents, and muscle cell damage. This proteolytic activity is an important factor in the early stages of muscle degradation, setting the stage for extensive degradation by the UPS and autophagy [1,47]. Reduced muscle protein synthesis plays a significant role in muscle loss in cancer cachexia, thereby exacerbating the effects of protein degradation. In healthy muscle tissue, protein synthesis is mediated by the anabolic signal-activated mTOR pathway [1,47].

#### 2.2.3. Mitochondrial Dysfunction

Mitochondrial dysfunction plays an important role in the development and progression of cancer cachexia by impairing muscle energy metabolism, increasing oxidative stress, and disrupting calcium homeostasis [58,59]. The reduced oxidative capacity, increased oxidative stress, impaired mitophagy, and energy inefficiency exacerbate the loss of muscle mass and lead to functional impairment [27,48]. Inflammatory signaling has been implicated in cancer-induced mitochondrial dysfunction in skeletal muscle [59]. NF-κB, STAT3, and Smad3 signaling have been associated with cancer-induced muscle mitochondrial dysfunction in tumor-bearing mice [59].

Mitochondrial dysfunction is associated with the overproduction of reactive oxygen species (ROS) [60]. The involvement of ROS in muscle atrophy observed in cancer cachexia has been suggested because ROS production is increased in cachexia-affected skeletal muscle and the involvement of ROS in skeletal muscle atrophy has been well documented [60].

#### 2.2.4. Anabolic Resistance

Anabolic resistance, defined as a reduced capacity of skeletal muscle to respond to anabolic stimuli, is a hallmark of cancer cachexia. Wasted muscles fail to respond to normal anabolic signals, even with nutritional or hormonal interventions to prevent muscle loss. The key molecular processes responsible for this condition include reduced response to amino acids, impaired sensing of mechanical load, and dysregulation of the GH/IGF-1 system, which further exacerbates progressive muscle atrophy [3,61].

#### 2.2.5. Neuroendocrine Dysregulation and Anorexia

Anorexia, characterized by a loss of appetite and/or reduced food intake, is one of the most devastating symptoms of cancer cachexia. Loss of appetite in cancer cachexia is not solely due to psychological distress or adverse effects of cancer treatment; the progressive decrease in food intake is a consequence of biological mechanisms, mainly neuroendocrine dysfunction, which disrupts the physiological regulatory control of hunger and satiety. Anorexia significantly affects quality of life, leading to malnutrition, loss of skeletal muscle, and attenuated efficacy of anti-cancer therapies [3,62].

Recent research on cancer cachexia has underscored the involvement of the neuroendocrine system, with particular emphasis on the roles of the hypothalamus, pituitary gland, and adrenal gland in appetite regulation [3,62]. The hypothalamus is essential for maintaining energy homeostasis, primarily through its signaling pathways that modulate food intake and, conversely, decrease energy expenditure [63,64].

The arcuate nucleus (ARC) of the brain is pivotal in regulating energy balance. It contains two distinct neuronal populations: those reliant on agouti-related protein (AgRP)/neuropeptide Y (NPY), and those reliant on proopiomelanocortin (POMC)/cocaine- and amphetamine-regulated transcript (CART). Activation of AgRP/NPY neurons by hormones leads to an increase in appetite, whereas activation of POMC/CART neurons suppresses appetite and reduces metabolic activity. Under hunger conditions, AgRP and NPY promoted food intake and decreased energy expenditure. Conversely, the POMC derivative melanocyte-stimulating hormone (MSH) and CART peptide inhibit food consumption and enhance energy expenditure when there is an energy surplus. Insulin and leptin, which are secreted in accordance with the body’s energy reserves, inhibit AgRP/NPY neuronal activity while stimulating POMC/CART neurons. Additionally, gastric ghrelin promotes food intake by activating AgRP/NPY neurons and inhibiting POMC/CART neurons [64].

Neuroendocrine dysregulation is crucial in the pathogenesis of cancer cachexia. The neuroendocrine system, controlled by the hypothalamic–pituitary–adrenal (HPA) axis and the sympathetic nervous system (SNS), regulates appetite, energy balance, and stress responses [65]. In cancer cachexia, these pathways are disrupted by tumor-derived factors and systemic inflammation, which further worsen cachexia and subsequent clinical outcomes [3,66]. Anorexia in cancer cachexia is closely associated with persistent inflammation and pro-inflammatory cytokine production in the hypothalamus, leading to deactivation of NPY/AgRP neurons and activation of POMC/CART neurons [3,62,63,65].

The hypothalamic–pituitary–adrenal (HPA) axis demonstrates hyperactivity in cancer cachexia owing to ongoing stress and inflammation [67]. Tumors and cytokines activate the hypothalamic–pituitary–adrenal (HPA) axis and maintain elevated cortisol levels. Cytokines increase the production of corticotropin-releasing factor, which along with prostaglandins, inhibits the synthesis of neuropeptide Y (NPY) [67]. Elevated cortisol levels, resulting from increased corticotropin-releasing hormone (CRH) and adrenocorticotropic hormone (ACTH) levels, promote catabolism of muscle proteins and lipolysis. Excess cortisol reduces insulin sensitivity and accelerates tissue degradation via the ubiquitin-proteasome pathway and the suppression of IGF-1 [65,67].

#### 2.2.6. Ghrelin Biology, Resistance, and Therapeutic Modulation

Ghrelin is a 28-amino acid peptide primarily synthesized and secreted by the X/A-like enteroendocrine cells in gastric mucosa [68]. This peptide has strong orexigenic effects that lead to increased food intake. In circulation, ghrelin exists in two forms: acylated ghrelin (AG) and unacylated ghrelin (des-acyl ghrelin, UnAG). The acylated form is the biologically active isoform, known to bind to its primary receptor, growth hormone secretagogue receptor-1a (GHS-R1a), thereby mediating the canonical physiological actions of ghrelin. In contrast, UnAG, although traditionally considered inactive at GHS-R1a, accounts for approximately 90% of total circulating ghrelin and has been implicated in biological activities through as-yet-unidentified receptors [68,69]. Physiologically, ghrelin levels peak before meals (pre-prandial) and rapidly decrease following food intake, reflecting its key role in the acute regulation of appetite. Ghrelin concentrations are observed to increase during periods of fasting or in states of negative energy balance, such as starvation or anorexia, and conversely, they are suppressed in conditions of energy surplus, like obesity [68].

Paradoxically, circulating ghrelin levels tend to be elevated in cachectic patients, likely as a compensatory response to weight loss and anorexia [3]. However, cachexia is associated with a “ghrelin resistance,” in which high ghrelin levels fail to trigger a normal increase in appetite or weight gain; therefore, patients remain anorexic despite the presence of the hormone [3]. In a rat model of cancer, tumor-bearing rats had elevated ghrelin levels but did not eat more or gain weight, confirming ghrelin resistance in cachectic states [70]. This resistance blunts the protective effects of ghrelin and contributes to ongoing muscle wasting and malnutrition in cachectic patients [3].

Ghrelin also has protective effects on skeletal muscle. It acts as an endogenous ligand for GHS-R1a and is a potent stimulator of GH secretion, activating signalling pathways that counteract muscle wasting [3]. Ghrelin also acts via GH-independent pathways to suppress the pro-inflammatory and catabolic milieu that drives cachexia as it stimulates the release of the anti-inflammatory cytokine IL-10 while reducing levels of TNF-α, IL-1β, and IL-6. This shift toward an anti-inflammatory profile helps reduce muscle breakdown caused by these cytokines [3,71].

At the molecular level, ghrelin inhibits key muscle-wasting pathways and downregulates the NF-κB-mediated ubiquitin-proteasome system in muscles. It thereby blocks the expression of muscle-specific ubiquitin ligases, Atrogin-1 and MuRF1, which drive protein degradation [3,71]. In the mouse model, ghrelin administration strongly attenuated dexamethasone-induced muscle atrophy by inhibiting Atrogin-1/MuRF1 through PI3Kβ, mTORC2, and p38 MAPK signalling [72]. Similarly, in muscle cell cultures, ghrelin blocks protein degradation induced by catabolic cytokines such as TNF-α [70].

Exercise can influence ghrelin levels and tissue sensitivity, offering a potential strategy to combat cachexia. Regular PA improves muscle mass and stimulates appetite in chronically ill patients [73].

Acute bouts of exercise generally lead to transient suppression of acylated ghrelin production [74]. However, following intense exercise bouts or very long-term training programs, there is often a compensatory increase in hunger and potentially higher ghrelin levels. Chronic exercise training, especially when it leads to significant weight loss, can also result in an overall increase in the total and UnAG production [74].

Interestingly, exercise appears to enhance ghrelin sensitivity in skeletal muscles, whereas physical inactivity can worsen ghrelin resistance [75,76]. Experimental studies have shown that prolonged physical inactivity, together with a poor diet, impairs ghrelin’s actions on muscle metabolism [76]. Rats fed a high-fat diet (HFD) while remaining sedentary for six weeks developed muscle ghrelin resistance; ghrelin could no longer stimulate fatty acid oxidation or improve insulin uptake in their muscles [76]. In contrast, rats fed a HFD that underwent exercise training retained normal muscle responses to ghrelin [76]. Exercise training preserves the positive effects of ghrelin on skeletal muscles, preventing metabolic resistance that develops with inactivity. Exercising rats had higher muscle levels of specific receptors (such as the CRF2 receptor) that may mediate ghrelin’s actions, suggesting that exercise can upregulate components of the ghrelin signaling pathway in the muscle [76].

A synergistic interaction between exercise and ghrelin has been suggested [75,77]. Exercise and ghrelin agonists may complement each other’s anabolic effects. Ghrelin activation triggers the GH/IGF-1 axis, increasing circulating IGF-1 and other anabolic signals, while resistance exercise induces local muscle IGF-1 production and boosts protein synthesis [75,77]. Thus, combining a ghrelin stimulant with an exercise regimen could theoretically maximize muscle anabolism via both the systemic and local pathways. It has been hypothesized that such a combination would yield greater improvements in lean mass and function than either intervention alone, especially in conditions of muscle wasting such as cancer [77].

The recognition of ghrelin’s function in appetite and muscle protection has resulted in the creation of ghrelin-mimicking drugs for cachexia treatment. Ghrelin agonists function to bypass endogenous ghrelin resistance by using potent long-acting compounds that activate the ghrelin receptor (GHS-R1a). Multiple clinical trials from the previous decade have evaluated ghrelin and its analogs for the treatment of cancer cachexia patients with positive outcomes. The administration of intravenous ghrelin to advanced cancer patients led to immediate and significant improvements in their appetite and food consumption compared to the placebo group. The patients experienced better meal satisfaction and consumed more calories following ghrelin infusion [78]. The clinical trial involving chemotherapy patients demonstrated that ghrelin treatment both enhanced appetite and decreased nausea symptoms, which helped fight chemotherapy-related anorexia [79]. Early clinical trials conducted between 2004 and 2012 showed that ghrelin could temporarily reverse anorexia in cancer patients [80]. Research now focuses on developing ghrelin receptor agonists, which provide better options for long-term administration. Anamorelin is a leading oral ghrelin-mimetic drug [81]. Ghrelin receptor activation by anamorelin produces metabolic and appetite effects that are identical to those of ghrelin. Clinical trials using anamorelin have demonstrated substantial advantages in patients with cachexia. The treatment resulted in increased lean body mass together with total body weight and appetite scores in patients who had cancer cachexia [81,82,83,84,85]. These positive results have led to regulatory approvals; anamorelin was approved in 2021 in Japan for the treatment of cancer cachexia in non-small cell lung cancer (NSCLC) and gastric, colorectal, and pancreatic cancers [81]. This made it one of the first drugs specifically indicated for the treatment of cancer cachexia. The discovery of liver-expressed antimicrobial peptide-2 (LEAP2) as a natural GHS-R antagonist stands out as an important advancement [86,87]. Thus, the discovery of this novel therapeutic approach has become possible. Ghrelin resistance in cachexia can be treated by blocking LEAP2 action to enhance ghrelin signaling through LEAP2/ghrelin ratio modulation. This new approach focuses on optimizing the receptor agonist and antagonist balance instead of raising ghrelin levels to restore its beneficial effects [86,87,88].

Thus, the therapeutic implications of ghrelin modulation are important. By pharmacologically activating the ghrelin pathway, we can address two hallmark problems of cachexia: anorexia and muscle wasting [80]. Ghrelin agonists stimulate appetite, leading to greater dietary intake, and simultaneously exert muscle-sparing effects via hormone release and direct signaling [89]. This dual action tackles the energy imbalance and the hyper-catabolic state of cachexia. However, although weight gain (especially lean mass gain) with ghrelin agonists is well documented, improvements in physical function and survival are still being studied. Some trials showed improved muscle strength or quality of life, whereas others did not show a significant functional benefit, despite weight gain [89]. Cachexia is a complex syndrome, and ghrelin-based therapy alone may not completely reverse it; however, it forms a critical component of a multimodal approach. The optimal strategy likely combines ghrelin modulation with exercise and nutritional support, along with management of tumor and inflammation [89].

#### 2.2.7. Neuromuscular Junction (NMJ) Instability

In cancer cachexia, there is evidence that this nerve–muscle connectivity is compromised. Preclinical studies have shown that tumor-bearing mice develop structural and functional NMJ abnormalities, leading to partial denervation of muscle fibers. For example, Sartori et al. [90] demonstrated in mouse models that cancer causes marked disruption of NMJ morphology, accompanied by motor nerve withdrawal from muscle fibers. This was mechanistically linked to tumor factors that trigger molecular changes that impair NMJ. Consistently, cachectic mice show muscle fibers with centralized myonuclei and abnormal positioning of nuclei, which is a hallmark of denervation (seen after nerve injury or in aged muscle) [91]. These central nuclei in the cachectic muscle occur without true muscle regeneration and colocalize with denervation markers such as neural cell adhesion molecule (NCAM) and upregulated acetylcholine receptor subunits [91]. The same was observed in muscle samples from patients who have gastrointestinal cancers, where a progressive increase in the number of central myonuclei was observed in weight stable and in cachectic patients, compared to healthy subjects Importantly, denervation-related atrophy seems to preferentially affect fast-twitch fibers: the same fiber type vulnerability observed in age-related sarcopenia, suggesting a shared pathophysiological pattern.

In contrast, Boehm et al. [92] analyzed NMJs in rectus abdominis biopsies from cancer patients (with and without cachexia) and reported that the NMJ structure appeared grossly intact despite significant muscle fiber atrophy. The authors concluded that overt NMJ dismantling was not evident in this context, suggesting that intrinsic muscular changes might underlie atrophy independent of denervation [92].

An important discovery in cachexia research is the role of bone morphogenetic protein (BMP) signaling in preserving muscle mass and innervation. BMP signaling (through Smad1/5/8 activation) is normally a positive regulator of muscle maintenance, promoting protein synthesis and protecting NMJ integrity [3]. This pathway is blunted in cancer cachexia. Tumor-derived factors, particularly Activin A (a TGF-β family cytokine) and IL-6, induce the expression of Noggin, a soluble BMP inhibitor, in the muscle tissue [90]. Elevated Noggin blocks BMPs from activating their receptors on muscle fibers and motor neurons, leading to a cascade of NMJ destabilization, denervation, and muscle wasting [90]. Sartori et al. [90] reported that diminished BMP-Smad1/5/8 signaling is an early event in cachexia, observed in both mice and cancer patients. BMP suppression results in the loss of normal “trophic” signals, which helps stabilize synapses and suppress atrophic genes. Interestingly, restoring BMP signaling can reverse these effects: increasing BMP activity in tumor-bearing mice (via gene therapy or pharmacological means) prevents muscle loss and preserves NMJ structure and function [90].

Proinflammatory cytokines can also play a role in this process. For instance, sustained IL-6 not only wastes muscle via Stat3, but might also hinder the regrowth of nerve terminals (chronic IL-6 can cause neuropathy in some contexts) and impair the differentiation of muscle stem cells. Thus, inflammatory cytokines are both independent causes of cachectic atrophy and facilitators of denervation-associated muscle loss [3].

#### 2.2.8. Gut Barrier Dysfunction, Dysbiosis, and Systemic Inflammation

Both animal models and human studies have suggested that increased intestinal permeability and microbial translocation are linked to the development and severity of cancer cachexia [93]. Gut barrier dysfunction can lead to leakage of bacteria and endotoxins, fueling inflammation and metabolic disturbances [94]. Several mechanisms may lead to gut barrier dysfunction in cancer cachexia. Chronic inflammation in cachexia directly impairs the intestinal barrier. Elevated pro-inflammatory cytokines in cachectic patients disrupt tight junctions between epithelial cells and increase permeability via both apoptotic and nonapoptotic mechanisms [95]. IL-6 signaling upregulates the tight-junction protein claudin-2, leading to a “leaky” gut barrier [96]. In a mouse model of cachexia, IL-6 was found to be necessary for barrier disruption. Apc(Min/+) mice lacking IL-6 do not develop gut leakiness or cachexia, and IL-6 overexpression increases intestinal permeability [97]. Neutralizing IL-6 in cachectic mice preserves tight junction integrity and prevents weight loss [96], underscoring the central role of cytokine-driven mucosal injury.

Dysbiosis in cancer cachexia contributes to intestinal barrier damage [98]. Dysbiotic microbiota in cachexia can erode the mucus layer and induce local inflammation, further weakening the epithelial barrier [98]. Interestingly, the administration of certain *Lactobacillus* probiotics attenuated muscle atrophy in mice, highlighting the gut-muscle axis [96].

Studies using animal models and humans have suggested that increased intestinal permeability and microbial translocation could be associated with the development and severity of cancer cachexia [93]. However, in cancer cachexia, several mechanisms can lead to gut barrier dysfunction. Chronic inflammation, which is present in cancer cachexia, can directly impair the intestinal barrier. The pro-inflammatory cytokines elevated in cancer cachexia disrupt epithelial cell tight junctions, resulting in increased permeability through apoptotic and non-apoptotic pathways [95]. The tight-junction protein claudin-2 shows increased expression through IL-6 signalling, which results in compromised gut barrier function [96]. Dysbiotic microbiota in cachexia can erode the mucus layer and lead to local inflammation, which further weakens the epithelial barrier [98]. Administration of *Lactobacillus* probiotics to mice resulted in a reduction in muscle wasting [96].

Tumors arising within the GI tract can directly disrupt the intestinal mucosa, leading to increased intestinal permeability, endotoxemia, and systemic inflammation. Ulceration or invasion of the gut wall by a tumor can compromise tight junctions and epithelial integrity, creating focal points for bacterial translocation [3].

The tumor presence in the GI tract can also trigger localized inflammation (involving tumor-infiltrating immune cells and cytokine release), which directly impairs the barrier. Severe barrier injury in GI cancers can disrupt normal motility and absorption. Clinically, patients with gastric or intestinal tumors often have anorexia and early satiety, as well as malabsorption and diarrhea if the mucosal barrier is compromised. High intestinal permeability can cause chronic diarrhea, nutrient malabsorption, and energy loss, compounding the cachectic state of GI cancers [3].

### 2.3. The Interrelationship Between Muscle and Bone in Cancer Cachexia

#### 2.3.1. Bone Metabolism in Cachexia

Cancer patients frequently experience bone loss, decreased bone mineral density, and increased fracture risk, leading to osteoporosis [99,100,101]. This phenomenon is traditionally attributed to metastases or therapy side-effects. However, increasing evidence suggests that cachexia itself can disrupt bone homeostasis even in the absence of metastases [102]. Patients undergoing cachexia show increased activity of bone resorption processes. Mechanisms such as chronic systemic inflammation, endocrine disorders (e.g., hypogonadism and insulin resistance), nutritional deficits, and a decrease in the mechanical load on the skeleton resulting from loss of muscle mass and reduced physical activity are responsible for this condition [100,101].

Zwickl and colleagues [103] detected higher levels of carboxy terminal telopeptide of collagen (CTX) in the serum of cancer cachexia patients compared to cancer patients without cachexia. The CTX-to-osteocalcin ratio was significantly higher in patients with cachexia, indicating that bone degradation processes exceed bone formation processes. Approximately 68% of patients with cachexia had a positive bone resorption balance compared to 20% in the control group. In addition, elevated C-reactive protein and reduced albumin levels, which reflect increased inflammation and malnutrition, respectively, were independently correlated with an unfavorable bone turnover profile [103]. These findings suggest that systemic inflammation is a common pathophysiological link between muscle wasting and bone degradation in patients with cachexia. Observed high C-reactive protein and low albumin levels (reflecting inflammation and poor nutritional status) independently correlated with this unfavorable bone turnover, implicating systemic inflammation as a link between muscle and bone wasting [103].

The observed correlations are consistent with the results of preclinical studies. In mouse models of tumor cachexia, animals with tumors exhibit reduced bone mineral density and abnormal bone microarchitecture that co-occur with muscle atrophy [104].

Tumor- and host-derived cytokines (e.g., IL-6 family, TNF-α) increase receptor activator of nuclear factor-κ B ligand (RANKL) expression and inhibit osteoprotegerin (OPG), which drives remodeling toward osteoclastogenesis and cortical/trabecular loss [105]. Preclinical studies have demonstrated that circulating RANKL is sufficient to induce concomitant bone resorption and muscle wasting, and that RANKL blockade attenuates both bone loss and cachexia in tumor-bearing mice [106]. IL-6 signaling exerts complex, context-dependent effects: IL-6 can promote osteoclast formation indirectly via osteoblast lineage cells and, in some cases, directly on progenitors, whereas “classical” versus “trans-“ IL-6 signaling differentially affects bone repair and resorption [107].

Osteocytes are crucial in cancer-induced bone pathology. In many models of nonmetastatic cancer cachexia (C26 colon adenocarcinoma, ES-2 ovary, LLC lung), severe osteocyte apoptosis and osteolysis were observed, with increased lacunar surface area and increased osteoclast gene expression in the osteocytes themselves; co-culture studies implicate tumor-secreted factors as drivers of these changes [106,108]. Osteocyte-derived Wnt signaling inhibitors (sclerostin, DKK1) and RANKL further link osteocyte stress to impaired formation and increased resorption, mechanisms that may be modulated by exercise [106,108,109].

In general, cancer cachexia induces an osteopenic phenotype through increased osteoclast activity and impaired osteoblast function. Underlying this mechanism are likely the same pro-inflammatory cytokines and catabolic signals that are responsible for muscle tissue atrophy, confirming the existence of a common pathophysiological axis for muscle and bone damage [101,110].

The pathophysiology of cancer cachexia involves profound degeneration of the musculoskeletal system, where skeletal muscle is the direct target of pathological processes. At the same time, bone loss, although often underestimated, is a constant and important component of this syndrome. The occurrence of concomitant changes in muscle and bone tissue indicates a coordinated musculoskeletal pathogenesis [101,110].

#### 2.3.2. Muscle–Bone Crosstalk in the Cancer Cachexia

Skeletal muscle and bone form an integrated musculoskeletal system through their anatomical and functional connection which enables essential health maintenance through their dynamic tissue relationship [101,111]. The interaction between both tissues occurs via physical forces and biochemical signals [101,111]. Bone tissue receives mechanical stimuli through the action of physical forces generated by gravity, locomotor activity, and physical exercise, while osteokines and myokines secreted by bones and muscles, respectively, act as mediators of biochemical communication between these tissues [101,111]. The bidirectional nature of this interaction means that any disturbances affecting muscle mass or function can affect bone tissue homeostasis not only by reducing mechanical loading but also by modulating molecular signaling pathways [101]. Maintaining the structural integrity of bone tissue is largely dependent on the functional state of the skeletal muscle, as pathological muscle atrophy leads to a simultaneous reduction in mechanical stimuli and beneficial biochemical signaling. Mechanical unloading resulting from the loss of muscle mass results in reduced osteoblast mechanostimulation, leading to reduced anabolic activity and increased osteocyte apoptosis, thereby disrupting bone remodeling processes. Muscle wasting leads to a reduction in mechanical load on the skeleton, which accelerates bone loss in a manner analogous to that observed during physical inactivity. Simultaneously, chronic inflammation that accompanies muscle wasting blunts anabolic signaling (e.g., decreased levels of IGF-1) and increases catabolic signaling (e.g., increased expression of myostatin and activin), resulting in inhibition of osteoblastogenesis and increased bone resorption [112].

Catabolic factors released from cachectic muscles can have adverse effects on bone metabolism. Myostatin, produced mainly by skeletal muscles, not only acts as a negative regulator of their growth, but also directly interferes with osteogenesis [113]. Myostatin inhibits osteoblast differentiation via osteocyte-derived signals, including those mediated by exosomal microRNA-218 [114]. Consistent with these observations, myostatin-deficient mice showed increased bone mineral density and strength, an effect that is partially abolished by unloading, indicating a synergistic effect of muscle mass and muscle-bone signaling [115].

In cancer cachexia, elevated levels of myostatin and activin A correlate with reduced markers of bone formation. Another muscle-derived factor released during catabolism is β-aminoisobutyric acid (BAIBA), which stimulates osteocyte activity. Its levels can change under cachexia, further affecting bone balance [113].

Chronic inflammation resulting from wasting muscle and adipose tissue cachexia leads to an increase in circulating pro-inflammatory cytokines, such as IL-6 and TNF-α, which stimulate osteoclastogenesis and enhance bone resorption [103]. In addition, the loss of mechanical load associated with muscle atrophy results in increased sclerostin expression by osteocytes, leading to the inhibition of osteoblast activity and reduced bone formation [113,116].

As a result, cachectic muscle atrophy and accompanying reduced physical activity generate both attenuated anabolic signals and increased catabolic signals, which together contribute to bone degradation [113].

On the other hand, bone-derived factors can also modulate mass and skeletal muscle function. One of the most prominent examples in the context of cachexia is RANKL ligand, known primarily as a key regulator of bone resorption through osteoclast activation [106,117]. Increasing evidence suggests that RANKL may also affect muscle metabolism, highlighting the importance of bidirectional communication between bone and muscle tissue in the pathophysiology of cachexia [106]. In a mouse model of ovarian cancer cachexia, high circulating RANKL was sufficient to cause both accelerated bone loss and severe muscle atrophy; treatment with osteoclast-targeted therapies (denosumab or bisphosphonate zoledronic acid, which inhibit RANKL signaling) not only preserved bone mass but also significantly attenuated muscle wasting and weakness [106]. This indicates RANKL acts as a bone-derived osteokine that can exacerbate muscle degeneration in cachexia, and that blocking bone resorption has spill-over benefits on muscle [106,117]. Bone metastases themselves, when present, further skew bone–muscle crosstalk by releasing factors like TGF-β from bone matrix; TGF-β can enter circulation and induce ubiquitin ligases in muscle, hastening atrophy [113,118]. On the other hand, osteocalcin, released by osteoblasts especially during exercise, improves muscle glucose uptake and endurance capacity [119]. It is necessary for optimal muscle function in mice, and older animals with boosted osteocalcin show enhanced exercise performance [119]. This suggests that bone loss in cachexia (with reduced osteocalcin output) could remove a beneficial signal for muscle maintenance.

Muscle–bone crosstalk abnormalities are increasingly recognized as key contributors to cancer cachexia’s onset and progression [113]. This recognition has important implications: it suggests that an effective intervention should ideally target both muscle and bone, breaking the cycle of combined musculoskeletal decline. Exercise is one such intervention that inherently engages the muscle–bone unit and modulates many of the signaling pathways described above [120].

### 2.4. Adipose Tissue Dysfunction in Cancer Cachexia

Decreased adipocyte surface area and perimeter alterations are the main morphological changes observed in both animal models and in cachectic patients. Studies in cachectic mice have shown that adipocyte remodeling goes beyond simple size reduction and involves complex morphological rearrangements [121,122]. The adipose tissue of these mice showed shrunken adipocytes of various sizes containing several smaller lipid droplets surrounded by altered mitochondria, irregular cell outlines, and dilated interstitial spaces enriched with capillaries [121,122]. Fibrosis formation and inflammatory cell infiltration contribute to architectural modifications in cachectic adipose tissue. Importantly, morphological alterations depend on the type of adipose tissue (visceral or subcutaneous). In cachectic patients with gastrointestinal cancer, significant visceral adipose tissue depletion is observed compared with that in healthy individuals [121,122].

Extracellular matrix (ECM) remodeling is an integral part of adipogenesis and the establishment of tissue architecture [122,123]. Cancer cachexia in both murine models and human patients is characterized by increased fibrosis, which contributes to the reorganization of adipose tissue. In cancer cachexia, ECM remodeling in adipose tissue is characterized by changes in collagen deposition, increased numbers of infiltrating cells, and development of insulin resistance [122,123,124].

Adipose tissue in cachectic patients exhibits fibrosis due to the enhanced synthesis and deposition of collagen fibers [123]. Recent studies have demonstrated that type I collagen content in the subcutaneous adipose tissue of patients with gastrointestinal cancer cachexia is rearranged, leading to increased infiltration of macrophages and lymphocytes [122,124]. In particular, cachectic patients with gastrointestinal cancer display architectural modifications in the subcutaneous adipose tissue due to fibrosis and infiltration of inflammatory cells surrounding adipocytes in areas of fibrosis [122,124].

In cancer cachexia, lipolytic enzymes are upregulated, leading to accelerated triglyceride catabolism in the adipose tissue. Increased activity of hormone-sensitive lipase (HSL) and adipose triglyceride lipase (ATGL) enhances triglyceride hydrolysis and enables the release of glycerol and free fatty acids (FFAs) into the blood [121]. Furthermore, factors secreted by the tumor, such as zinc-alpha-2-glycoprotein (ZAG), further promote triglyceride hydrolysis via ATGL, aggravating fat depletion. Increased lipolytic activity worsens the negative energy balance and causes systemic metabolic derangements that contribute to cachexia by shifting energy demand and promoting skeletal muscle loss [121].

Increased circulating FFA levels correlate with the worsening muscle protein breakdown observed in cancer cachexia [125]. Therefore, adipose tissue atrophy due to increased lipid mobilization represents a key point in the onset and progression of cancer cachexia, which significantly contributes to the negative energy balance and promotes the skeletal muscle wasting observed in cachectic patients.

In cancer cachexia, major phenotypic changes are observed in adipose tissue, including the so-called “browning” of white adipose tissue (WAT). During this process, a fraction of white adipocytes transform into thermogenic “beige” or “brite” adipocytes characterized by a high number of mitochondria and increased expression of uncoupling protein 1 (UCP1) [121,126,127].

UCP1 uncouples mitochondrial respiration from ATP synthesis, allowing energy to be released as heat via thermogenesis and resulting in faster weight and fat loss [126,127]. Tumor-derived and systemic factors, most notably IL-6, tumor-secreted PTHrP, and ZAG, transactivate thermogenic gene expression in different adipose tissue depots, resulting in the browning process [128,129]. WAT browning-induced increased thermogenesis aggravates the metabolic inefficiency associated with cancer cachexia [121,126,127]. Studies in mice have shown that a phenotypic switch from white adipose tissue to brown-like (beige) fat occurs in the early stages of cachexia, even before significant muscle wasting occurs [130]. Browning of WAT is associated with upregulation of UCP1 and mitochondrial uncoupling, leading to increased energy expenditure and lipid burning in cachectic animals [130]. A landmark study revealed that parathyroid hormone-related protein (PTHrP) secreted by tumors can cause extensive browning of adipose tissue in mice with cancer cachexia [128].

Neutralizing PTHrP in a cachexia tumor model blocked the browning of white adipose tissue while preventing muscle loss, suggesting thermogenic activation of fat as a causative factor in cachexia for muscle wasting [128]. Inflammatory cytokines such as IL-6 have also been shown to induce beige fat development in cachexia models [130]. By increasing browning signals, tumors efficiently convert fat depots into energy-burning tissues. This energy drain accelerates weight loss: one study estimated that browning and uncoupled thermogenesis were responsible for a significant portion of the increased resting energy expenditure in cachexia mice [130].

However, a study by Rohm et al. [131] has shown in several experimental models that although previous studies have detected mild induction of UCP1 mRNA levels in tumor-exposed AT, such changes appear to be discrete in thermogenic terms. This observation suggests that the overall effect of AT UCP1-dependent thermogenesis on systemic energy homeostasis may not be a major factor in cancer cachexia [131].

The clinical significance of thermogenic fat in human cachexia is an active area of research. Although preclinical data suggest that browning of BAT and WAT may be associated with tumor cachexia [129,130,132,133], clinical studies have shown inconsistent results [134,135,136,137]. Historically, it has been hypothesized that cancer patients with metabolically active BAT may be prone to more rapid weight loss due to “wasted” calories such as heat. However, recent clinical data have suggested a more nuanced picture. A large retrospective cohort study (over 14,000 cancer patients) assessed the presence of BAT using PET scans and found no association between the presence of brown fat and the likelihood or severity of cachexia [136]. Importantly, the presence of detectable BAT did not correlate with worse survival in patients with cachexia. In fact, there was a slight trend towards better outcomes in patients with active BAT (although not statistically significant), aligning with the broader observation that BAT in humans often correlates with overall metabolic health [136].

In a recent investigation, Panagiotou et al. [137] conducted a retrospective cohort study to examine the relationship between the presence of brown adipose tissue (BAT) and the risk of cancer cachexia within a one-year period following cancer diagnosis. The authors found that patients with detectable BAT exhibited a significantly lower risk of developing cachexia and experienced less weight loss than those without detectable BAT. They concluded that the presence of BAT may have a protective effect against cancer cachexia and suggested that BAT could be a marker for improved overall metabolic health [137].

These results contradict previous views that BAT activation potentially exacerbates cachexia [133,134,138] and suggest that BAT-derived factors may even be protective in patients. This raises the question: Could BAT-derived factors mitigate some aspects of cachexia rather than exacerbating them? Further investigation of the molecular and physiological roles of AT browning and its thermogenic capacity in cancer cachexia is warranted.

### 2.5. Altered Adipokine Secretion

Adipokines, substances secreted by adipose tissue, influence various physiological processes, including inflammation, metabolism, and appetite regulation, which play complex roles in cancer cachexia. In this pathological state, the initiation of inflammation and dysfunction of adipose tissue contribute to the dysregulation of pro- and anti-inflammatory adipokine synthesis and secretion [122].

When elevated leptin levels are observed in cachectic patients, hyperleptinemia is not consistently observed despite significant weight loss in cancer patients [139]. The role of leptin in the regulation of appetite and energy expenditure has been implicated in metabolic alterations observed during cachexia, particularly in patients with gastrointestinal cancer [140]. Leptin production and sensitivity can be further modulated by increased levels of proinflammatory cytokines, contributing to the cachectic state [141].

Leptin displays varied behavior in cancer cachexia across different cancer types. In a study by Kerem et al. [142], increased leptin levels were found in cachectic patients with gastric cancer and negatively correlated with BMI. In contrast, a study by Diakowska et al. [143] reported lower leptin levels in cachectic patients with esophageal cancer, with leptin and BMI predicting cachexia with a 90% accuracy.

Leptin acts through the JAK/STAT and NF-κB pathways to activate immune cells, leading to increased inflammation [144]. High leptin levels in CAC increase the production of TNF-α, IL-6, and IL-1β, which may worsen muscle loss [145]. Exercise typically lowers leptin levels, and limited available evidence shows that physical activity helps reduce leptin-driven inflammation in cachexia [146].

Adiponectin is produced by adipocytes and circulates in three isoforms: high, middle, and low. This peptide exerts anti-inflammatory and insulin-sensitizing properties and exhibits a paradoxical profile in CAC [122]. Low adiponectin levels are associated with obesity-associated cancers [147], whereas cachectic patients, especially those with gastrointestinal cancers, present with high levels of adiponectin [148,149]. It has been suggested that the observed increase in adiponectin levels is an adaptive response to loss of adipose tissue and muscle [142,150]. Interestingly, recent animal models have shown reduced adiponectin levels [122,151]. In a recent study, Massart et al. [152] investigated AdipoRon (AR), a synthetic adiponectin receptor agonist, in the treatment of cancer cachexia. AR in C26 and Apc(Min/+) mouse models of colorectal cancer reduced body weight loss and muscle wasting while restoring muscle strength. AR has shown anti-inflammatory effects by lowering IL-6 levels, reducing muscle inflammation, decreasing corticosterone production, and improving lipid metabolism by lowering triglyceride levels in the Apc(Min/+) model [152]. These observations suggest that adiponectin may switch from protective to detrimental effects depending on disease progression.

Adiponectin is recognized as an exerkine that has an important role in mediating the metabolic and anti-inflammatory beneficial effects of physical activity [153,154]. Previous research has demonstrated that moderate-to-vigorous aerobic and resistance exercises, when performed over weeks to months, significantly increase circulating adiponectin levels in humans [154,155]. Aerobic exercise consistently increases adiponectin levels, while resistance exercise can also increase adiponectin levels; however, the evidence is less consistent, and the magnitude of change often correlates with factors such as exercise intensity, duration, and changes in body composition [154,156]. The effect of acute exercise sessions on adiponectin levels is variable; some studies have shown no immediate change after a single session, whereas others have reported increases depending on exercise intensity, duration, and individual characteristics [154,157]. Adiponectin exerts its effects through AdipoR1 and AdipoR2 receptors, increasing insulin sensitivity, reducing inflammation, and exerting cardioprotective effects through the activation of AMPK pathways in muscles and other tissues that are similar to those induced by exercise [153]. Increased adiponectin levels induced by physical activity are associated with better glycemic control, reduced inflammation, improved lipid parameters, and lower risk of obesity, all of which cumulatively lead to improved metabolic health [153].

Resistin, an inflammatory and insulin resistance-related adipokine, has also been implicated as a contributing factor in cancer cachexia. Elevated resistin levels have been reported in cachectic patients with gastric and esophageal cancer cachexia, and resistin levels are inversely correlated with BMI [142,148,158]. Exercise generally tends to lower resistin levels, which may be beneficial in the context of cancer cachexia [159].

Moreover, the interaction between adipokines and myokines increases the complexity of cancer cachexia, influences whole-body metabolism, and plays a role in systemic inflammation observed in cancer cachexia [160].

## 3. Physical Activity in Cancer Cachexia

Over the past decade, noteworthy progress has been made in understanding the development of cancer cachexia, leading to improved management and treatment approaches. While pharmacological interventions are available, they are best complemented with supportive nonpharmacological therapies to enhance their efficacy [17,161]. Despite these advancements, strategies for malignant cancers are largely palliative, and some specific anti-cancer therapies might even worsen cancer cachexia [162]. An integrated approach should include pharmacological therapy, nutrition and exercise. Physical activity is vital for cancer treatment and prevention, lowering the risk of recurrence and improving survival rates, and is fundamental in efforts to combat or slow the progression of cancer cachexia [13,17,163]. However, it should be noted that the effects of physical exercise on the physiological and pathophysiological parameters of the body depend on the type, duration, and frequency [164].

### 3.1. Exercise Intervention in Cancer Cachexia

Recent studies on patients with cancer cachexia have shown that exercise interventions are safe and feasible. The positive effects of these interventions on muscle mass, physical function, and quality of life have been reported in several narrative and systematic reviews [8,9,10,11,12,13,14,15,16,17].

Resistance training is particularly effective in counteracting muscle wasting, and mixed exercise modalities may address multiple aspects of cachexia syndrome [14]. A recent randomized trial on pancreatic cancer cachexia demonstrated that a 3-month resistance training program was safe and led to improvements in muscle strength, functional mobility, and even increased lean body mass [165].

The methodological heterogeneity of the studies notwithstanding, there is a consistent trend to include structured physical activity as an integral part of multimodal therapy for cachexia [8,9,12,14,16]. A recent systematic review of 12 controlled trials (898 patients) on exercise-based interventions for cancer cachexia by Cheung et al. [8] showed high adherence (median program completion 75%) and very few adverse events, demonstrating that even patients with advanced cancer can safely participate in exercise programs. Most studies have shown positive effects on specific clinical outcomes, with the most notable being changes in body composition and muscle strength [8].

The effects on functional ability (as measured by endurance and gait tests) and quality of life were more equivocal; however, approximately half of the studies showed improvement. This suggests that, although exercise may improve muscle performance even during cachexia, translating this into better overall function and quality of life may require longer or combined interventions. A review suggested that exercise is best used as part of a multicomponent strategy for the treatment of cachexia, in conjunction with nutrition and medical therapy, to maximize its impact [8].

In line with that, a major phase III trial called MENAC (Multimodal Exercise, Nutrition, Anti-inflammatory Treatment for Cachexia) was recently completed in patients with advanced lung or pancreatic cancer (two cancers with high cachexia incidence). MENAC tested a 6-week intervention that included aerobic and resistance exercises, nutritional support with omega-3 supplementation, and an anti-inflammatory (NSAID), compared to standard care [166]. These results showed that multimodal intervention effectively stabilized body weight relative to that of patients in the standard care group [166]. By week 6, the intervention group had essentially no weight loss (+0.05 kg), whereas the control group had lost ~1 kg [166].

These results are promising, considering that achieving body weight stabilization is a key goal in cachexia. However, it should be noted that the study did not observe a significant difference in muscle mass (measured by computed tomography (CT) between the groups in such a short period [166]. Despite multimodal therapy, both groups still lost some muscle area, indicating that more potent or longer interventions may be needed to regain the muscle [166]. These outcomes suggest that while exercise (with nutrition and NSAIDs) can prevent weight loss in patients with advanced cancer, maintaining or increasing skeletal muscle mass remains a challenge, possibly because of the aggressive biology of the tumor and the short duration of intervention. Nonetheless, MENAC provides evidence that cachexia can be partially mitigated in practice, emphasizing the importance of combining modalities [166].

Cancer cachexia often occurs early in life in patients with pancreatic cancer, leading to the rapid deterioration of their overall physical condition. The P-Move study (2024) evaluated supervised exercise in patients with advanced pancreatic and biliary tract cancer after first-line chemotherapy [167]. These conditions are known to have poor outcomes, but the study showed that exercise intervention was feasible and safe, with no reported adverse events. Participants in the exercise group showed substantial improvement in physical function, as measured by walking and strength tests. In contrast, the condition of those in the control group deteriorated [167].

Fatigue was reduced and quality of life was stable in the exercise group (unlike in the control group). These are remarkable findings, considering that ~80% of the subjects presented with cachexia at study entry and were receiving palliative interventions. Although the trial was small, it suggests that even among frail patients with GI cancer, functional benefits can be achieved with an individually tailored exercise plan [167].

Recent studies suggest that both aerobic and resistance exercise can affect cancer cachexia via distinct molecular mechanisms [8,14,168,169]. Diverse types of exercises provide different physiological responses, and these findings support their use as a treatment for cachexia. Resistance training significantly increases lean body mass, muscle strength, and overall functionality in patients with cancer cachexia [17]. Indeed, there is much consistent evidence to support the notion that resistance training enhances muscle strength in patients with cancer cachexia [8,14]. It is most effective in reducing muscle wasting, as evidenced by 80% of measured parameters from studies showing a positive effect on muscle strength [8,14,168]. Resistance training prevents cytokine-mediated muscle catabolism by modulating inflammatory mediators and anabolic signaling, including IGF-1/Akt/mTOR signaling [120]. Lambert [170] highlighted the molecular advantages of resistance training for muscles, including increased muscle protein synthesis, dampening of pro-inflammatory cytokines (IL-6 and TNF-α), and enhanced net muscle protein balance. These changes in molecular mechanisms underlie several beneficial effects, such as increased muscle strength, maintenance of lean body mass, and overall improvement in quality of life [120].

Conversely, aerobic exercise offers benefits primarily through modulation of metabolic profiles and systemic inflammation. It also improves cardiovascular fitness and functional capacity in patients with cancer cachexia. However, its effects on skeletal muscle mass are less pronounced than those of resistance training [168]. Aerobic training may improve mitochondrial function, reduce oxidative stress, and attenuate pro-inflammatory cytokines (e.g., IL-6 and TNF-α), which are indirectly related to muscle preservation and increase the threshold for cardiovascular fitness and fatigue [10]. Low-to-moderate-intensity aerobic exercise appears to be particularly beneficial for the management of fatigue and maintenance of daily activities [8,168]. An intriguing study by Morinaga et al. [171] showed that aerobic activity promotes beneficial effects via modulation of adiponectin signaling, which is essential for preventing muscle loss upon exposure to cancer cachexia.

However, it has been suggested that exercise training may worsen muscle status in cancer cachexia owing to comorbidities [34,172]. In a mouse model of colon cancer, colon adenocarcinoma-26 (C26), endurance training did not prevent the loss of body weight and muscle mass and even worsened the condition of mice in the early stages of cancer cachexia [172]. In contrast, Khamoui et al. [173] observed that neither aerobic nor resistance training alone prevented tumor-induced weight loss in a similar mouse model of cancer cachexia. Additionally, they observed that resistance training induced the expression of genes related to muscle damage and repair, and some resistance-trained C26 mice required euthanasia before the end of the experiment because of their deteriorating condition [173].

The combined effects of aerobic exercise and resistance training have been reported to be superior to those of individual training modalities [15,174,175,176]. Multimodal training regimens consistently provide substantial benefits across the body size, composition, function, and exerkine production profiles [9,177,178]. Multimodal exercise interventions effectively address the multifactorial pathology of cancer cachexia by targeting anabolic resistance with resistance exercise and systemic metabolic dysfunction with aerobic exercise [9,177,178]. This comprehensive approach enhances muscle mass preservation, improves systemic metabolism, and ultimately boosts physical functionality and the quality of life. Early findings strongly suggest that these combined strategies are more effective than single-modality interventions in preserving physical function and quality of life [9,177,178].

The implementation of preventive exercise programs at the time of cancer diagnosis is effective in preventing cachexia initiation and progression. These early treatment modalities capitalize on patients’ remaining physical abilities, which increases the likelihood of adherence and response. Early exercise preserves muscle mass and function, and has been shown to modify treatment-induced decline. Integrating exercise into cancer treatment concurrently with oncologic interventions has been shown to prolong resistance and counteracts cachexia [8,13,177,179,180,181].

Despite the superiority of preventive measures, therapeutic exercise interventions after cachexia onset have been beneficial. Although less effective than prevention, these strategies have the potential to improve patient outcomes by ameliorating muscle wasting, increasing functional capacity, and slowing disease progression, even in patients with advanced disease. Evidence suggests that muscles remain responsive to exercise training, with the ability to improve protein synthesis responses and diminish catabolism in advanced-stage cachexia [8,11,13,15,16,177,180,181].

For safe and effective exercise, prescriptions must be individualized according to the patient’s capabilities. In cases of progressive cachexia, progressive exercise programs should be modified in response to decreased functional ability, and surveillance is necessary to ensure a balance between benefits and safety [13,15,16,180]. Guidelines provided by the European Society for Clinical Nutrition and Metabolism (ESPEN) and the European Society for Medical Oncology (ESMO) advocate a comprehensive approach to the treatment of CAC [182,183]. These approaches include dietary and exercise interventions, pharmacotherapy to treat inflammation and metabolic alterations, and psychosocial support. ESPEN and ESMO encourage moderate physical activity, combining both resistance and aerobic exercises, to contribute to the maintenance of muscle mass and physical performance [182,183].

Digestive tract cancers are closely associated with severe and rapid cachexia, metabolic disturbances, and inflammation [20,22,25]. Patients suffer from major problems including anorexia, significant weight loss, and treatment toxicity, leading to a downward spiral of malnutrition and deconditioning [22,184]. However, exercise interventions appear to be safe and beneficial, and resistance training has proven to be particularly valuable in maintaining or improving muscle mass and strength in patients with gastrointestinal cancer undergoing chemotherapy or radiotherapy [11,13,25,177,184,185]. Studies involving patients with pancreatic and esophageal cancers have shown that structured resistance training not only mitigates muscle wasting and loss but can actually lead to an increase in lean body mass, even in the setting of aggressive anticancer therapies such as chemotherapy and radiotherapy [25,184,185].

Importantly, these interventions have also improved patients’ tolerance to cancer therapies, which indirectly contributed to enhanced treatment efficacy and potentially improved survival outcomes [25,185]. However, adherence to such interventions is poor because of fatigue and gastrointestinal complaints [25,168,177,184,185]. Successful management should include a comprehensive, individualized multimodal approach that includes nutritional support, symptom control and exercise [9,10,177].

In some patients, particularly those with refractory cachexia, conventional exercise is not possible owing to physical frailty or medical comorbidities. In these cases, other forms of therapy should be considered. A few methods, such as neuromuscular electrical stimulation (NMES) or passive training, can mimic the effects of exercise by providing muscle contractions in immobilized patients [186]. These approaches have shown promise in improving muscle tone and strength in other settings, and can be modified for cachectic patients who are unable to exercise actively. Moreover, novel rehabilitative technologies such as robotic exoskeletons and assisted cycling devices enable patients to engage in physical activity, even those with limited capacity [186]. These new procedures expand the options for providing exercise-like benefits when standard exercise is not feasible.

### 3.2. Molecular Mechanisms of Action of Physical Exercise in Cancer Cachexia

Systemic inflammation plays a vital role in the pathogenesis of cachexia, and pro-inflammatory cytokines play a crucial role in this process and have been implicated in a variety of health problems, including anorexia, elevated metabolic rate, lipolysis, and proteolysis [34]. Exercise has been shown to have anti-inflammatory properties in animal models by increasing the expression of anti-inflammatory cytokines and potentially inhibiting TNF-α-induced lipolysis, which may attenuate the loss of adipose tissue observed in cancer cachexia [120,187].

Several studies using various animal models of CAC have demonstrated the anti-inflammatory effects of physical exercise. Lira et al. [188] reported that eight weeks of moderate treadmill running at 60% VO_2_max resulted in decreased levels of TNF-α, IL-1β, and IL-6 proteins in retroperitoneal and mesenteric fat in Walker-256 tumor-bearing rats. This training protocol enhanced the anti-inflammatory IL-10/TNF-α ratio, indicating anti-inflammatory transition. Trained rats exhibited a ten-fold reduction in tumor volume, partial correction of body weight loss, and improved plasma lipid profile, which was associated with peripheral anti-inflammatory effects and amelioration of systemic disease [188].

In a study by Li et al. [189], low-intensity swimming exercise conducted twice daily for four weeks in mice bearing CT-26 tumors reduced intramuscular TNF-α, IL-6, and IL-1β levels while shifting the IL-10/TNF-α ratio toward IL-10. This finding also indicates the anti-inflammatory properties of exercise in cachexia, followed by a reduction in macrophage infiltration. Shamsi et al. [190] have investigated the effects of aerobic interval training in 4T1 breast in cancer-bearing mice with cachexia. They observed that exercise training increased the IL-10/TNF-α ratio and IL-15 expression in skeletal muscle.

The anti-inflammatory effects of exercise are particularly relevant in GI cancers such as pancreatic cancer, where IL-6-driven cachexia is often severe. Disrupting the IL-6–STAT3–proteolysis axis with exercise or related interventions is a promising strategy [3,169]. Muscle wasting in cachexia reflects a marked imbalance in muscle protein turnover, with rates of breakdown greatly exceeding protein synthesis rates. Exercise, particularly resistance exercise, effectively counteracts this process by targeting key molecular pathways that regulate muscle protein synthesis and degradation [191].

Recent studies have demonstrated that even with short-term voluntary exercise in the colon-26 adenocarcinoma (C26) mouse model of colorectal cancer, tumor-bearing mice maintained normal rates of muscle protein synthesis and fiber size compared with sedentary tumor controls [192].

Animal studies have demonstrated that exercise training stimulates muscle protein synthesis and activates anabolic pathways such as the IGF-1/PI3K/Akt/mTOR signaling cascade. Puppa et al. [169] showed that treadmill exercise prevented suppression of muscle mTOR signaling in mice model of cancer cachexia. Interestingly, Sato et al. [193] observed that High-Frequency Stimulation (HFES) in female mice activated muscle protein synthesis via mTOR signaling, and repeated bouts of contraction attenuated cancer-induced muscle mass loss. Exercise-induced insulin-like growth factor 1 (IGF-1) promotes muscle hypertrophy via this pathway. The critical downstream effect involves Akt phosphorylation and the inhibition of FoxO transcription factors. FoxO proteins upregulate muscle-specific E3 ubiquitin ligases, such as MuRF-1 and MaFbx, which are important components of the ubiquitin-proteasome system (UPS) responsible for protein degradation. Exercise effectively reduced protein degradation via the UPS by suppressing FoxO activity [191,192]. Furthermore, exercise upregulated peroxisome proliferator-activated receptor gamma coactivator 1-alpha (PGC-1α), which is essential for increasing mitochondrial biogenesis and function. PGC-1α inhibits FoxO activity, further reducing muscle atrophy-related gene expression [191].

Exercise has a normalizing effect on cachexia, where excessive protein degradation driven by the UPS and autophagy-lysosomal pathways plays a dominant role. Wasted muscles exhibit elevated levels of E3 ubiquitin ligases (MAFbx/atrogin-1 and MuRF1) driven by FoxO and inflammatory signaling [191].

Studies in rodent models have shown that exercise attenuates tumor-induced increases in MuRF1/atrogin-1 expression and UPS activity. Low-intensity aerobic exercise in cachectic rats prevented muscle atrophy by downregulating UPS components and simultaneously activating mTOR, indicating a shift towards protein synthesis [194]. Endurance training in mice reduces levels of TWEAK and prevents NF-κB activation in the cachectic cardiac myocardium [36].

Exercise training also prevents excessive autophagic degradation in healthy muscle tissues. In a rat model of carcinoma cachexia, aerobic exercise restored autophagy markers to normal levels and improved muscle homeostasis [194]. Combined aerobic and resistance training in mice with colon carcinoma increased muscle mass, partly by restoring autophagy balance, reducing overactivation, and improving mitochondrial function [174]. The exercise-induced increase in PGC-1α also contributes to FoxO3 inhibition, thereby blunting muscle autophagy and UPS gene expression [191].

In summary, exercise counteracts muscle wasting in cachexia by simultaneously promoting muscle protein synthesis via the IGF-1/PI3K/Akt/mTOR pathway and suppressing excessive protein degradation mediated by the ubiquitin-proteasome and autophagy-lysosomal pathways [191]. This is achieved through mechanisms that include inhibition of FoxO transcription factors by Akt and PGC-1α, downregulation of E3 ubiquitin ligases, interference with inflammatory and pro-catabolic signaling (such as NF-κB), and beneficial modulation of autophagy to remove damaged components without causing excessive tissue breakdown. Exercise helps restore protein homeostasis and preserves muscle mass in cachexia by favorably influencing key molecular targets and pathways [191].

Cancer cachexia is closely associated with the increased production of reactive oxygen species (ROS) and oxidative damage, which significantly contribute to muscle wasting [195]. Wasted muscles often display mitochondrial dysfunction, characterized by reduced mitochondrial content, impaired oxidative metabolism, increased mitophagy, and oxidative damage, resulting in fatigue and decreased exercise capacity. This is exacerbated by chronic inflammation and is induced by tumor ROS burden, which can damage cellular components and activate catabolic signaling [195].

In addition to preserving muscle, exercise likely confers benefits to bone in the cachectic setting. Weight-bearing aerobic exercise and resistance training both provide mechanical stimuli that promote bone formation and strength [196,197,198]. Although direct studies of exercise on bone in cachectic animals are limited, it stands to reason that the bone loss seen in sedentary tumor-bearing mice would be less severe with physical activity (as is the case in osteoporosis models) [113]. Resistance exercise, in particular, can increase osteoblast activity and bone mineral density in healthy and osteopenic conditions, and may similarly mitigate cancer-associated osteopenia. Moreover, exercise’s anti-inflammatory effects could reduce the chronic cytokine milieu that drives osteoclast activation in cachexia [103].

In addition to its beneficial effects on preserving muscle mass, physical activity can also have a positive effect on bone tissue in cachexia. Both aerobic exercise with a weight-bearing component and resistance training provide important mechanical stimuli that stimulate osteogenesis and improve the structural strength of bone [196,197,198]. Although direct experimental data on the effects of exercise on the skeletal system in animal models of cachexia are limited, it is reasonable to speculate that the loss of bone mass observed in sedentary mice with tumors would be less severe with exercise intervention, analogous to the effects observed in models of osteoporosis [113,199]

Specifically, resistance training has shown potential to increase osteoblast activity and improve bone mineral density in both healthy subjects and osteopenic patients. Therefore, it is conceivable that similar mechanisms may alleviate cancer-related osteopenia [2,3]. Moreover, the anti-inflammatory effects of exercise may limit the chronic cytokine environment that promotes osteoclast activation and increased bone resorption in cachexia [5].

Exercise training may improve mitochondrial quality and reduce oxidative stress [175,200]. Aerobic exercise promotes mitochondrial biogenesis via the AMPK–PGC-1α pathway, increases muscle oxidative capacity and endurance, and counteracts tumor-induced mitochondrial loss [175]. Exercise-induced increases in antioxidant enzyme expression, reduction in muscle ROS, and oxidative damage likely interfere with ROS-driven proteolysis and apoptosis in cachexia-affected muscles. Exercise enhances cellular resilience and energy metabolism by restoring mitochondrial function, redox homeostasis, and critical disruption in cachexia [175,200].

It is well documented that regular physical exercise increases the antioxidant capacity of skeletal muscle and other tissues. Endurance (aerobic) exercise training has been repeatedly shown to increase the activity and/or expression of key antioxidant enzymes—notably SOD (both cytosolic CuZn-SOD/SOD1 and mitochondrial Mn-SOD/SOD2), GPx, and sometimes catalase, in trained muscles [201]. Six months of moderate-intensity aerobic training in humans increased total muscle SOD, CAT, and GPx activity by approximately 31%, 57%, and 51%, respectively [202]. Another study found that moderate endurance training increased muscle SOD2, GPx1, and peroxiredoxin-5 activity by 37–66% [203].

Preclinical studies support these findings, exercise in rodents of sufficiently high intensity (>50% VO_2_max) and duration (>30 min per session) typically produces significant increases (of the order of 20–100% or more) in SOD1, SOD2, and total GPx activity in skeletal muscle [204]. Research results indicate a dose–response relationship, whereby stronger training stimuli produce a stronger response than lower-intensity or short-duration exercise [204,205].

Resistance exercise may also enhance antioxidant defenses. Although animal studies are scarce, available data indicate that resistance training also increases the activity of antioxidant enzymes in muscles [201]. In rats, resistance exercise led to higher activity of total SOD (especially SOD1) and GPx in skeletal muscle, but catalase was not consistently changed. In humans, numerous studies in various populations also have shown that resistance training increases levels of antioxidant enzymes [206,207].

There is a difference in the acute and chronic effects of physical exercise on oxidative stress. While acute, intense physical exercise can transiently increase ROS production, regular training increases antioxidant defenses, mitigating oxidative stress over time. Repeated bouts of PA produce a sustained increase in antioxidant capacity, which not only protects cells from exercise-induced ROS but may also provide resistance to other oxidative challenges [201].

A recent human meta-analysis showed that regular exercise improves redox balance by augmenting antioxidant defenses and reducing pro-oxidant parameters, which consequently reduces oxidative stress and its role in muscle catabolism and apoptosis [208].

Antioxidant adaptations to exercise are directly relevant to cancer cachexia because they help break the vicious cycle between oxidative stress and muscle catabolism. Muscle affected by cachexia is exposed to oxidative stress due to tumor and host factors, including inflammatory cytokines and tumor-derived oxidants, as well as mitochondrial dysfunction [181]. Oxidative stress not only directly harms skeletal muscle but also initiates pathways that lead to muscle breakdown; the activation of NF-κB and p38 MAPK by ROS leads to the increased expression of atrogin-1 (MAFbx) and MuRF1, which promote protein degradation [209]. Additionally, ROS activates FOXO3, leading to the transcription of autophagy-related genes, contributing to the loss of muscle proteins and organelles through autophagy/mitophagy [175]. Furthermore, oxidative stress and proinflammatory cytokines mutually reinforce each other. ROS can activate inflammasomes and NF-κB to produce pro-inflammatory cytokines, while TNF-α and IL-6 can stimulate increased ROS production in muscle and other tissues [175].

Exercise training disrupts the cachexia cascade through the upregulation of muscle antioxidant enzymes, which reduces chronic oxidative stress [175]. Ballarò et al. [175] in study on mice with colon adenocarcinoma (C26) tumors showed that moderate endurance exercise can modify redox homeostasis and reduce cachexia in tumor-bearing animals. In that study, sedentary animals developed the expected cachectic symptoms, which included substantial muscle atrophy and strength decline together with elevated muscle ROS levels, protein carbonylation, and activated proteasome and autophagy pathways in muscle tissue [175]. The effects of muscle wasting and weakness were minimized in tumor-bearing mice that exercised moderately. The exercised tumor-bearing mice maintained their muscle mass and grip strength, while showing decreased markers of oxidative stress and enhanced antioxidant capacity in their muscle tissue. They demonstrated better mitochondrial health through increased muscle mitochondrial content than sedentary cachectic mice, which exhibited signs of mitophagy and mitochondrial loss [175].

In an additional study, Ballarò et al. [210] expanded these findings by exploring the role of exercise in the context of cancer and chemotherapy. The study revealed that moderate exercise in mice helped protect their muscles from mitochondrial atrophy and damage, which are the combined effects of cancer and chemotherapy [210].

The results of human studies confirmed the findings of animal studies and provided important information for clinical practice [211]. Although most human studies have shown that exercise programs for cancer patients effectively increase antioxidant levels while reducing oxidative damage, some studies have been inconclusive [211]. The results obtained in patients appear to depend on the type and stage of cancer as well as on the specific measures used [211].

## 4. Exerkines: Molecular Mediators of Exercise-Induced Systemic Adaptations

Several studies have provided convincing evidence that physical activity has beneficial health effects. Our knowledge of the impact of physical exercise on health has recently been enriched by the introduction of exerkines (Figure 1). These signaling molecules, including peptides, metabolites, and RNAs, are released into the blood by various organs during acute and chronic exercise. Exerkines act as endocrine, paracrine, and autocrine messengers in distant tissues and enhance cardiovascular, metabolic, immune, and neurological health [157,212].

**Figure 2:**
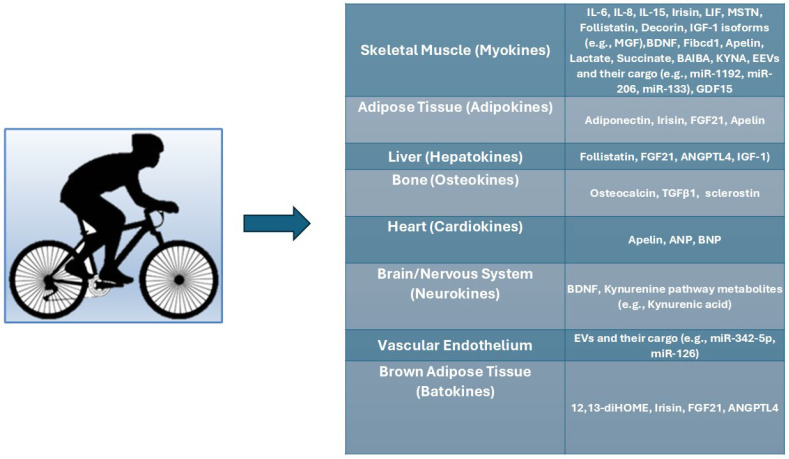
Exercise-induced release of exerkines from multiple tissues. IL-6—interleukin-6; IL-8—interleukin-8; IL-15—interleukin-15; LIF—leukemia inhibitory factor; MSTN—myostatin; MGF—mechano growth factor (IGF-1Ec); IGF-1—insulin-like growth factor-1; BDNF—brain-derived neurotrophic factor; Fibcd1—fibrinogen C domain–containing 1; BAIBA—β-aminoisobutyric acid; KYNA—kynurenic acid; EEVs—exercise-induced extracellular vesicles; miR—microRNA; GDF15—growth differentiation factor 15; FGF21—fibroblast growth factor 21; ANGPTL4—angiopoietin-like 4; TGFβ1—transforming growth factor beta-1; ANP—atrial natriuretic peptide; BNP—B-type natriuretic peptide; 12,13-diHOME—12,13-dihydroxy-9Z-octadecenoic acid. Arrow indicates the release of exercise-induced signaling molecules (exerkines) from different tissues during physical activity.

Exerkines are secretory factors that are produced by organs in response to physical activity (Figure 2). This definition encompasses a variety of signaling molecules that are produced during and after exercise to communicate between cells and tissues, organs, and systems [157,212,213,214]. The term “exerkine” was first coined in 2016, although exercise-induced factors such as lactate have been known for over a century [215,216]. Research on exercise-induced factors gained momentum in the early 2000s with the identification of myokines, cytokines, and peptides released by the muscle during contractions [217,218]. This concept has expanded since then, as many other organs (heart, adipose tissue, liver, bone, etc.) also release signaling factors during exercise [157,212,213,214]. The pool of circulating exerkines comprises several organs and tissues, including skeletal muscle (myokines), heart (cardiokines), liver (hepatokines), white adipose tissue (adipokines), brown adipose tissue (batokines), bone (osteokines), and neurons (neurokines). These molecules play a significant role in the health benefits of exercise through their effect on metabolic regulation, neuroprotection and muscle adaptation [157,212,213,214].

Exerkine secretion occurs in several ways. Some exerkines, particularly peptides and proteins that contain a signal peptide, are released by the classical process of exocytosis, which involves the endoplasmic reticulum and Golgi apparatus [219]. However, other exerkines, particularly those lacking signal sequences or unstable in the extracellular environment, are packaged into extracellular vesicles (EVs). EVs, such as exosomes (30–140 nm) and microvesicles (100–1000 nm), are membrane-bound vesicles that contain a wide range of biomolecules, including peptides, nucleic acids (mRNA and miRNA), and metabolites [220,221]. EVs are released in response to acute and chronic exercise, suggesting that they play a crucial role in interorgan communication and systemic responses to physical activity. Vesicles can interact with target cells through different mechanisms, such as receptor activation, direct fusion with the plasma membrane, or endocytosis, to deliver their contents and influence cellular processes [219,220,221].

Exerkines have many systemic effects, and can affect various organs and physiological processes. They facilitate inter-organ communication and crosstalk between organs, and contribute to metabolic health, immune function, and tissue repair. For example, muscle-derived exerkines can affect glucose metabolism in the liver and adipose tissues, whereas adipokines can affect muscle insulin sensitivity [157]. Moreover, exerkines have been found to have neuroprotective effects, some of which can cross the blood–brain barrier and affect learning, memory, neurogenesis, and synaptic plasticity [157,222]. Studies using animal models have shown that exerkines can mediate the cognitive benefits of exercise, reduce brain inflammation, and improve the skin health. The heart, liver, gut, endocrine, and immune systems are all affected by exerkines released during and after physical activity [223].

The release and effects of exerkines are strongly related to the characteristics of the exercise stimulus, including type, intensity, and duration. Low-to-moderate-intensity training increases apelin levels, which enhance protein synthesis, and HIIT rapidly increases circulating IL-6 levels. Resistance training reduces myostatin release, a negative regulator of muscle growth. Endurance exercise and HIIT elicit different exerkine responses, and exercise duration also determines the prolonged release of specific factors. Different exercise modalities, intensities, and durations elicit different patterns of exerkine secretion, which reflects the intricate relationship between exercise and exerkines [223,224].

The exerkine response differs between acute and chronic exercise. Acute exercise induces metabolic homeostasis and maintains responses through balanced pro- and anti-inflammatory mechanisms. A single bout of exercise increases the levels of cytokines including IL-6, IL-8, and IL-10 [214,225]. Chronic exercise or regular training results in long-term metabolic adaptations and involves sustained changes in exerkines, such as irisin and adiponectin, while decreasing systemic inflammation, leading to lower baseline levels of pro-inflammatory cytokines, including IL-6 [214,225]. miRNA profiles change dynamically; miR-146a and miR-221 decrease immediately post-exercise but rebound within hours, whereas miR-1 remains elevated for up to three hours, suggesting distinct roles in recovery versus adaptation [226]. The cumulative advantages of regular physical activity are partly due to repeated acute responses to exercise [214,225]. Knowledge of exerkines is essential to reveal the molecular basis of exercise as a therapeutic intervention.

One interesting aspect of exerkine research is how it reveals a network of organ crosstalk during exercise. Different organs contribute to different signals and each target tissue responds in a unique manner. Several organ-specific perspectives and differences have emerged [219].

As the largest organ by mass engaged during exercise, muscle is the predominant source of many exerkines [157,227,228]. Muscle contraction-induced myokines (such as IL-6, IL-8, IL-15, and irisin) typically mobilize energy and stimulate muscle adaptation. Muscle-secreted exerkines often have autocrine effects; they also help the muscle to remodel itself for future exercise. For instance, muscle-derived IL-6 not only enters the circulation to affect the liver and fat but can also provide feedback to muscles to boost glucose uptake during exercise [229,230]. Similarly, myokines, such as VEGF and FGF2, are released from the muscle to promote the formation of new blood vessels in the muscle itself, thereby improving endurance capacity [231,232]. A unique feature of muscle is its fiber-type composition; different types of exercise (endurance vs. strength training) may elicit different myokine profiles [233]. Endurance exercise tends to release more oxidative and angiogenic metabolic factors, whereas resistance exercise tends to release more anabolic and growth factors. However, there is an overlap, and many myokines (such as IL-6) respond to both modes [219,233].

Adipose tissue is not only a fat storage depot but also an active endocrine organ that communicates with the muscle, liver, and brain by releasing adipokines [234]. During exercise, adipose tissue itself can produce exerkines or respond to them. Adiponectin, an adipokine, increases in the bloodstream after exercise in humans [153]. Adipose tissue also sends inflammatory signals, but exercise shifts these signals towards an anti-inflammatory profile [235]. Adipose tissue is a target for many muscle exerkines. Among them, IL-6, irisin, BAIBA, and KYNA act on white fat to promote fat burning [235]. In rodents, exercise-conditioned fat shows “browning”—higher mitochondrial content and energy use—driven by such exerkines. In humans, the evidence for fat browning during exercise is mixed and a matter of debate [236]. Exercise makes adipose tissue healthier and more metabolically active, and both adipose tissue-derived and incoming signals play a role in this remodeling process [219].

The central metabolic role of the liver is that it influences and is affected by exercise signals. The liver releases hepatokines, such as FGF21, during exercise and angiopoietin-like 4, which helps regulate plasma triglycerides when the energy demand is high [157,227]. Proteomic studies have shown that exercise alters the profile of the proteins secreted by the liver. The liver is also a key target for other exerkines. IL-6 stimulates the liver to produce glucose and ketones to fuel muscle during prolonged exercise. Similarly, signals from the muscle prompt the liver to increase antioxidant production, improve fat processing, and protect against fatty liver disease [237]. Animal models have shown that when certain signaling pathways are blocked, exercise fails to improve liver health. This crosstalk between organs is vital for metabolic homeostasis during exercise [219,238,239].

The bone-derived osteokine osteocalcin can act on muscles [240]. Bone is also a recipient of exerkines; for example, muscle-derived irisin acts on bone to stimulate new bone formation, and BAIBA protects bone cells from oxidative stress [241,242,243].

The brain releases its own cytokines and responds to peripheral tissues [213]. During exercise, the pituitary gland and brain structures increase the production of factors such as brain-derived neurotrophic factor (BDNF), which supports neuronal growth and cognitive function. Blood BDNF levels increase after exercise and are believed to contribute to the mood and cognitive benefits associated with regular activity [213]. The discovery that muscle PGC-1α affects the kynurenine pathway (diverting a stress metabolite away from the brain) suggests an indirect exerkine effect from muscle to the brain that can combat depression [244]. Interestingly, a protein called cathepsin B, released from muscles during exercise, has been shown to cross the brain and stimulate neurogenesis in animal models, offering another link between muscle movement and brain health [213,245]. A fascinating observation in humans is that some hormones appear in the circulation, but decrease in the cerebrospinal fluid during exercise. For instance, one study noted that adiponectin decreases in the CSF even as it rises in the blood, hinting at regulated trafficking that might affect appetite or neuroendocrine responses [246]. The nervous system is both the source and the target of exerkine signals, integrating the body’s response to exercise [219,247].

The heart and blood vessels also communicate during exercise. The heart secretes natriuretic peptides (ANP and BNP) in response to increased exercise load, which helps reduce blood pressure and mobilize fat, which could be considered cardiokines [157,248].

During exercise, endothelial cells release more nitric oxide (NO) and EV-packed miRNAs (such as miR-126 and miR-342-5p), which aids in vascular adaptation and cardiovascular protection [249]. Evidence suggests that an exercised heart can release vesicles or proteins that benefit other organs, although disentangling the contributions of the heart and muscles is challenging in humans [157,248]. In contrast, muscle-released exerkines can act on the heart to improve its function and stress resistance [250]. Thus, the cardiovascular system participates in systemic crosstalk, ensuring that circulation and perfusion meet the body’s demands and that protective signals reach where they are needed [219].

### 4.1. Protein and Peptide Exerkines

Peptide and protein exerkines are a large group of biologically active substances, including cytokines, growth factors, hormones, and other proteins that are increased by physical exercise (Table 2). The primary source is skeletal muscle; however, other tissues such as fat, liver, heart, and bone can also secrete these substances after exercise [157,219].

#### 4.1.1. IL-6

One of the first myokines to be discovered is Il-6. Its concentration in the bloodstream increases dramatically immediately after exercise, particularly after intense or long-duration physical activity [217,251,252]. IL-6 is released from muscle fibers during contraction and affects numerous tissues in the body. During acute exercise, IL-6 acts as a metabolic regulator that stimulates lipolysis, gluconeogenesis, insulin-stimulated glucose uptake, and fat oxidation. Animal studies have shown that the inhibition of IL-6 signaling reduces exercise-induced lipolysis and fat loss, suggesting an important metabolic role. IL-6 has also been suggested to play an important role in exercise-induced immune cell redistribution, thereby enhancing overall immune surveillance [253].

Elevated plasma IL-6 levels are observed in pancreatic cancer and actively secrete IL-6 (upregulated up to 14-fold in circulating immune cells) and other IL-6 family cytokines, which correlate with weight loss and poor survival [254,255,256]. These cytokines activate catabolic signaling in muscle; IL-6 and TNF-α trigger the JAK/STAT3 pathway and NF-κB, which in turn upregulate muscle ubiquitin-proteasome system components (for example, E3 ligases MuRF1/TRIM63 and atrogin-1/MAFbx) and autophagy genes, leading to muscle wasting [254,255,256]. NF-κB activation (exacerbated by tumor factors and even chemotherapy) drives proteasomal breakdown and can suppress myogenic regulators such as MyoD, impairing muscle regeneration [254,255,256]. IL-6 plays a paradoxical role; acute IL-6 surges from exercising muscle have anti-inflammatory and anabolic effects, whereas chronically elevated IL-6 (as in cachexia) becomes detrimental [18]. Chronic IL-6 exposure suppresses muscle protein synthesis and activates proteolysis pathways; in cachectic patients, high IL-6 levels are associated with low IGF-1 levels and impaired AMPK/mTOR signaling, resulting in atrophy of muscle metabolism [18,255]. Consistent with this, blocking IL-6 signaling has been shown to attenuate cachexia. For example, trials of IL-6 or IL-6 receptor antibodies (clazakizumab and tocilizumab) in gastrointestinal cancers have shown reduced IL-6 levels, inhibited muscle loss, and improved albumin levels without accelerating tumor growth [255]. Recent studies in pancreatic cancer further emphasize the central role of IL-6, showing that an IL-6 trans-signaling loop between the tumor, fat, and muscle drives progressive wasting in this model [4].

The short-term exercise-induced IL-6 response creates an anti-inflammatory state by stimulating interleukin-10 and IL-1 receptor antagonist production and concomitantly reducing the pro-inflammatory cytokine TNF-α [253]. The effects of IL-6 depend on its specific context. Acute exercise-induced elevation of IL-6 is beneficial, but prolonged resting IL-6 elevation leads to an increased risk of cardiovascular disease and metabolic inflammation [253]. In cancer, IL-6 primarily exerts proinflammatory effects. Regular exercise over a longer period leads to lower basal levels of IL-6 along with other inflammatory markers in various chronic diseases, allowing for differentiation between short-term and long-term responses [253,257,258,259].

The pleiotropic nature of IL-6 may be due to the existence of two signaling pathways [253]. The “classical” mode of action of IL-6 is through binding to a cell membrane receptor (mbIL-6R), which is expressed on several cell types such as hepatocytes, monocytes-macrophages, lymphocytes, and skeletal myocytes. It is widely accepted that IL-6 is a major signaling pathway during exercise [253]. In contrast, trans-signalling is driven by the binding of IL-6 to a soluble form of its receptor (sIL-6R), generating a complex with a much longer half-life that is capable of signaling in all cell types. This trans-signalling mediates the functions of IL-6, which is associated with inflammation and constitutes the major pathway in pathological situations [253]. Therefore, IL-6 released in pathological situations cannot be considered as an exerkine because it is not released during exercise.

Released as a transient myokine during exercise, skeletal muscle IL-6 participates in a feedback loop with osteocalcin. Osteocalcin, a bone-secreted osteocin, in turn increases IL-6 production in muscle during exercise, while IL-6 stimulates the synthesis of bioactive osteocalcin [260,261]. Conversely, under pathological conditions, such as cancer cachexia, chronic IL-6 signaling induced by the presence of a tumor can promote osteoclastogenesis and enhance bone resorption. This phenomenon emphasizes the crucial importance of the biological context, duration, and mode of activation of the IL-6 signaling pathway, classical versus trans-signalling, for its impact on bone homeostasis [253,262].

#### 4.1.2. Leukemia Inhibitory Factor

Leukemia Inhibitory Factor (LIF) is a another pleiotropic cytokine of the IL-6 family that has emerged in the last decade as a key contributor to cancer cachexia, particularly muscle loss [263,264,265]. Paradoxically, LIF, which promotes muscle catabolism in malignancy, supports muscle regeneration and growth under healthy or exercising conditions [263,266,267]. During and post-exercise, the skeletal muscle synthesizes and secretes LIF. Studies have shown that LIF mRNA expression is strongly upregulated in human skeletal muscle following aerobic and resistance exercises, and resistance exercise is particularly substantial. LIF is secreted from both cultured muscle cells in vitro and intact cells in vivo, confirming its role as a myokine/exerkine [266,267,268,269]. Despite the marked increase in intramuscular LIF, its circulating levels do not appear to increase significantly post-exercise. LIF seems to act locally via autocrine/paracrine signaling in muscle tissue, binds to receptors on satellite cells, stimulates their proliferation, and promotes muscle regeneration and adaptation to exercise [266,267,268]. In mouse models, the delivery of recombinant LIF to injured muscle (or overexpression of the LIF transgene) increases the rate of muscle fiber regeneration and functional recovery [266,267,268]. LIF-knockout mice show a diminished regenerative response to muscle injury, with fewer proliferating satellite cells and fewer regenerated fibers than wild-type mice [266,267,268].

It is now well established that tumors that secrete LIF can act systemically on skeletal muscles to drive atrophy. In a landmark study in mice using the C26 colon carcinoma cachexia model, Seto et al. [265] identified LIF as a necessary and sufficient factor for muscle wasting. Conditioned medium from C26 tumor cells (which secrete high LIF) causes severe atrophy of cultured myotubes and robust STAT3 activation. Neutralization of LIF with specific antibodies abolished this atrophic effect, whereas blocking other cytokines (IL-6 or OSM) had no effect [265]. LIF levels were markedly elevated in the C26 tumor milieu, whereas other known wasting factors (IL-6, TNF-α, myostatin) were not, indicating that LIF is the key wasting cytokine in this model [265]. In vivo, C26 tumor-bearing mice showed an increase in circulating LIF that preceded and exceeded the increase in IL-6 [265]. Muscle wasting and STAT3 phosphorylation in these mice were dramatically reduced by JAK2 inhibitors or LIF antibody [265]. Together, these findings demonstrate that LIF produced by cancer cells acts directly on muscles to cause atrophy, primarily through JAK2/STAT3 signaling [265]. Subsequent studies have reinforced this mechanism in other animal tumor models and cancer types. For example, many human and murine tumor lines (including pancreatic, lung, and colon cancers) secrete LIF, and high LIF levels are associated with cachexia development in tumor-bearing mice [270]. Clinically, circulating LIF levels tend to be elevated in patients with advanced malignancies, and are thought to be a major contributor to cachexia-related weight loss and anorexia. Indeed, high serum LIF are often observed in patients with cachectic disease, which is associated with a poorer prognosis [264,271].

The explanation for this phenomenon, how LIF could mediate such opposing effects in muscles, was explained by the context in which LIF acts, its dose, and duration of action [264]. Under physiological conditions, LIF is a locally acting myokine released in response to exercise or minor trauma. In such situations, LIF does not enter the bloodstream in large amounts; therefore, its effects remain localized [263,264]. However, in pathological conditions such as cancer cachexia, LIF production becomes chronic, dysregulated, and systemic. Tumors can chronically secrete elevated levels of LIF into the bloodstream chronically [263,270]. Another critical factor for LIF is the concomitant presence of other cytokines and stress signals. In exercising muscles, the effects of LIF may be counterbalanced by growth factors (e.g., IGF-1) and the anti-inflammatory environment following exercise. In the case of cancer cachexia, LIF acts in concert with a number of other catabolic factors (IL-6, TNF-α, GDF15, etc.) that may amplify signals of muscle-wasting [264].

#### 4.1.3. Myostatin

The muscle-derived protein (MSTN) was the first identified myokine, although it was not initially known by its name [272]. MSTN, also known as growth differentiation factor 8 (GDF-8), is a member of the TGF-β family and is secreted by the muscle fibers. It binds to the activin type IIB receptor (ActRIIB) on muscle cells, activating a cascade of Smad and forkhead protein (FoxO) transcription factors that suppress muscle protein synthesis and promote protein degradation.

This signaling pathway activates the mTOR pathway and simultaneously upregulates atrophy-related genes. Myostatin also impairs the differentiation of satellite cells into new muscle fibers, and high myostatin levels limit muscle fiber size and number, and reduce the regenerative capacity of the muscle [273]. The FoxO pathway activated by MSTN causes muscle atrophy along with inhibition of skeletal muscle glucose uptake through GLUT4 reduction and AMPK deactivation. In effect, high myostatin levels limit muscle fiber size and number, and reduce the regenerative capacity of the muscles. Importantly, elevated myostatin levels have been linked to muscle wasting. Elevated myostatin levels in the muscles or blood are associated with conditions involving muscle loss, such as age-related sarcopenia, cancer cachexia, and heart failure [273,274]. Endogenous follistatin counteracts these effects by activating the Akt-mTOR pathway to stimulate protein synthesis, while functioning as a pro-hypertrophic signal [273,275].

Myostatin also suppresses osteoblastogenesis and promotes bone resorption. Genetic or pharmacologic myostatin inhibition increases bone mass or preserves bone in several models; importantly, targeting myostatin/activin protects both muscle and bone under atrophic conditions [276,277]. In fact, experimental blockade of myostatin/activin (such as with ACVR2B receptors traps) in tumor-bearing mice not only increases muscle mass but also prevents cachexia-associated bone loss, reinforcing the myostatin link in muscle–bone pathology [277].

Exercise has a strong effect on myostatin expression and activity. Unlike most myokines, myostatin levels typically decrease during exercise. Myostatin levels decrease during resistance training, reflecting their impact on protein synthesis [228]. A transient reduction in myostatin levels was observed within 24 h post-exercise after a single bout of high-load resistance exercise. In contrast, chronic resistance evokes more sustained adaptations, leading to strong reductions in resting levels of myostatin in both the blood and muscle, with elevated follistatin levels [228,273].

Aerobic or endurance exercise also modulates myostatin, although its effects can differ from those of resistance exercise in terms of magnitude and functional outcome. Several studies have shown that endurance training can reduce myostatin levels in the muscle and blood, which may contribute to improved muscle metabolism and maintenance [278].

Myostatin is often overexpressed in the muscles and plasma of patients with cachexia and in animal models [3]. However, results in humans are not entirely consistent, and some studies have reported only modest differences or even lower myostatin plasma levels in cachectic patients [38,279].

Importantly, some tumors can produce myostatin or related ligands. Recent clinical research in lung cancer found that a subset of resected tumors expresses high levels of myostatin, which correlates with significantly lower skeletal muscle mass in these patients [280]. In addition, high tumoral myostatin levels were associated with greater infiltration of tumor-associated macrophages and worse survival, suggesting that tumor-produced myostatin can have both systemic (muscle-wasting) and local (tumor microenvironment) effects [280].

#### 4.1.4. Activin A

In addition to myostatin, many cancers secrete activin A, a closely related TGF-β family ligand that uses the same ActRIIB receptor. Activin A is frequently elevated in cachectic patients and secreted by various cancer cells [3]. It likely contributes, alongside myostatin, to the overall ActRIIB-mediated catabolic signaling. Thus, in cancer cachexia, the total “myostatin signal” driving muscle atrophy may originate from a combination of host-derived myostatin (and activins) and tumor-derived factors.

#### 4.1.5. Follistatin

Conversely, follistatin, a muscle-derived exerkine that binds and neutralizes myostatin and activin A, is significantly reduced in cachexia [18]. Muscle-secreted proteoglycans, decorin, and follistatin are produced during resistance exercise to prevent myostatin from inhibiting muscle growth [281,282]. Follistatin was first identified through its involvement in folliculogenesis and activin inhibition, but it is now recognized as an exerkine produced by the liver and muscle tissues in response to exercise [283,284]. A combination of aerobic and resistance exercise training for 12 weeks elevated follistatin serum levels in elderly participants, resulting in improved muscle mass and metabolic function [283].

Follistatin mRNA delivery via nanoparticles has been established as a revolutionary approach to treating cachexia. Intraperitoneal delivery of lipid nanoparticles containing FST mRNA in ovarian cancer models decreased activin A levels, while preserving muscle mass and increasing survival when used with cisplatin. This approach utilizes body-produced follistatin to maintain myostatin inhibition over time, avoiding problems associated with recombinant protein therapies [285].

#### 4.1.6. Decorin

Decorin, a small leucine-rich proteoglycan, is found in the extracellular matrix of many tissues, including skeletal muscle. It plays a crucial role in various physiological processes, including collagen fibrillogenesis and the modulation of growth factors such as transforming growth factor-beta (TGF-β) and myostatin. Decorin also exhibits onco-suppressive properties by interacting with multiple receptor tyrosine kinases.

Evidence strongly suggests that decorin is an exercise-regulated myokine [281,286]. Studies have shown that decorin is released from contracting human myotubes, indicating that muscle contraction directly stimulates decorin secretion. Exercise significantly increases decorin levels in humans, and acute resistance exercise is particularly effective [233]. In both humans and mice, skeletal muscle decorin expression is elevated following chronic exercise training, suggesting that regular exercise induces long-term adaptations in muscle decorin production [233,281]. Furthermore, a single bout of whole-body vibration (WBV) stimulates the release of decorin into circulation, showing a pattern similar to traditional exercise, with circulating decorin concentrations being notably higher immediately following WBV [287]. High-intensity interval training (HIIT) has also been shown to significantly increase decorin levels in trained individuals compared with control groups [286]. These results suggest that different forms of exercise can stimulate decorin release, indicating that exercise regimens can be tailored to maximize their beneficial effects. The interaction between decorin and myostatin is crucial for their role in exercise and muscle hypertrophy. Decorin binds directly to myostatin and modulates its activity. By binding to myostatin in the extracellular matrix, decorin effectively blocks its inhibitory effects on myoblast proliferation, thereby promoting muscle hypertrophy [281,288]. Mechanistically, decorin increases the expression of pro-myogenic factors and decreases that of ubiquitin ligases involved in muscle atrophy [281,288].

Decorin may offer a potential treatment for cancer cachexia via multiple mechanisms [289]. Decorin acts by binding directly to myostatin and protecting muscle tissue from catabolic effects; however, it also exhibits onco-suppressive features and effects on the immune system [289,290,291]. Its anti-inflammatory activity is important for preventing muscle wasting associated with systemic inflammation in cancer cachexia [288,291]. Decorin is of particular therapeutic interest because of its synergistic action against myostatin-mediated muscle breakdown and inflammation in cancer cachexia [288,289,291]. In a mouse model of prostate cancer bone metastasis, systemic administration of an oncolytic adenovirus carrying the decorin gene (Ad.dcn) significantly inhibited cancer cachexia [292].

An imbalance between high myostatin and low follistatin levels contributes to unchecked muscle wasting. Therapeutically, this axis is attractive: experimentally knocking down or inhibiting myostatin not only blunts muscle atrophy but also reduces systemic inflammation (by inhibiting IL-6 and TNF-α levels). Simultaneously, follistatin therapy can promote muscle hypertrophy and mitigate atrophy in cachexia models [18].

#### 4.1.7. IGF-1

IGF-1 is a major growth factor that controls the anabolic and catabolic pathways in skeletal muscle, thus playing a key role in muscle growth, differentiation, and regeneration. IGF-1 is primarily produced in the liver and acts as a systemic growth factor; however, its various isoforms are released by skeletal muscle through autocrine and paracrine mechanisms [293,294]. Exercise affects IGF 1 in two ways: it raises hepatic IGF 1 secretion and simultaneously turns on local expression of muscle-specific splice variants that help muscle fibers and other organs adapt [295]. Human and animal data show that the magnitude and rate of these changes depend on exercise regimen, intensity, age, and metabolic health [296,297,298]. At the molecular level, IGF 1Ea sustains protein synthesis, IGF 1Eb and the stress-responsive IGF 1Ec, or mechano growth factor (MGF), activate satellite cells and remodel tissue, while circulating IGF 1 stabilizes glucose and lipid handling during and after workouts [293,295,298,299,300]. IGF-1 supports osteoblast survival/function and is positively associated with bone mineral density and lower fracture risk [301].

Growth hormone pulses during prolonged or repeated exercise stimulate hepatocytes to secrete IGF 1 into the blood, promoting metabolic balance and aiding recovery [294]. Acute endurance training can lead to transient increases in serum IGF 1 concentrations of 10–20% in trained adults, although responses vary with nutritional status, sex hormones, and circadian timing [297,302]. In older or obese volunteers, blunted liver function correlates with slower protein synthesis and weaker training gains [297,303].

Contracting fibers splice the IGF1 gene into multiple transcripts, the peptides of which act in autocrine, paracrine, and endocrine pathways once in the plasma [293,300]. Resistance exercise markedly increased IGF 1Ec/MGF mRNA levels within hours, reaching a peak at 24–48 h, whereas endurance exercise promoted IGF 1Ea expression and export [293,300]. These local pools reach neighboring fibers and immune cells more rapidly than liver-derived hormones, accelerating repair after damaging eccentric work [293,300].

IGF-1 stimulates skeletal muscle anabolism via the PI3K/Akt/mTOR and PI3K/Akt/GSK3β signaling pathways [304,305]. Activation of PI3K/Akt through this process leads to the inhibition of FoxO, which results in reduced expression of E3 ubiquitin ligases that regulate protein breakdown via the ubiquitin-proteasome system (UPS) [305]. Autophagy appears to be inhibited by IGF-1 signaling, which involves the mTOR and FoxO pathways [304]. IGF-1 stimulates satellite cell activation, leading to muscle hypertrophy and prevention of muscle atrophy. In many chronic disorders, IGF-1 levels are suppressed along with IGF-1R signaling, leading to muscle atrophy [304].

Circulating and muscle-specific IGF-1 levels are reduced in animal models of cancer cachexia (e.g., AH-130 hepatoma and C26 colon adenocarcinoma), correlating with the loss of muscle mass [306]. Hepatic IGF-1 production is also suppressed, which contributes to systemic deficiency [306]. In the Yoshida hepatoma rat model, low-dose IGF-1 (0.3 mg/kg/day) attenuated lean mass loss (−28.8 g vs. −41.4 g in controls), improved food intake/activity, and reduced mortality (HR = 0.45) [307].

#### 4.1.8. IL-15

IL-15 is another myokine released by muscles after exercise [308,309]. A recent meta-analysis revealed that acute exercise effectively increases IL-15 concentrations immediately and one-hour after exercise, regardless of whether the exercise is resistance or endurance [308]. In contrast, chronic exercise did not significantly affect IL-15 levels. However, endurance training in animal models of diabetes and long-term treadmill running increases IL-15 expression in skeletal muscle and consequently improves glucose tolerance and insulin sensitivity [310]. IL-15 stimulates the activation of signaling pathways associated with muscle growth and hypertrophy in response to exercise regimens. In addition to muscle health, IL-15 plays a role in regulating immune responses and is particularly relevant in pathological conditions such as cancer and autoimmune diseases [309].

IL-15 in cancer cachexia may have a dual role as a biomarker for muscle mass and as a potential preclinical drug candidate. However, its direct role in cachexia development in humans remains unclear. Basal IL-15 levels in patients with cachexia were not different from those in healthy controls [311]. In rodent models, IL-15 administration led to the inhibition of protein degradation pathways (e.g., the ubiquitin-proteasome system) and apoptosis and counteracted cachexia [312]. Clinical studies have shown that increased IL-15 levels are associated with improved muscle mass and reduced proteolysis in patients who regain weight. Patients with baseline IL-15 levels <2 pg/mL had poorer preservation of fat mass and weight loss, suggesting that IL-15 may serve as a prognostic biomarker [311]. The effects of IL-15 may depend more on local production in the muscle than on systemic levels, which current assays may not fully capture.

IL-15 may also influence bone, as it has been reported to promote osteoblast differentiation and bone mass in some contexts [313].

#### 4.1.9. Irisin

Irisin is a hormone-like peptide generated from the muscle protein FNDC5 after exercise. Irisin was initially identified as a peroxisome proliferator-activated receptor-γ coactivator-1α (PGC-1α)-dependent myokine. It is released into the bloodstream by cleavage of skeletal muscle membrane-associated type III fibronectin domain protein (FNDC5). This process occurs in response to exercise or muscle shivering, leading to browning of white adipose tissue and regulation of thermogenesis [314,315,316].

Irisin levels increase during endurance exercise and act on adipocytes to increase energy expenditure and heat production. It may also affect bones; irisin has been observed to stimulate osteoblasts, which may explain how physical activity can strengthen bones. However, there were initially some concerns about measuring irisin levels in humans, as different assays yielded different results. However, many studies have detected increases in circulating irisin levels following physical exercise [315,316].

Studies investigating the relationship between irisin level and cancer cachexia have yielded conflicting results [113]. In a mouse model of gastric cancer-induced muscle cachexia, the expression of FNDC5 increased in adipose tissue, along with elevated levels of circulating irisin. Increased FNDC5 expression in white and brown adipose tissues of mice with experimentally induced gastric cancer may result from a cachectic effect [317]. Patients with cancer cachexia have been found to have higher irisin levels than weight-stable individuals. In contrast, irisin levels were lower in patients with colorectal and breast cancers than in healthy subjects [113].

In preclinical models, it was shown that irisin administration in mice led to an increase in cortical bone mass and strength. This mechanism is associated with activation of integrin αV-containing receptors located on osteoblasts and osteocytes, resulting in improved bone cell survival and modulation of the expression of sclerostin, an inhibitor of osteoblast activity [318].

#### 4.1.10. Fibrinogen C Domain Containing 1

Fibrinogen C Domain Containing 1 (Fibcd1), a myokine that regulates myofiber size, has been suggested to have a promising role in treatment of cancer cachexia. Graca et al. [319] identified evolutionarily conserved myokines that influence myofiber size, focusing on the mechanism of action and potential therapeutic applications of Fibcd1. Similar to Irisin/FNDC5, which also helps maintain myofiber size, Fibcd1 is released by proteolytic cleavage of its transmembrane form and is present in human plasma. They showed that local injection of recombinant secreted Fibcd1 reduces muscle fiber atrophy in the diaphragm muscle caused by cancer cachexia. They suggested that recombinant Fibcd1 could serve as a therapeutic agent to prevent muscle loss in cancer cachexia or to aid its recovery, particularly as Fibcd1 interacts with receptors on muscle cells but not on cancer cells, indicating that its use is unlikely to promote cancer growth, making it a potentially safe therapeutic target.

#### 4.1.11. Apelin

Apelin, a small peptide hormone, is produced by several tissues, including the heart, adipose tissue, vascular endothelium, lungs, kidneys, gastrointestinal tract, placenta, and reproductive organs [320,321]. Exercise significantly increases apelin secretion; therefore, the primary source of apelin secretion is skeletal muscle, although other tissues may also contribute. In particular, the vascular endothelium may contribute to apelin secretion in response to exercise-induced changes in the blood flow and shear stress. Interestingly, exercise elevates placental apelin expression during pregnancy, supporting maternal-fetal signaling [320,321,322]. Diakowska et al. [148] demonstrated significantly higher apelin levels, in patients with gastroesophageal cancer, particularly in cachectic individuals. However, there was no correlation between apelin levels and cachexia severity [148].

#### 4.1.12. FGF21

The liver, adipose tissue, and muscle primarily secrete FGF21, which increases following acute exercise [323,324,325]. The effects of chronic exercise on FGF21 levels are complex and may depend on the type of exercise, duration, and population. Recent meta-analyses have suggested that chronic exercise reduces circulating FGF21 levels in adults with metabolic disorders, potentially reflecting improved FGF21 sensitivity and metabolic health [326]. The hormone acts through specific receptors (FGFR1 with the co-receptor β-Klotho) in adipose and other tissues to increase metabolism [323]. The sensitivity of adipose tissue to FGF21 is increased by exercise because of an increase in the number of its receptors, which leads to improved glucose and lipid metabolism. In high-fat diet-fed mice, exercise training normalized FGF21 signaling in adipose tissue, which is crucial for metabolic improvements achieved with exercise [323,325]. In patients with cancer cachexia, FGF21 levels tend to be elevated, which may be a response to increased energy demand or the activation of brown adipose tissue [327]. Oost et al. [328] showed that FGF21 was crucial for fasting-induced muscle atrophy. In fasted control mice, isolated muscle mass decreased by 15–25% compared with that in fed control mice. However, deletion of FGF21 prevented muscle loss during fasting. The role of FGF21 in the atrophy process was further supported by the overexpression of FGF21 in muscles in vivo, which was sufficient to induce autophagy and resulted in 15% muscle loss [328].

#### 4.1.13. Growth/Differentiation Factor 15

Growth/Differentiation Factor 15 (GDF15) is a stress-responsive circulating cytokine that belongs to the transforming growth factor-β family, and is recognized as an exercise-induced cytokine with complex roles in metabolism, energy balance, and adaptation to exercise. GDF15 is a stress-induced cytokine that is normally expressed at low levels, but is highly upregulated in response to tissue damage, inflammation, oxidative stress, and cancer. Acute intense exercise significantly increases circulating GDF15 levels in humans. These increases are transient, peaking during and shortly after exercise before returning to baseline levels [329]. However, its origin is not clearly understood (the source may be different tissues) [330].

GDF15 is known to suppress appetite in the brain [331]. Elevated levels of circulating GDF15 are consistently found in patients with cancer cachexia and are correlated with anorexia, weight loss, and decreased survival [332]. In addition to appetite suppression, GDF15 directly induces muscle wasting. Experimental studies have shown that GDF15 increases the expression of muscle-wasting markers and decreases muscle fiber diameter. It also promotes muscle loss through pathways such as MAP3K11 activation and the Bcl-2/caspase-3 apoptotic pathway in muscle tissues [332]. This aligns with in vivo findings that neutralizing circulating GDF15 reverses cachexia-induced weight loss [333]

#### 4.1.14. Osteocalcin

Osteocalcin (OC), a protein derived from osteoblasts, has recently been classified as an exerkine. In mice, physical activity significantly increases serum osteocalcin, which contributes to the ability of muscles to absorb and utilize nutrients, thereby supporting physical activity [113]. In humans, serum osteocalcin levels, especially those of undercarboxylated osteocalcin (ucOC), a bioactive hormonal form, are elevated by acute exercise. Furthermore, persistent exercise over weeks increases the serum levels of osteocalcin and ucOC, which are associated with favorable metabolic profiles, such as reduced adiposity, enhanced glucose metabolism, and improved lipid profiles [334,335,336]. Remarkably, in mice and humans, OC levels decrease with age, and exogenous administration of OC restores exercise capacity and muscle mass in aged mice and increases exercise capacity in young animals. Osteocalcin plays a key role in ensuring optimal exercise performance in mice by participating in the osteocalcin-IL-6 signaling pathway activated during physical exercise; this axis may be weakened in states of cachexia, but its functionality can be restored through training intervention [261,337]. However, there are no data on whether OC plays a role in cancer cachexia [113].

#### 4.1.15. Brain-Derived Neurotrophic Factor

Brain-derived neurotrophic factor (BDNF) plays a key role in learning and memory in neural tissues; however, it also acts as a myogenic factor in skeletal muscles. Circulating plasma levels of BDNF are reduced in both neurodegenerative diseases and metabolic disorders such as obesity and type 2 diabetes. In humans, BDNF production is stimulated by skeletal muscle exercise, and resistance exercise, in particular, can elevate plasma BDNF levels [228,338]. BDNF promotes satellite cell proliferation and differentiation in skeletal muscle [339]. Modulation of BDNF expression is significantly altered in response to muscle damage, enabling satellite cell activity and proliferation [340]. In addition to its regenerative functions, muscle-derived BDNF improves energy metabolism by activating the AMPK–PGC1α pathway and consequently promoting fat and lipid oxidation [341,342]. Although there is no direct evidence linking BDNF to cachexia, it is speculated that this molecule could modulate the pathogenesis of muscle wasting in cachexia, as it is known to affect muscle maintenance and regeneration. Changes in BDNF expression or its signaling may have implications for muscle function and atrophy in cachexia [66].

#### 4.1.16. Angiopoietin-Like 4

Angiopoietin-like 4 (ANGPTL4), initially recognized for its role in lipid metabolism, has emerged as a critical mediator of inter-organ communication between the skeletal muscle, liver, and adipose tissue [343,344]. Acute bouts of exercise transiently elevate intramuscular or hepatic ANGPTL4, where it acts as an “exerkine” that coordinates lipid use and satellite cell activation. However, many tumors chronically oversecrete ANGPTL4, driving systemic inflammation, lipolysis, and muscle fat wasting, typical of cachexia [343].

ANGPTL4 is expressed in multiple tissues including the liver, skeletal muscle, adipose tissue, and kidneys. However, its release during exercise exhibits tissue-specific effects. Hepatic secretion dominates the systemic ANGPTL4 response, and measurement of arterial-to-venous differences across the hepatosplanchnic bed during acute exercise supports that the liver is the primary source of circulating ANGPTL4 [343]. In contrast, skeletal muscle contributes minimally to systemic ANGPTL4 during exercise, despite local upregulation of mRNA in both exercising and non-exercising muscles. This dichotomy suggests that ANGPTL4 has distinct paracrine and endocrine roles: locally in muscle, it modulates lipid uptake, whereas hepatic ANGPTL4 regulates systemic lipid trafficking and transport [343].

ANGPTL4 levels in plasma and tumors are markedly elevated in cachectic colorectal cancer patients and correlate positively with NF-κB, IL-1β, and MCP-1 levels in mesenteric adipose tissue, supporting a pro-inflammatory catabolic axis [345]. Single-cell sequencing of adipose stromal fractions from cachectic patients confirmed ANGPTL4 enrichment in inflammatory progenitor clusters [346].

Mouse models bearing pancreatic, lung, or colon tumors overexpressing ANGPTL4 exhibit accelerated fat browning, increased resting energy expenditure, and profound loss of muscle mass [347]. Genetic silencing or antibody neutralization partially restores body weight and muscle cross-sectional area without affecting tumor size [348].

Duration, compartmentalization, and proteolytic processing determine outcomes; short-lived, muscle-restricted full-length ANGPTL4 supports fuel redistribution and repair, whereas persistent circulating cANGPTL4 isoforms from tumors sustain lipolysis and endothelial leakage, magnifying catabolic inflammation [349].

### 4.2. Metabolic Exerkines

Exercise triggers the release of numerous metabolites and small molecules that, in addition to proteins, function as signaling agents (Table 3).

Metabolic changes in muscle cells during exercise lead to fuel burning, which generates various byproducts that enter the bloodstream and communicate with the body tissues. Modern metabolomics studies track hundreds of compounds in the blood to identify the metabolites that change during exercise. Circulating levels of lactate and other exerkines are significantly influenced by exercise intensity and duration. Studies have shown that vigorous-intensity exercise tends to elicit more pronounced metabolic changes and a greater release of exerkines than moderate-intensity exercise.

#### 4.2.1. Lactate

According to contemporary knowledge, lactate, historically considered a by-product of anaerobic processes occurring during intense exercise and responsible for muscle fatigue, has gained new and important meaning. It is currently considered a signaling factor referred to as lactomone [215,350]. Lactate, acting as an exerkine released from working muscles, is not only transported to the liver for conversion into glucose but also binds to receptors on the surface of fat cells and other tissues [215,350]. The interaction of lactate with fat cells increases the rate of lipolysis, and its action in the brain affects appetite control centers. Signals transmitted by lactate to the appetite centers of the brain cause a short-term reduction in appetite [215,219,350]. In addition, lactate affects angiogenesis and skeletal muscle adaptation through regenerative mechanisms by activating specific signaling pathways [215,219,350].

Recent studies have indicated that lactate elevation plays an essential role in both the initiation and progression of cachexia, demonstrating that lactate functions as a biomarker and a potential therapeutic agent [351]. Studies have established that the severity of cachexia in patients with cancer is directly correlated with circulating lactate concentration. A similar increase in lactate levels has been observed in mouse cancer models. Interestingly, an increase in lactate levels occurred before significant weight loss was observed [351]. Lactate infusion in tumor-free mice led to cachexia symptoms in a dose-dependent manner. This effect appears to be stereospecific since D-lactate infusion did not lead to the same effects [351]. The catabolic effects of lactate occur via activation of G protein-coupled receptor 81 (GPR81), which acts as a lactate sensor in adipose tissue owing to its high expression in this tissue. Binding of lactate to GPR81 activates distinct signaling pathways that activate RhoA/ROCK1, leading to activation of p38 mitogen-activated protein kinase (MAPK) [351]. This signaling pathway promotes the metabolic remodeling of adipose tissue, characterized by increased browning and lipolysis, and leads to skeletal muscle wasting and systemic hypercatabolic effects. Ablation of GPR81 in mice ameliorated lactate-induced adipose and muscle wasting [351].

#### 4.2.2. Succinate

The Krebs cycle (TCA cycle) intermediate succinate accumulates in muscles during exercise, particularly when muscle cells become acidic due to prolonged exertion. Studies on physical activity have unexpectedly revealed that working muscles selectively release succinate into the bloodstream [219,352]. This unique process involves pH-dependent protonation of succinate, which allows cellular release when the muscle pH decreases and interacts with distant tissues. Succinate binds to SUCNR1 receptors found in multiple cell types to initiate cellular responses. Muscle tissue uses locally produced succinate to stimulate immune and endothelial cells to remodel and form capillary [219,352]. Succinate has been suggested to act as an endocrine signal that modifies metabolic processes in the adipose tissue and liver cells. Succinate acts as a metabolite that links muscle metabolic activity to whole-body adaptive responses [219,352]. Recent studies have shown that succinate enhances SC myogenic capacity via SUCNR1 [353]. The HIIT murine model received 1.5% succinate supplementation, which resulted in a 46% increase in grip strength and a 37% increase in endurance, along with muscle growth and neuromuscular junction repair [353]. The muscle adaptation effects of succinate were eliminated when SUCNR1 was knocked out in SCs, demonstrating the necessity of this receptor for muscle adaptation. SUCNR1 activation leads to upregulation of protein kinase C eta (PKCη) and p38α mitogen-activated protein kinase (MAPK), which are essential for SC differentiation [353].

Although succinate promotes muscle repair by activating SCs, its pro-inflammatory effects may exacerbate cachexia [354]. SUCNR1 signaling in macrophages and lymphocytes increases IL-1β and TNF-α production and sustains muscle proteolysis [354]. SUCNR1 activation in regulatory T cells (Tregs) leads to reduced inflammation, but this effect is context dependent. The ultimate outcome of cancer cachexia is determined by the succinate concentration, receptor distribution, and tumor microenvironmental factors. Serum succinate levels in cachexia patients are positively correlated with CRP and IL-6 levels, indicating a predominant inflammatory effect of this molecule in CCs [354].

#### 4.2.3. β-Aminoisobutyric Acid

β-Aminoisobutyric acid (BAIBA): During exercise, skeletal muscles produce the small β-amino acid derivative, BAIBA, via valine breakdown. Metabolomic studies have shown that BAIBA is involved in browning of white adipocytes and improves glucose homeostasis [219,355]. BAIBA activates a receptor called MRGPRD in osteocytes (bone cells), leading to the protection and stimulation of bone development. In mice, BAIBA increases lipolysis while simultaneously increasing insulin sensitivity [355]. In humans, physical exercise increases BAIBA levels and improves markers of metabolic health in individuals with higher BAIBA concentrations [219,355].

However, the role of BAIBA in cancer cachexia has not been thoroughly investigated. The ability of L-BAIBA to improve muscle function and preserve bone mass suggests its ability to counteract the negative effects of cancer cachexia [113]. The effects of BAIBA on fatty acid oxidation and its anti-inflammatory actions may counteract catabolic signaling. BAIBA reduces muscle and bone loss [113,356]. In murine models of cachexia (hindlimb-unloaded mice), BAIBA supplementation preserved muscle and bone mass by protecting mitochondrial integrity and reducing ROS formation [356].

#### 4.2.4. Kynurenine Pathway Metabolites

The kynurenine pathway (KP) of tryptophan catabolism produces kynurenine (KYN) and kynurenic acid (KYNA), which act as signaling molecules beyond their traditional metabolic roles, including myokine activity as muscle-derived signaling molecules [357].

The KP pathway metabolizes approximately 95% of dietary tryptophan into bioactive metabolites that affect immune responses, metabolic processes, and the neurological system [357]. Indoleamine 2,3-dioxygenase (IDO1) and tryptophan 2,3-dioxygenase (TDO) catalyze the conversion of tryptophan into KYN and further into KYNA and QUINA and 3-hydroxykynurenine [358,359]. The neuroprotective agent KYNA blocks NMDA and α7 nicotinic receptors, but QUINA causes neurotoxicity through NMDA receptor overactivation [358,359]. KYN derivatives act on peripheral tissues to control immune tolerance, oxidative stress, and energy metabolism, with implications for cancer progression and cachexia. Skeletal muscles contain enzymes of the kynurenine pathway, including kynurenine aminotransferases (KATs), kynurenine 3-monooxygenase (KMO), and kynureninase, which convert kynurenine to kynurenic acid and other metabolites [357,358,359]. Exercise induces changes in skeletal muscle kynurenine metabolism, increases KAT expression, and increases blood kynurenic acid levels. Kynurenic acid, produced by muscles during exercise, acts systemically and influences energy metabolism and immune responses [219,357,360].

KYNA acts as a signaling molecule that binds to the adipose tissue receptor GPR35 during exercise and triggers browning of white adipocytes, while reducing inflammation [361]. Kynurenine metabolites are exerkines that link exercise to both mental health and metabolism. Exercise in humans reduces resting KYN concentration while increasing KYNA concentration, indicating the activation of this beneficial pathway [357,360].

Cancer-associated inflammation leads to elevated KYN levels and unbalanced QUINA/KYNA ratios. Overexpression of IDO1 in tumor cells and stromal immune cells results in tryptophan depletion and KYN accumulation, which suppress antitumor T-cell responses and promote regulatory T-cell differentiation [358,359]. The chronic activation of KP in cachexia leads to muscle wasting through multiple pathways, including activation of KYN-aryl hydrocarbon receptor (AhR) pathways that induce muscle-specific E3 ubiquitin ligases (e.g., MAFbx/atrogin-1), which cause proteasomal degradation. Cancer-associated inflammation leads to elevated KYN levels and unbalanced QUINA/KYNA ratios. Overexpression of IDO1 in tumor cells and stromal immune cells causes tryptophan depletion and KYN accumulation, which suppresses antitumor T-cell responses and promotes regulatory T-cell differentiation. Chronic activation of KP in cachexia leads to muscle wasting through multiple pathways, including activation of the aryl hydrocarbon receptor (AhR) pathways that induce muscle-specific E3 ubiquitin ligases (e.g., MAFbx/atrogin-1) that cause proteasomal degradation [358,359]. Elevated QUINA levels can damage the neuromuscular junction and consequently impair neurotransmission and muscle contractility. Furthermore, KP metabolites block AMPK activity and reduce mitochondrial formation and energy production in skeletal muscle [223,358,359].

In vitro, KYNA stimulates myoblast differentiation at low doses via AMPK activation, but at higher doses, it inhibits mTORC1, which impairs protein synthesis. Exercise-induced KYNA can enhance muscle repair at physiological concentrations and contribute to muscle atrophy at pathological level [223,358,359].

#### 4.2.5. 12,13-diHOME

In addition to water-soluble metabolites, some lipid molecules also function as cytokines. A good example is 12,13-diHOME, a fatty acid derivative (dihydroxy-linoleic acid) that increases blood concentrations after exercise [219,362]. BAT produces this molecule after cold exposure and exercise, and appears to act on muscles to enhance the uptake of fatty acids as fuel [362,363]. In effect, 12,13-diHOME signals that the body prefers fat as an energy source post-exercise [362,364]. It is one of several lipid mediators that increases with exercise and may improve metabolic flexibility. In humans, a single bout of moderate exercise produced a marked increase in plasma 12,13-diHOME levels across age and sex groups. Studies in mice confirmed that BAT is the primary source of this surge. For instance, surgical removal of BAT abolishes the exercise-induced 12,13-diHOME increase [362]. The discovery of exercise-regulated lipids such as 12,13-diHOME underscores that not only proteins and classical metabolites, but also bioactive lipids participate in organ-to-organ communication during exercise [219,362,364]. Thus, 12,13-diHOME is recognized as an endocrine mediator that links activated BAT to systemic metabolic effects during cold stress or physical activity.

Direct studies specifically linking 12,13-diHOME to cancer cachexia are still emerging; however, current evidence allows us to outline a plausible role. Given that 12,13-diHOME is a well-established marker and mediator of BAT activation, it is reasonable to expect that the cachexia-associated browning of fat would elevate 12,13-diHOME levels. For example, in cachectic mice, tumors induce a BAT-like phenotype in adipose depots (high UCP1, increased thermogenesis), which is analogous to cold exposure or exercise in healthy organisms, known to spike 12,13-diHOME [130,365]. Indeed, enhanced β-adrenergic stimulation of adipose tissue (such as the high catecholamine tone in cachexia) could drive the release of lipokines, such as 12,13-diHOME. Thus, 12,13-diHOME likely participates in the cachectic milieu as a signal for hyperactive fat metabolism [130,365]. Given its diverse biological actions, 12,13-diHOME may have both beneficial and adverse effects in cancer cachexia.

### 4.3. Extracellular Vesicles and RNA-Based Exerkines

One of the exciting frontiers in exerkine research is the role of extracellular vesicles (EVs), tiny membrane-bound particles such as exosomes and microvesicles, in signal transduction [220]. When muscles contract during exercise, they not only secrete free molecules but also shed an increased number of EVs into the bloodstream (Table 4). These vesicles (typically 30–150 nm in the case of exosomes) carry cargo like proteins, microRNAs, messenger RNAs, and even mitochondrial DNA. EVs protect their cargo from degradation and can deliver it specifically to target cells [220].

Evidence has shown that an acute bout of endurance exercise can significantly boost the circulating exosome levels. The contents of these exercise-induced vesicles suggest that they are a delivery system for exerkines. Many known protein exerkines and cytokines have been identified in exosomes [220]. The hypothesis is that packaging signals in EVs allow exercise-stressed cells to influence distant tissues in a precise manner, contributing to the multisystem benefits of exercise. For example, exosomes released by exercising muscles may fuse with adipocytes and release microRNAs that alter fat cell gene expression, promoting fat breakdown. This indirect mode of communication adds a layer of regulation to the freely circulating factors [220].

MicroRNAs (miRNAs) are the major components of EVs in response to exercise [219,220]. miRNAs act as small non-coding RNA molecules that are approximately twenty-two nucleotides in length and regulate cellular gene expression. The release of individual miRNAs encapsulated in exosomes from tissues into the circulation occurs during and after exercise. These circulating miRNAs function as endocrine signals that modify protein expression in the target organs. Several studies have demonstrated the existence of dozens of exercise-responsive miRNAs [219,220].

Studies on young adults who underwent 12 months of aerobic rowing training showed increased levels of a specific exosomal miRNA, miR-342-5p, which is produced by the endothelium. miRNAs exert cardioprotective effects by targeting pro-apoptotic proteins [366]. A study in mice highlighted miR-1192 as a novel exerkine, and after four weeks of swimming exercise, muscle and cardiac tissue in mice had increased levels of miR-1192, which indicates the protection of heart cells from hypoxia-induced death by suppressing caspase-3 (an apoptosis enzyme) [249]. Injecting mice with an miR-1192 mimic reproduced the exercise’s cardiac protection, while blocking miR-1192 reversed this benefit [249]. These findings provide convincing evidence that miR-1192 is an exerkine messenger during exercise that directly mediates a healthy effect (in this case, cardioprotection). Interestingly, miR-1192 has also been found in muscle fibers, where it can moderate muscle cell differentiation; therefore, its beneficial effect seems to be specific to the heart [249].

Other miRNAs have also been implicated in exercise adaptation. For example, miR-206 and miR-133 (muscle-specific miRNAs) participate in muscle growth and regeneration and their levels change with training. miR-146a and miR-21 are induced by exercise and have anti-inflammatory roles [219,367]. Circulating miR-126 (an endothelial miRNA) levels may increase after exercise, potentially supporting vascular repair. Extensive profiling has shown that acute exercise transiently increases EV-bound miRNAs such as miR-10b-5p, miR-222-3p, and miR-30a-5p, which appear to originate from diverse cell types (endothelial cells, immune cells, etc.) and may influence angiogenesis and immune function [219,367].

Recent studies have shown that EVs are important carriers of cachexia-induced signals. Tumors and other cells in the microenvironment release EVs carrying bioactive cargo (proteins, lipids, and nucleic acids), which can travel systemically to distant tissues [368]. Cancer cell-derived EVs have been shown to induce significant catabolic changes in skeletal muscles by disrupting the balance between protein synthesis and degradation. EVs from cachexia-inducing tumors deliver specific miRNAs and other factors into muscle fibers and reprogram gene expression to promote atrophy. Tumor-derived EVs can also directly reprogram adipose tissue metabolism, promoting lipolysis (breakdown of stored fat) and browning of WAT [368,369]. EVs are also implicated in broader cachectic phenomena such as chronic inflammation and anorexia. Tumor EVs can propagate systemic inflammation (elevated CRP, IL-6, etc.) by ferrying inflammatory mediators that feed into the muscle and fat catabolism. EVs may even influence the central nervous system; cachexia-related tumors often release factors that act on the hypothalamus to suppress appetite (e.g., GDF15, IL-1β) [368,370].

The benefits of EVs as potential therapeutic agents for CC are currently being investigated. The treatment approach involves the use of EVs extracted from exercised muscle tissue or engineered vesicles containing specific anti-atrophy RNAs. In a proof-of-concept, Di Felice et al. [371] developed a “physiactisome,” a nanoscale vesicle carrying the chaperone protein HSP60 (which is upregulated by exercise), and showed it could reduce tumor-induced muscle wasting in preclinical models.

Developing engineered vesicles also has the therapeutic potential to increase anti-inflammatory and pro-anabolic signals in patients with cachexia. For example, a pioneering 2023 study revealed the incorporation of small interfering RNA (siRNA) therapeutics into red blood cell–derived EVs for muscle-targeted delivery in the treatment of cachexia [372]. Repeated administration of RBC-EVs containing siRNA against myostatin resulted in >80% myostatin reduction in skeletal muscle, which produced larger muscle fibers and blocked cachexia development in tumor-bearing mice [372]. The ability of EVs to penetrate biological barriers while targeting specific cells renders them suitable for precise therapeutic delivery.

## 5. Conclusions

Despite a large amount of research on this topic, there is still controversy regarding the relationship between physical exercise and cancer cachexia and the role of exerkines in this process [180]. One of the most important debates is the safety and effectiveness of exercise in patients who already suffer from cachexia [180]. Exercise benefits most cancer patients; however, its impact on patients with cachexia remains uncertain. Some historical perspectives and recent commentaries have cautioned against exercise in patients with cachexia. Exercise may have adverse effects on patients with cachexia because their limited energy capacity may not support additional energy requirements [180]. However, recent clinical trials indicate that structured exercise supervised by a healthcare professional may be safe and effective for this population, improving physical function and muscle mass [10,17,373,374].

However, robust clinical evidence remains limited. Despite growing interest, very few clinical studies have evaluated exercise interventions in patients who fulfil the criteria for cancer cachexia. A 2021 Cochrane review [16] found that no randomized controlled trials (RCTs) included participants who met the international consensus criteria for cachexia. Population studies conducted on patients with advanced cancer or partial cachexia do not provide sufficient evidence for their direct application in patients with confirmed cachexia [16]. Much of our knowledge about the mechanisms and potential benefits of exercise in this area comes from preclinical animal models in which exercise is performed before the onset of cachexia [175,375,376]. A significant gap exists between preclinical findings and the development of safe and effective treatments in humans, partly because of the complexity of the disease and the heterogeneity of patients [17].

The mechanism by which exercise reverses the process of muscle wasting in cancer patients is not fully understood [181,187]. Although exercise is known to affect processes such as protein turnover, inflammation, and mitochondrial function, a deeper understanding of how these mechanisms are influenced by exercise in the context of cachexia is required [181,187]. There is a lack of clarity and consensus regarding the most appropriate exercise regimen (mode, type, dosage, and timing) for patients with cancer cachexia [15,17,168]. Different exercise regimens and types may produce different effects, and inappropriate exercise choices may be harmful. Individualized exercise prescriptions tailored to patient limitations and specific outcomes are recommended; however, further research is warranted [168].

Exercise induces the release of various signaling molecules known as exerkines. These factors are hypothesized to mediate numerous beneficial effects of exercise and hold potential as biomarkers for assessing the severity of cachexia and response to treatment [18]. However, the current understanding of the physiological mechanisms involved and the importance of studying exerkines, particularly in the context of cancer cachexia, is regarded as fundamental. There is a significant knowledge gap regarding exerkines in patients with cancer [18].

Future research should focus on investigating the role of exerkines, their impact on different organs, and immunological and physiological mechanisms involved in cachexia. Exerkines offer a promising direction for future studies, and may play a role in the development of tailored exercise regimens [18]. While many exerkines have beneficial effects on healthy individuals or at physiological exercise-induced concentrations, the same substances may have adverse effects when chronically elevated or produced in excessive amounts by cancerous tissue. This highlights the critical importance of the dose, duration, and specific pathological context [18,180,377]. Another controversy pertains to the role of BAT-derived factors: Do they actually mitigate some aspects of muscle wasting leading to cachexia, or do they exacerbate it? [133,135,136,137,138].

## Figures and Tables

**Table 1:** Key Characteristics of Cancer Cachexia.

Characteristic	Description	Underlying Mechanism
Weight Loss	Unintentional and progressive body weight loss.	Negative energy balance; increased resting energy expenditure; tumor metabolism. Not reversed by nutritional support alone.
Muscle Wasting (Skeletal & Cardiac)	Significant, involuntary loss of skeletal muscle mass; affects locomotion, respiratory muscles, and myocardium.	Cytokine excess; altered protein metabolism (proteolysis, reduced synthesis); ubiquitin-proteasome system (UPS) activation; calpain activation. Not reversed by nutritional support alone.
Fat Loss	Often accompanies muscle loss, but can occur independently.	Increased lipolysis; adipose tissue browning; tumor-secreted microRNAs. Not reversed by nutritional support alone
Anorexia	Lack of hunger, disinterest in eating; distinct from anorexia nervosa.	Inflammatory mediators (cytokines) inducing satiety; digestive factors (nausea, dysgeusia). Limited improvement with nutritional interventions alone.
Fatigue & Weakness	Extreme exhaustion, poor tolerance to activity; impacts daily tasks and self-care.	Muscle loss; altered energy metabolism; increased symptom burden (pain, sleep disturbances). A multimodal approach is needed.
Systemic Inflammation	Chronic, widespread inflammation throughout the body.	Host cytokines (TNF-α, IL-6, IL-1β); tumor-derived factors; acute phase response. Central driver, often self-perpetuating.
Insulin Resistance	Muscles and fat cells do not respond properly to insulin.	Tumor-secreted factors (e.g., ImpL2); impaired anabolic response. Contributes to muscle loss.
Increased Protein Turnover	Proteins break down too rapidly to be replaced.	Catabolic state; reduced anabolic hormones (IGF-1, testosterone, ghrelin). Leads to net protein loss
Quality of Life Reduction	Increased severity of pain, dry mouth, vomiting, dysgeusia, early satiety, sleep disturbances, anxiety.	Significantly impaired. Direct and indirect effects of the syndrome.
Reduced Therapy Tolerance	Patients too weak for effective chemotherapies and radiotherapies.	Overall debilitation and poor clinical parameters. Major clinical dilemma.
Increased Mortality	Accounts for 20–25% of all cancer-related deaths; powerful predictor of poor survival.	Impaired vital organ function (heart, respiratory muscles); systemic decline.

**Table 2:** Protein and Peptide Exerkines relevant to cancer cachexia.

Exerkine	Primary Source(s)	Exercise Response (Acute/Chronic)	Cachexia-Relevant Findings	Mechanisms/Pathways	Context-Dependent Notes
IL-6	Skeletal muscle (myokine); also immune cells	Acute ↑ markedly; chronic training lowers basal IL-6	Chronic tumor-driven IL-6/trans-signaling promotes wasting; exercise-induced IL-6 supports metabolism	JAK/STAT3; classical vs. trans-signaling; immune cell redistribution	Beneficial when transient post-exercise; harmful when persistently elevated from tumors/systemic inflammation
IL-8	Skeletal muscle; immune cells	Acute ↑	Indirect; pro-angiogenic/immune roles; limited direct cachexia data	Chemokine signaling	General inflammation vs. reparative angiogenesis may diverge by context
IL-10	Immune cells; possibly muscle-modulated	Acute ↑ with exercise-induced anti-inflammatory milieu	Anti-inflammatory; limited direct cachexia data	Cytokine anti-inflammatory signaling	Anti-inflammatory post-exercise; systemic deficiency/excess may alter outcomes
IL-15	Skeletal muscle (myokine)	Acute ↑; training effects vary	Lower serum in cachectic cancer reported in some cohorts; IL-15 antagonizes tumor-induced muscle protein loss in rats	Anabolic/anti-atrophy; glucose handling	Local muscle effects beneficial; systemic disease may blunt signaling
IGF-1 (hepatic + muscle isoforms)	Liver (endocrine); skeletal muscle isoforms (autocrine/paracrine)	Acute ↑ (circulating) variably; muscle IGF-1Ea/Ec upregulated post-exercise	IGF-1 suppressed in experimental cachexia; IGF-1 treatment attenuates lean mass loss in rats	PI3K/Akt/mTOR; inhibits FoxO-E3 ligases; supports satellite cells	Exercise restores local IGF-1 pools; systemic deficiency in cachexia limits anabolism
Myostatin (GDF8)	Skeletal muscle; also tumors	Decreases with resistance/endurance training	Often elevated in cachexia; tumor-derived myostatin correlates with muscle loss and worse survival	ActRIIB/Smad2/3; inhibits protein synthesis, promotes proteolysis	Exercise-lowered myostatin is beneficial; tumor or chronic elevation is catabolic
Follistatin	Liver and skeletal muscle (hepatokine/myokine)	Plasma ↑ after exercise; training ↑	Reduced in cachexia; neutralizes myostatin/activin A; FST mRNA therapy preserves muscle and survival in models	Binds myostatin/activin; activates Akt-mTOR	Generally protective; deficit favors wasting
Decorin	Skeletal muscle (ECM proteoglycan/myokine)	↑ after resistance, HIIT; released from contracting myotubes	Binds myostatin; anti-inflammatory; onco-suppressive; Ad.dcn mitigates cachexia in mice	Sequesters TGF-β/myostatin; modulates RTKs; reduces E3 ligases	Exercise-induced decorin supports hypertrophy; deficits may permit atrophy
Irisin (FNDC5)	Skeletal muscle; adipose expression changes	↑ with acute/chronic exercise	Conflicting in cancer: higher with cachexia in some data; lower in specific cancers; increases bone mass in mice	Browning; αV-integrin signaling in bone; metabolic modulation	Exercise-driven pulses beneficial; tumor/systemic disease may dysregulate levels
Fibcd1	Skeletal muscle (cleaved ectodomain)	Exercise-regulated myokine (preclinical)	Recombinant Fibcd1 reduces cancer-induced myofiber atrophy in mice	Myofiber size regulation (receptor engagement on muscle)	Therapeutic replacement shows benefit without stimulating tumor growth (preclinical)
Apelin	Skeletal muscle; endothelium; multiple organs	↑ with exercise (muscle/endothelium)	Higher serum in gastroesophageal cancer (esp. cachectic) without severity correlation	APJ signaling; improves muscle metabolism	Generally beneficial post-exercise; tumor/systemic elevations may be maladaptive
FGF21	Liver, adipose, muscle	Acute ↑; chronic training may ↓ in metabolic disease	Elevated in cachectic patients; muscle overexpression induces autophagy and muscle loss in mice	FGFR1/β-Klotho; mitochondrial integrity; adipose receptor upregulation with exercise	Physiological pulses adaptive; chronic elevation linked to atrophy
GDF15	Stress-responsive, multiple tissues	Acute intense exercise ↑ transiently	Elevated in cancer cachexia; suppresses appetite and directly induces muscle wasting; neutralization reverses weight loss in models	MAP3K11; Bcl-2/caspase-3; brain appetite centers	Transient exercise spikes vs. chronic tumor-driven elevation (harmful)
Osteocalcin (OC)	Bone (osteoblasts)	↑ after acute and chronic exercise	No direct data in cancer cachexia; supports muscle function; osteocalcin-IL-6 axis during exercise	UcOC endocrine effects; bone-muscle crosstalk	Beneficial exercise hormone; cachexia role unknown
BDNF	Skeletal muscle and brain	↑ with exercise (esp. resistance)	No direct cachexia data; supports muscle regeneration and metabolism	Satellite cell activation; AMPK–PGC1α	Likely beneficial with training; deficits may impair repair
Cathepsin B	Skeletal muscle	↑ with exercise; crosses BBB (preclinical)	Neurogenic benefits; cachexia link indirect	Neurogenesis support	Physiological signals beneficial; chronic disease context uncertain
Natriuretic peptides (ANP/BNP)	Heart (cardiokines)	↑ with cardiac load	Mobilize fat; unclear direct cachexia effects	Lipolysis; BAT activation	Exercise cardiometabolic benefits; disease elevations may reflect cardiac stress
ANGPTL4	Liver (systemic during exercise), muscle (local)	Acute ↑ (hepatic dominates systemic)	Chronically elevated in tumors; associates with inflammation, fat wasting; neutralization beneficial in models	Lipid trafficking; endothelial permeability; inflammatory signaling	Short-lived exercise elevations coordinate fuel use; chronic tumor secretion is catabolic
Adiponectin (adipokine)	Adipose tissue	Chronic training ↑; context dependent	AdipoRon (AdipoR agonist) and rosiglitazone-mediated restoration ameliorate cachexia in mice	Anti-inflammatory; metabolic remodeling	Exercise-raised adiponectin generally protective; tumor context varies
Leukemia Inhibitory Factor (LIF)	Skeletal muscle, immune cells, tumor cells	↑ in some cancers; exercise regulation unclear but animal data suggest muscle LIF release during intense contraction	Elevated in certain tumor types contributes to muscle wasting; in muscle, acute release may promote satellite cell activation	JAK/STAT3 signaling; inflammation and muscle regeneration pathways	Beneficial acute myokine role in regeneration; chronic tumor-driven secretion linked to cachexia

Ad.dcn—adenovirus-mediated decorin gene delivery; AMPK—AMP-activated protein kinase; ANGPTL4—angiopoietin-like 4; ANP/BNP—atrial natriuretic peptide/B-type natriuretic peptide; APJ—apelin receptor; BDNF—brain-derived neurotrophic factor; BAT—brown adipose tissue; dcn—decorin gene; ECM—extracellular matrix; FST—follistatin; FNDC5—fibronectin type III domain-containing protein 5 (precursor of irisin); FGF21—fibroblast growth factor 21; FGFR1—fibroblast growth factor receptor 1; FoxO—forkhead box O transcription factors; GDF15—growth differentiation factor 15; GDF8—growth differentiation factor 8 (myostatin); IGF-1Ea/Ec—insulin-like growth factor 1 isoforms Ea and Ec; IL—interleukin; JAK/STAT—Janus kinase/signal transducer and activator of transcription; LIF—leukemia inhibitory factor; MAP3K11—mitogen-activated protein kinase kinase kinase 11; mTOR—mechanistic target of rapamycin; OC—osteocalcin; PI3K—phosphoinositide 3-kinase; RTKs—receptor tyrosine. ↑ indicates an increase in circulating levels or expression of the given exerkine in response to exercise

**Table 3:** Metabolic Exerkines.

Exerkine	Primary Source(s)	Exercise Response (Acute/Chronic)	Cachexia-Relevant Findings	Mechanisms/Pathways	Context-Dependent Notes
Lactate (“lactomone”)	Working muscle	Acute ↑ with intensity	Signals metabolism; affects adipose lipolysis and brain appetite centers; direct cachexia data limited	Receptor-mediated signaling; Cori cycle; metabolic reprogramming	Physiological spikes beneficial; chronic tumor lactate may be maladaptive
Succinate (TCA dicarboxylate)	Muscle metabolism	Acute ↑; myometabokine	Regulates myokine secretion; direct cachexia data limited	Receptor (SUCNR1) signaling; paracrine crosstalk	Adaptive during exercise; disease-state accumulation may differ
BAIBA (β-aminoisobutyric acid)	Muscle (via PGC-1α)	↑ with training	Protects bone cells from oxidative stress; metabolic benefits; cachexia data limited	Browning; oxidative stress defense	Likely beneficial in physiological ranges
12,13-diHOME (lipokine)	Brown/white adipose; muscle-adipose axis	Acute ↑ after exercise	Promotes fatty acid uptake/oxidation; direct cachexia evidence limited	Lipid transport/oxidation	Supports exercise fuel handling; role in cachexia unknown
Kynurenine pathway (Kyn → KYNA vs. QUIN)	Muscle PGC-1α1 drives KATs; systemic tryptophan metabolism	Exercise shifts Kyn → KYNA (less neurotoxic)	Modulates stress/inflammation; muscle-brain axis; cachexia link indirect	KAT enzymes; PGC-1α1 program	Exercise-induced KYNA shift likely protective; chronic inflammation favors QUIN

BAIBA—β-aminoisobutyric acid; KYNA—kynurenic acid; Kyn—kynurenine; QUIN—quinolinic acid; PGC-1α—peroxisome proliferator-activated receptor gamma coactivator 1-alpha; SUCNR1—succinate receptor 1; ↑ indicates an increase in circulating levels or expression of the given exerkine in response to exercise; → indicates metabolic conversion from one compound to another.

**Table 4:** Extracellular Vesicles and RNA-Based Exerkines.

EV/RNA Exerkine	Source Tissue/Cell	Exercise Response	Proposed Targets/Actions	Cachexia-Relevant Findings	Context-Dependent Notes
Endothelial EV miR-342-5p	Endothelium	↑ with exercise	Cardiovascular protection; vascular adaptation	Anti-inflammatory/vascular benefits; indirect for cachexia	Physiological increases protective; tumor EV milieu may oppose
miR-1192 (exercise-induced)	Circulating (mouse)	↑ after exercise	Cardioprotection	Indirect; systemic resilience	Exercise protective; disease context unknown
miR-126 (endothelial)	Endothelium	↑ with exercise	Vascular homeostasis; angiogenesis	Indirect; may support perfusion	Exercise protective; tumor EVs may disrupt
Muscle-enriched miRs (miR-206, miR-133)	Skeletal muscle	Dynamic changes post-exercise	Myogenesis, regeneration	Potential to counteract atrophy; direct data limited	Training likely beneficial; chronic illness may blunt response
Inflammation-responsive miRs (miR-146a, miR-221, miR-21, miR-10b-5p, miR-222-3p, miR-30a-5p)	Circulating/various	Acute decreases/increases depending on miR and timing	Immune modulation; endothelial function; remodeling	Reflect inflammatory tone; potential biomarkers	Patterns differ with intensity/timing; tumor EV cargo may be opposite
HSP60 (exercise-upregulated; EV cargo candidate)	Skeletal muscle	↑ with endurance exercise	Mitochondrial stress signaling; therapy prototype (physiactisome)	Conceptually cytoprotective; therapeutic EV engineered from exercise factor	Endogenous signals beneficial; pharmacologic delivery under study

—blood–brain barrier; EV—extracellular vesicle; HSP—heat shock protein; miR—microRNA; ↑ indicates an increase in circulating levels or expression of the given exerkine in response to exercise.

## References

[1] Neshan M., Tsilimigras D.I., Han X., Zhu H., Pawlik T.M. (2024). Molecular Mechanisms of Cachexia: A Review. Cells.

[2] Poisson J., Martinez-Tapia C., Heitz D., Geiss R., Albrand G., Falandry C., Gisselbrecht M., Couderc A.-L., Boulahssass R., Liuu E. (2021). Prevalence and prognostic impact of cachexia among older patients with cancer: A nationwide cross-sectional survey (NutriAgeCancer). J. Cachexia Sarcopenia Muscle.

[3] Setiawan T., Sari I.N., Wijaya Y.T., Julianto N.M., Muhammad J.A., Lee H., Chae J.H., Kwon H.Y. (2023). Cancer cachexia: Molecular mechanisms and treatment strategies. J. Hematol. Oncol..

[4] Rupert J., Bonetto A., Narasimhan A., Liu Y., O’Connell T., Koniaris L., Zimmers T. (2020). IL-6 Trans-Signaling and Crosstalk Among Tumor, Muscle and Fat Mediate Pancreatic Cancer Cachexia. bioRxiv.

[5] Ni J., Zhang L. (2020). Cancer Cachexia: Definition, Staging, and Emerging Treatments. Cancer Manag. Res..

[6] Dizdar Ö., Kılıçkap S., Yalcin S., Philip P.A. (2019). Global Epidemiology of Gastrointestinal Cancers. Textbook of Gastrointestinal Oncology.

[7] Mamun T.I., Younus S., Rahman M.H. (2024). Gastric cancer-Epidemiology, modifiable and non-modifiable risk factors, challenges and opportunities: An updated review. Cancer Treat. Res. Commun..

[8] Cheung C., Boocock E., Grande A.J., Maddocks M. (2023). Exercise-based interventions for cancer cachexia: A systematic review of randomised and non-randomised controlled trials. Asia Pac. J. Oncol. Nurs..

[9] Bowers M., Petrasso C., McLuskie A., Bayly J., Laird B.J.A., Higginson I.J., Maddocks M. (2025). Multicomponent Interventions for Adults With Cancer Cachexia: A Systematic Review. J. Cachexia Sarcopenia Muscle.

[10] Clemente-Suarez V.J., Redondo-Florez L., Rubio-Zarapuz A., Martinez-Guardado I., Navarro-Jimenez E., Tornero-Aguilera J.F. (2022). Nutritional and Exercise Interventions in Cancer-Related Cachexia: An Extensive Narrative Review. Int. J. Environ. Res. Public Health.

[11] Bertocchi E., Frigo F., Buonaccorso L., Venturelli F., Bassi M.C., Tanzi S. (2024). Cancer cachexia: A scoping review on non-pharmacological interventions. Asia Pac. J. Oncol. Nurs..

[12] Constantina C., Mary E., George O., Konstantinos F., Christiana K., Nicos M., Andreas C. (2025). Nonpharmacological Management of Cancer-Related Cachexia: A Systematic Review. Semin. Oncol. Nurs..

[13] Maddocks M., Murton A.J., Wilcock A. (2012). Therapeutic exercise in cancer cachexia. Crit. Rev. Oncog..

[14] Horawski J.L., Fleszar-Pavlovic S.E., Lopez-Pentecost M., Crane T.E., Wheeler M.G., Kholodovsky E., Best T.M. (2025). The role of resistance training in mitigating cancer-induced cachexia: A systematic review. Sports Med. Health Sci..

[15] Tsitkanou S., Murach K.A., Washington T.A., Greene N.P. (2022). Exercise Counteracts the Deleterious Effects of Cancer Cachexia. Cancers.

[16] Grande A.J., Silva V., Sawaris Neto L., Teixeira Basmage J.P., Peccin M.S., Maddocks M. (2021). Exercise for cancer cachexia in adults. Cochrane Database Syst. Rev..

[17] Mavropalias G., Sim M., Taaffe D.R., Galvao D.A., Spry N., Kraemer W.J., Hakkinen K., Newton R.U. (2022). Exercise medicine for cancer cachexia: Targeted exercise to counteract mechanisms and treatment side effects. J. Cancer Res. Clin. Oncol..

[18] Ahmadi Hekmatikar A., Nelson A., Petersen A. (2023). Highlighting the idea of exerkines in the management of cancer patients with cachexia: Novel insights and a critical review. BMC Cancer.

[19] Fearon K., Strasser F., Anker S.D., Bosaeus I., Bruera E., Fainsinger R.L., Jatoi A., Loprinzi C., MacDonald N., Mantovani G. (2011). Definition and classification of cancer cachexia: An international consensus. Lancet Oncol..

[20] Brown L.R., Laird B.J.A., Wigmore S.J., Skipworth R.J.E. (2022). Understanding Cancer Cachexia and Its Implications in Upper Gastrointestinal Cancers. Curr. Treat. Options Oncol..

[21] Akezaki Y., Kikuuchi M., Hamada K., Ookura M. (2020). Incidence of cachexia in patients with advanced gastrointestinal cancer at the beginning of rehabilitation intervention. J. Phys. Ther. Sci..

[22] Valaire R., Garden F., Razmovski-Naumovski V. (2024). Are measures and related symptoms of cachexia recorded as outcomes in gastrointestinal cancer chemotherapy clinical trials?. J. Cachexia Sarcopenia Muscle.

[23] Tao Z., Chen Z., Gao Y., Quan M. (2024). Influence of cachexia on immunotherapy efficacy and prognosis for malignant tumors of the digestive system. Cancer Rep..

[24] Yule M.S., Brown L.R., Waller R., Wigmore S.J. (2024). Cancer cachexia. BMJ.

[25] Anandavadivelan P., Lagergren P. (2016). Cachexia in patients with oesophageal cancer. Nat. Rev. Clin. Oncol..

[26] Guo Z.X., Ma J.L., Zhang J.Q., Yan L.L., Zhou Y., Mao X.L., Li S.W., Zhou X.B. (2025). Metabolic reprogramming and immunological changes in the microenvironment of esophageal cancer: Future directions and prospects. Front. Immunol..

[27] Li L., Ling Z.Q. (2024). Mechanisms of cancer cachexia and targeted therapeutic strategies. Biochim. Biophys. Acta Rev. Cancer.

[28] Baltgalvis K.A., Berger F.G., Peña M.M.O., Davis J.M., White J.P., Carson J.A. (2009). Muscle wasting and interleukin-6-induced atrogin-I expression in the cachectic Apc Min/+ mouse. Pflügers Arch.—Eur. J. Physiol..

[29] Patel H.J., Patel B.M. (2017). TNF-α and cancer cachexia: Molecular insights and clinical implications. Life Sci..

[30] Tisdale M.J. (2010). Cancer cachexia. Curr. Opin. Gastroenterol..

[31] Tisdale M.J. (2005). The ubiquitin-proteasome pathway as a therapeutic target for muscle wasting. J. Support. Oncol..

[32] Stovroff M.C., Fraker D.L., Swedenborg J.A., Norton J.A. (1988). Cachectin/tumor necrosis factor: A possible mediator of cancer anorexia in the rat. Cancer Res..

[33] Chen J.L., Colgan T.D., Walton K.L., Gregorevic P., Harrison C.A. (2016). The TGF-β signalling network in muscle development, adaptation and disease. Growth Factors and Cytokines in Skeletal Muscle Development, Growth, Regeneration and Disease.

[34] Argilés J.M., Busquets S., Stemmler B., López-Soriano F.J. (2014). Cancer cachexia: Understanding the molecular basis. Nat. Rev. Cancer.

[35] Murphy K.T., Chee A., Gleeson B.G., Naim T., Swiderski K., Koopman R., Lynch G.S. (2011). Antibody-directed myostatin inhibition enhances muscle mass and function in tumor-bearing mice. Am. J. Physiol.—Regul. Integr. Comp. Physiol..

[36] Padrao A.I., Moreira-Goncalves D., Oliveira P.A., Teixeira C., Faustino-Rocha A.I., Helguero L., Vitorino R., Santos L.L., Amado F., Duarte J.A. (2015). Endurance training prevents TWEAK but not myostatin-mediated cardiac remodelling in cancer cachexia. Arch. Biochem. Biophys..

[37] Klimek M.E.B., Aydogdu T., Link M.J., Pons M., Koniaris L.G., Zimmers T.A. (2010). Acute inhibition of myostatin-family proteins preserves skeletal muscle in mouse models of cancer cachexia. Biochem. Biophys. Res. Commun..

[38] Loumaye A., de Barsy M., Nachit M., Lause P., Frateur L., van Maanen A., Trefois P., Gruson D., Thissen J.P. (2015). Role of Activin A and myostatin in human cancer cachexia. J. Clin. Endocrinol. Metab..

[39] Kim Y.-M., Sanborn M.A., Vijeth S., Gajwani P., Wang X., Jung D., Valyi-Nagy T., Chakraborty S., Mancinelli G., Toth P.T. (2025). Skeletal muscle endothelial dysfunction through the activin A–PGC1α axis drives progression of cancer cachexia. Nat. Cancer.

[40] Winkles J.A. (2008). The TWEAK-Fn14 cytokine-receptor axis: Discovery, biology and therapeutic targeting. Nat. Rev. Drug Discov..

[41] Tajrishi M.M., Zheng T.S., Burkly L.C., Kumar A. (2014). The TWEAK-Fn14 pathway: A potent regulator of skeletal muscle biology in health and disease. Cytokine Growth Factor. Rev..

[42] Marceca G.P., Londhe P., Calore F. (2020). Management of Cancer Cachexia: Attempting to Develop New Pharmacological Agents for New Effective Therapeutic Options. Front. Oncol..

[43] Tomaz da Silva M., Roy A., Vuong A.T., Joshi A.S., Josphien C., Trivedi M.V., Hindi S.M., Narkar V.A., Kumar A. (2025). The TWEAK/Fn14 signaling mediates skeletal muscle wasting during cancer cachexia. iScience.

[44] Johnston A.J., Murphy K.T., Jenkinson L., Laine D., Emmrich K., Faou P., Weston R., Jayatilleke K.M., Schloegel J., Talbo G. (2015). Targeting of Fn14 Prevents Cancer-Induced Cachexia and Prolongs Survival. Cell.

[45] Yu Y.C., Ahmed A., Lai H.C., Cheng W.C., Yang J.C., Chang W.C., Chen L.M., Shan Y.S., Ma W.L. (2022). Review of the endocrine organ-like tumor hypothesis of cancer cachexia in pancreatic ductal adenocarcinoma. Front. Oncol..

[46] Liu M., Ren Y., Zhou Z., Yang J., Shi X., Cai Y., Arreola A.X., Luo W., Fung K.-M., Xu C. (2024). The crosstalk between macrophages and cancer cells potentiates pancreatic cancer cachexia. Cancer Cell.

[47] Webster J.M., Kempen L., Hardy R.S., Langen R.C.J. (2020). Inflammation and Skeletal Muscle Wasting During Cachexia. Front. Physiol..

[48] Wang Y.F., An Z.Y., Lin D.H., Jin W.L. (2022). Targeting cancer cachexia: Molecular mechanisms and clinical study. MedComm.

[49] Park S.Y., Hwang B.O., Song N.Y. (2023). The role of myokines in cancer: Crosstalk between skeletal muscle and tumor. BMB Rep..

[50] Kwak K.S., Zhou X., Solomon V., Baracos V.E., Davis J., Bannon A.W., Boyle W.J., Lacey D.L., Han H.Q. (2004). Regulation of protein catabolism by muscle-specific and cytokine-inducible ubiquitin ligase E3alpha-II during cancer cachexia. Cancer Res..

[51] Aniort J., Stella A., Philipponnet C., Poyet A., Polge C., Claustre A., Combaret L., Béchet D., Attaix D., Boisgard S. (2019). Muscle wasting in patients with end-stage renal disease or early-stage lung cancer: Common mechanisms at work. J. Cachexia Sarcopenia Muscle.

[52] Fu T.M., Shen C., Li Q., Zhang P., Wu H. (2018). Mechanism of ubiquitin transfer promoted by TRAF6. Proc. Natl. Acad. Sci. USA.

[53] Paul P.K., Gupta S.K., Bhatnagar S., Panguluri S.K., Darnay B.G., Choi Y., Kumar A. (2010). Targeted ablation of TRAF6 inhibits skeletal muscle wasting in mice. J. Cell Biol..

[54] Bodine S.C., Baehr L.M. (2014). Skeletal muscle atrophy and the E3 ubiquitin ligases MuRF1 and MAFbx/atrogin-1. Am. J. Physiol. Endocrinol. Metab..

[55] Sun Y.S., Ye Z.Y., Qian Z.Y., Xu X.D., Hu J.F. (2012). Expression of TRAF6 and ubiquitin mRNA in skeletal muscle of gastric cancer patients. J. Exp. Clin. Cancer Res..

[56] Peris-Moreno D., Cussonneau L., Combaret L., Polge C., Taillandier D. (2021). Ubiquitin Ligases at the Heart of Skeletal Muscle Atrophy Control. Molecules.

[57] Segatto M., Fittipaldi R., Pin F., Sartori R., Dae Ko K., Zare H., Fenizia C., Zanchettin G., Pierobon E.S., Hatakeyama S. (2017). Epigenetic targeting of bromodomain protein BRD4 counteracts cancer cachexia and prolongs survival. Nat. Commun..

[58] VanderVeen B.N., Fix D.K., Carson J.A. (2017). Disrupted Skeletal Muscle Mitochondrial Dynamics, Mitophagy, and Biogenesis during Cancer Cachexia: A Role for Inflammation. Oxidative Med. Cell. Longev..

[59] Carson J.A., Hardee J.P., VanderVeen B.N. (2016). The emerging role of skeletal muscle oxidative metabolism as a biological target and cellular regulator of cancer-induced muscle wasting. Semin. Cell Dev. Biol..

[60] McLean J.B., Moylan J.S., Andrade F.H. (2014). Mitochondria dysfunction in lung cancer-induced muscle wasting in C2C12 myotubes. Front. Physiol..

[61] Hardee J.P., Montalvo R.N., Carson J.A. (2017). Linking Cancer Cachexia-Induced Anabolic Resistance to Skeletal Muscle Oxidative Metabolism. Oxidative Med. Cell. Longev..

[62] Laviano A., Seelaender M., Rianda S., Silverio R., Rossi Fanelli F. (2012). Neuroinflammation: A contributing factor to the pathogenesis of cancer cachexia. Crit. Rev. Oncog..

[63] Le Thuc O., Stobbe K., Cansell C., Nahon J.L., Blondeau N., Rovère C. (2017). Hypothalamic Inflammation and Energy Balance Disruptions: Spotlight on Chemokines. Front. Endocrinol..

[64] Vohra M.S., Benchoula K., Serpell C.J., Hwa W.E. (2022). AgRP/NPY and POMC neurons in the arcuate nucleus and their potential role in treatment of obesity. Eur. J. Pharmacol..

[65] Olson B., Diba P., Korzun T., Marks D.L. (2021). Neural Mechanisms of Cancer Cachexia. Cancers.

[66] Wang Y., Dong Z., An Z., Jin W. (2024). Cancer cachexia: Focus on cachexia factors and inter-organ communication. Chin. Med. J..

[67] Kanter N.G., Cohen-Woods S., Balfour D.A., Burt M.G., Waterman A.L., Koczwara B. (2024). Hypothalamic-Pituitary-Adrenal Axis Dysfunction in People With Cancer: A Systematic Review. Cancer Med..

[68] Jiao Z.T., Luo Q. (2022). Molecular Mechanisms and Health Benefits of Ghrelin: A Narrative Review. Nutrients.

[69] Thompson N.M., Gill D.A., Davies R., Loveridge N., Houston P.A., Robinson I.C., Wells T. (2004). Ghrelin and des-octanoyl ghrelin promote adipogenesis directly in vivo by a mechanism independent of the type 1a growth hormone secretagogue receptor. Endocrinology.

[70] Terawaki K., Kashiwase Y., Sawada Y., Hashimoto H., Yoshimura M., Ohbuchi K., Sudo Y., Suzuki M., Miyano K., Shiraishi S. (2017). Development of ghrelin resistance in a cancer cachexia rat model using human gastric cancer-derived 85As2 cells and the palliative effects of the Kampo medicine rikkunshito on the model. PLoS ONE.

[71] Chen J.A., Splenser A., Guillory B., Luo J., Mendiratta M., Belinova B., Halder T., Zhang G., Li Y.P., Garcia J.M. (2015). Ghrelin prevents tumour- and cisplatin-induced muscle wasting: Characterization of multiple mechanisms involved. J. Cachexia Sarcopenia Muscle.

[72] Porporato P.E., Filigheddu N., Reano S., Ferrara M., Angelino E., Gnocchi V.F., Prodam F., Ronchi G., Fagoonee S., Fornaro M. (2013). Acylated and unacylated ghrelin impair skeletal muscle atrophy in mice. J. Clin. Investig..

[73] Shao T., Verma H.K., Pande B., Costanzo V., Ye W., Cai Y., Bhaskar L. (2021). Physical Activity and Nutritional Influence on Immune Function: An Important Strategy to Improve Immunity and Health Status. Front. Physiol..

[74] Ouerghi N., Feki M., Bragazzi N.L., Knechtle B., Hill L., Nikolaidis P.T., Bouassida A. (2021). Ghrelin Response to Acute and Chronic Exercise: Insights and Implications from a Systematic Review of the Literature. Sports Med..

[75] Mani B.K., Castorena C.M., Osborne-Lawrence S., Vijayaraghavan P., Metzger N.P., Elmquist J.K., Zigman J.M. (2018). Ghrelin mediates exercise endurance and the feeding response post-exercise. Mol. Metab..

[76] Lovell A.J., Hoecht E.M., Hucik B., Cervone D.T., Dyck D.J. (2022). The effects of diet and chronic exercise on skeletal muscle ghrelin response. Metabol. Open.

[77] Fuoco D., Kilgour R.D., Vigano A. (2015). A hypothesis for a possible synergy between ghrelin and exercise in patients with cachexia: Biochemical and physiological bases. Med. Hypotheses.

[78] Neary N.M., Small C.J., Wren A.M., Lee J.L., Druce M.R., Palmieri C., Frost G.S., Ghatei M.A., Coombes R.C., Bloom S.R. (2004). Ghrelin increases energy intake in cancer patients with impaired appetite: Acute, randomized, placebo-controlled trial. J. Clin. Endocrinol. Metab..

[79] Hiura Y., Takiguchi S., Yamamoto K., Takahashi T., Kurokawa Y., Yamasaki M., Nakajima K., Miyata H., Fujiwara Y., Mori M. (2012). Effects of ghrelin administration during chemotherapy with advanced esophageal cancer patients: A prospective, randomized, placebo-controlled phase 2 study. Cancer.

[80] Yeom E., Yu K. (2022). Understanding the molecular basis of anorexia and tissue wasting in cancer cachexia. Exp. Mol. Med..

[81] Matsumoto T., Cho S., Nakasya A., Nagai H., Satake H., Yasui H. (2024). Early administration of anamorelin improves cancer cachexia in gastrointestinal cancer patients: An observational study. Sci. Rep..

[82] Hamauchi S., Furuse J., Takano T., Munemoto Y., Furuya K., Baba H., Takeuchi M., Choda Y., Higashiguchi T., Naito T. (2019). A multicenter, open-label, single-arm study of anamorelin (ONO-7643) in advanced gastrointestinal cancer patients with cancer cachexia. Cancer.

[83] Katakami N., Uchino J., Yokoyama T., Naito T., Kondo M., Yamada K., Kitajima H., Yoshimori K., Sato K., Saito H. (2018). Anamorelin (ONO-7643) for the treatment of patients with non–small cell lung cancer and cachexia: Results from a randomized, double-blind, placebo-controlled, multicenter study of Japanese patients (ONO-7643-04). Cancer.

[84] Naito T., Uchino J., Kojima T., Matano Y., Minato K., Tanaka K., Mizukami T., Atagi S., Higashiguchi T., Muro K. (2022). A multicenter, open-label, single-arm study of anamorelin (ONO-7643) in patients with cancer cachexia and low body mass index. Cancer.

[85] Garcia J.M., Friend J., Allen S. (2013). Therapeutic potential of anamorelin, a novel, oral ghrelin mimetic, in patients with cancer-related cachexia: A multicenter, randomized, double-blind, crossover, pilot study. Support. Care Cancer.

[86] Ge X., Yang H., Bednarek M.A., Galon-Tilleman H., Chen P., Chen M., Lichtman J.S., Wang Y., Dalmas O., Yin Y. (2018). LEAP2 Is an Endogenous Antagonist of the Ghrelin Receptor. Cell Metab..

[87] Lu X., Huang L., Huang Z., Feng D., Clark R.J., Chen C. (2021). LEAP-2: An Emerging Endogenous Ghrelin Receptor Antagonist in the Pathophysiology of Obesity. Front. Endocrinol..

[88] Varshney S., Shankar K., Kerr H.L., Anderson L.J., Gupta D., Metzger N.P., Singh O., Ogden S.B., Paul S., Piñon F. (2024). The LEAP2 Response to Cancer-Related Anorexia-Cachexia Syndrome in Male Mice and Patients. Endocrinology.

[89] Oneda E., Manno A., Noventa S., Libertini M., Cherri S., Zaniboni A. (2025). Role of diet, physical activity and new drugs in the primary management of cancer cachexia in gastrointestinal tumors—A comprehensive review. Front. Oncol..

[90] Sartori R., Hagg A., Zampieri S., Armani A., Winbanks C.E., Viana L.R., Haidar M., Watt K.I., Qian H., Pezzini C. (2021). Perturbed BMP signaling and denervation promote muscle wasting in cancer cachexia. Sci. Transl. Med..

[91] Daou N., Hassani M., Matos E., De Castro G.S., Galvao Figueredo Costa R., Seelaender M., Moresi V., Rocchi M., Adamo S., Li Z. (2020). Displaced Myonuclei in Cancer Cachexia Suggest Altered Innervation. Int. J. Mol. Sci..

[92] Boehm I., Miller J., Wishart T.M., Wigmore S.J., Skipworth R.J., Jones R.A., Gillingwater T.H. (2020). Neuromuscular junctions are stable in patients with cancer cachexia. J. Clin. Investig..

[93] Klein G.L., Petschow B.W., Shaw A.L., Weaver E. (2013). Gut barrier dysfunction and microbial translocation in cancer cachexia: A new therapeutic target. Curr. Opin. Support. Palliat. Care.

[94] Costa R.G.F., Caro P.L., de Matos-Neto E.M., Lima J., Radloff K., Alves M.J., Camargo R.G., Pessoa A.F.M., Simoes E., Gama P. (2019). Cancer cachexia induces morphological and inflammatory changes in the intestinal mucosa. J. Cachexia Sarcopenia Muscle.

[95] Genua F., Raghunathan V., Jenab M., Gallagher W.M., Hughes D.J. (2021). The Role of Gut Barrier Dysfunction and Microbiome Dysbiosis in Colorectal Cancer Development. Front. Oncol..

[96] Bindels L.B., Neyrinck A.M., Loumaye A., Catry E., Walgrave H., Cherbuy C., Leclercq S., Hul M.V., Plovier H., Pachikian B. (2018). Increased gut permeability in cancer cachexia: Mechanisms and clinical relevance. Oncotarget.

[97] Puppa M.J., White J.P., Sato S., Cairns M., Baynes J.W., Carson J.A. (2011). Gut barrier dysfunction in the Apc(Min/+) mouse model of colon cancer cachexia. Biochim. Biophys. Acta.

[98] Panebianco C., Villani A., Potenza A., Favaro E., Finocchiaro C., Perri F., Pazienza V. (2023). Targeting Gut Microbiota in Cancer Cachexia: Towards New Treatment Options. Int. J. Mol. Sci..

[99] Pin F., Prideaux M., Huot J.R., Essex A.L., Plotkin L.I., Bonetto A., Bonewald L.F. (2021). Non-bone metastatic cancers promote osteocyte-induced bone destruction. Cancer Lett..

[100] Kaji H. (2014). Interaction between Muscle and Bone. J. Bone Metab..

[101] Kaji H. (2025). Bone-muscle interactions. Osteoporos. Sarcopenia.

[102] Anastasilaki E., Paccou J., Gkastaris K., Anastasilakis A.D. (2023). Glucocorticoid-induced osteoporosis: An overview with focus on its prevention and management. Hormones.

[103] Zwickl H., Zwickl-Traxler E., Haushofer A., Seier J., Podar K., Weber M., Hackner K., Jacobi N., Pecherstorfer M., Vallet S. (2021). Effect of cachexia on bone turnover in cancer patients: A case-control study. BMC Cancer.

[104] Bonetto A., Kays J.K., Parker V.A., Matthews R.R., Barreto R., Puppa M.J., Kang K.S., Carson J.A., Guise T.A., Mohammad K.S. (2016). Differential Bone Loss in Mouse Models of Colon Cancer Cachexia. Front. Physiol..

[105] Kearns A.E., Khosla S., Kostenuik P.J. (2008). Receptor activator of nuclear factor kappaB ligand and osteoprotegerin regulation of bone remodeling in health and disease. Endocr. Rev..

[106] Pin F., Jones A.J., Huot J.R., Narasimhan A., Zimmers T.A., Bonewald L.F., Bonetto A. (2022). RANKL Blockade Reduces Cachexia and Bone Loss Induced by Non-Metastatic Ovarian Cancer in Mice. J. Bone Miner. Res..

[107] Sims N.A. (2021). Influences of the IL-6 cytokine family on bone structure and function. Cytokine.

[108] Anloague A., Delgado-Calle J. (2023). Osteocytes: New Kids on the Block for Cancer in Bone Therapy. Cancers.

[109] Riquelme M.A., Cardenas E.R., Jiang J.X. (2020). Osteocytes and Bone Metastasis. Front. Endocrinol..

[110] Wang T., Zhou D., Hong Z. (2025). Sarcopenia and cachexia: Molecular mechanisms and therapeutic interventions. MedComm.

[111] Herrmann M., Engelke K., Ebert R., Müller-Deubert S., Rudert M., Ziouti F., Jundt F., Felsenberg D., Jakob F. (2020). Interactions between Muscle and Bone-Where Physics Meets Biology. Biomolecules.

[112] Yuan S., Wan Z.-H., Cheng S.-L., Michaëlsson K., Larsson S.C. (2021). Insulin-like Growth Factor-1, Bone Mineral Density, and Fracture: A Mendelian Randomization Study. J. Clin. Endocrinol. Metab..

[113] Pin F., Bonewald L.F., Bonetto A. (2021). Role of myokines and osteokines in cancer cachexia. Exp. Biol. Med..

[114] Qin Y., Peng Y., Zhao W., Pan J., Ksiezak-Reding H., Cardozo C., Wu Y., Divieti Pajevic P., Bonewald L.F., Bauman W.A. (2017). Myostatin inhibits osteoblastic differentiation by suppressing osteocyte-derived exosomal microRNA-218: A novel mechanism in muscle-bone communication. J. Biol. Chem..

[115] Hamrick M.W., Shi X., Zhang W., Pennington C., Thakore H., Haque M., Kang B., Isales C.M., Fulzele S., Wenger K.H. (2007). Loss of myostatin (GDF8) function increases osteogenic differentiation of bone marrow-derived mesenchymal stem cells but the osteogenic effect is ablated with unloading. Bone.

[116] Galea G.L., Lanyon L.E., Price J.S. (2017). Sclerostin’s role in bone’s adaptive response to mechanical loading. Bone.

[117] Tsourdi E. (2022). RANKL blockade for cancer cachexia; A new therapeutic opportunity?. J. Bone Miner. Res..

[118] Pauk M., Saito H., Hesse E., Taipaleenmäki H. (2022). Muscle and Bone Defects in Metastatic Disease. Curr. Osteoporos. Rep..

[119] Booth F.W., Ruegsegger G.N., Olver T.D. (2016). Exercise Has a Bone to Pick with Skeletal Muscle. Cell Metab..

[120] Tamayo-Torres E., Garrido A., de Cabo R., Carretero J., Gomez-Cabrera M.C. (2024). Molecular mechanisms of cancer cachexia. Role of exercise training. Mol. Aspects Med..

[121] Daas S.I., Rizeq B.R., Nasrallah G.K. (2019). Adipose tissue dysfunction in cancer cachexia. J. Cell. Physiol..

[122] Mannelli M., Gamberi T., Magherini F., Fiaschi T. (2020). The Adipokines in Cancer Cachexia. Int. J. Mol. Sci..

[123] Batista Júnior M.L., Henriques F., Valarmathi M.T. (2018). Adipose Tissue Remodeling during Cancer Cachexia. Muscle Cells—Recent Advances and Future Perspectives.

[124] Batista M.L., Henriques F.S., Neves R.X., Olivan M.R., Matos-Neto E.M., Alcântara P.S., Maximiano L.F., Otoch J.P., Alves M.J., Seelaender M. (2016). Cachexia-associated adipose tissue morphological rearrangement in gastrointestinal cancer patients. J. Cachexia Sarcopenia Muscle.

[125] Geppert J., Rohm M. (2024). Cancer cachexia: Biomarkers and the influence of age. Mol. Oncol..

[126] Tsoli M., Moore M., Burg D., Painter A., Taylor R., Lockie S.H., Turner N., Warren A., Cooney G., Oldfield B. (2012). Activation of thermogenesis in brown adipose tissue and dysregulated lipid metabolism associated with cancer cachexia in mice. Cancer Res..

[127] Mota I.N.R., Satari S., Marques I.S., Santos J.M.O., Medeiros R. (2024). Adipose tissue rearrangement in cancer cachexia: The involvement of β3-adrenergic receptor associated pathways. Biochim. Biophys. Acta—Rev. Cancer.

[128] Kir S., White J.P., Kleiner S., Kazak L., Cohen P., Baracos V.E., Spiegelman B.M. (2014). Tumour-derived PTH-related protein triggers adipose tissue browning and cancer cachexia. Nature.

[129] Han J., Meng Q., Shen L., Wu G. (2018). Interleukin-6 induces fat loss in cancer cachexia by promoting white adipose tissue lipolysis and browning. Lipids Health Dis..

[130] Petruzzelli M., Schweiger M., Schreiber R., Campos-Olivas R., Tsoli M., Allen J., Swarbrick M., Rose-John S., Rincon M., Robertson G. (2014). A switch from white to brown fat increases energy expenditure in cancer-associated cachexia. Cell Metab..

[131] Rohm M., Schäfer M., Laurent V., Üstünel B.E., Niopek K., Algire C., Hautzinger O., Sijmonsma T.P., Zota A., Medrikova D. (2016). An AMP-activated protein kinase–stabilizing peptide ameliorates adipose tissue wasting in cancer cachexia in mice. Nat. Med..

[132] Xie H., Heier C., Meng X., Bakiri L., Pototschnig I., Tang Z., Schauer S., Baumgartner V.J., Grabner G.F., Schabbauer G. (2022). An immune-sympathetic neuron communication axis guides adipose tissue browning in cancer-associated cachexia. Proc. Natl. Acad. Sci. USA.

[133] Bos S.A., Gill C.M., Martinez-Salazar E.L., Torriani M., Bredella M.A. (2019). Preliminary investigation of brown adipose tissue assessed by PET/CT and cancer activity. Skelet. Radiol..

[134] Shellock F.G., Riedinger M.S., Fishbein M.C. (1986). Brown adipose tissue in cancer patients: Possible cause of cancer-induced cachexia. J. Cancer Res. Clin. Oncol..

[135] Becker A.S., Zellweger C., Bacanovic S., Franckenberg S., Nagel H.W., Frick L., Schawkat K., Eberhard M., Bluthgen C., Volbracht J. (2020). Brown fat does not cause cachexia in cancer patients: A large retrospective longitudinal FDG-PET/CT cohort study. PLoS ONE.

[136] Eljalby M., Huang X., Becher T., Wibmer A.G., Jiang C.S., Vaughan R., Schöder H., Cohen P. (2023). Brown adipose tissue is not associated with cachexia or increased mortality in a retrospective study of patients with cancer. Am. J. Physiol.—Endocrinol. Metab..

[137] Panagiotou G., Babazadeh D., Mazza D.F., Azghadi S., Cawood J.M., Rosenberg A.S., Imamura F., Forouhi N.G., Chaudhari A.J., Abdelhafez Y.G. (2025). Brown adipose tissue is associated with reduced weight loss and risk of cancer cachexia: A retrospective cohort study. Clin. Nutr..

[138] Chu K., Bos S.A., Gill C.M., Torriani M., Bredella M.A. (2020). Brown adipose tissue and cancer progression. Skelet. Radiol..

[139] Silvério R., Lira F.S., Oyama L.M., Oller do Nascimento C.M., Otoch J.P., Alcântara P.S.M., Batista M.L., Seelaender M. (2017). Lipases and lipid droplet-associated protein expression in subcutaneous white adipose tissue of cachectic patients with cancer. Lipids Health Dis..

[140] Takahashi M., Terashima M., Takagane A., Oyama K., Fujiwara H., Wakabayashi G. (2009). Ghrelin and leptin levels in cachectic patients with cancer of the digestive organs. Int. J. Clin. Oncol..

[141] Lee C.H., Woo Y.C., Wang Y., Yeung C.Y., Xu A., Lam K.S.L. (2015). Obesity, adipokines and cancer: An update. Clin. Endocrinol..

[142] Kerem M., Ferahkose Z., Yilmaz U.T., Pasaoglu H., Ofluoglu E., Bedirli A., Salman B., Sahin T.T., Akin M. (2008). Adipokines and ghrelin in gastric cancer cachexia. World J. Gastroenterol..

[143] Diakowska D., Krzystek-Korpacka M., Markocka-Maczka K., Diakowski W., Matusiewicz M., Grabowski K. (2010). Circulating leptin and inflammatory response in esophageal cancer, esophageal cancer-related cachexia–anorexia syndrome (CAS) and non-malignant CAS of the alimentary tract. Cytokine.

[144] Maurya R., Sebastian P., Namdeo M., Devender M., Gertler A. (2021). COVID-19 severity in obesity: Leptin and inflammatory cytokine interplay in the link between high morbidity and mortality. Front. Immunol..

[145] Naylor C., Petri W.A. (2016). Leptin regulation of immune responses. Trends Mol. Med..

[146] Sturgeon K., Digiovanni L., Good J., Salvatore D., Fenderson D., Domchek S., Stopfer J., Galantino M.L., Bryan C., Hwang W.-T. (2016). Exercise-induced dose-response alterations in adiponectin and leptin levels are dependent on body fat changes in women at risk for breast cancer. Cancer Epidemiol. Biomark. Prev..

[147] Dalamaga M., Diakopoulos K.N., Mantzoros C.S. (2012). The role of adiponectin in cancer: A review of current evidence. Endocr. Rev..

[148] Diakowska D., Markocka-Mączka K., Szelachowski P., Grabowski K. (2014). Serum levels of resistin, adiponectin, and apelin in gastroesophageal cancer patients. Dis. Markers.

[149] Wei T., Ye P., Peng X., Wu L.L., Yu G.Y. (2016). Circulating adiponectin levels in various malignancies: An updated meta-analysis of 107 studies. Oncotarget.

[150] Balstad T.R., Brunelli C., Pettersen C.H., Schønberg S.A., Skorpen F., Fallon M., Kaasa S., Bye A., Laird B.J.A., Stene G.B. (2021). Power Comparisons and Clinical Meaning of Outcome Measures in Assessing Treatment Effect in Cancer Cachexia: Secondary Analysis From a Randomized Pilot Multimodal Intervention Trial. Front. Nutr..

[151] Langer H.T., Ramsamooj S., Dantas E., Murthy A., Ahmed M., Ahmed T., Hwang S.K., Grover R., Pozovskiy R., Liang R.J. (2024). Restoring adiponectin via rosiglitazone ameliorates tissue wasting in mice with lung cancer. Acta. Physiol..

[152] Massart I.S., Kouakou A.N., Pelet N., Lause P., Schakman O., Loumaye A., Abou-Samra M., Deldicque L., Bindels L.B., Brichard S.M. (2024). Administration of adiponectin receptor agonist AdipoRon relieves cancer cachexia by mitigating inflammation in tumour-bearing mice. J. Cachexia Sarcopenia Muscle.

[153] Li Y., Onodera T., Scherer P.E. (2024). Adiponectin. Trends Endocrinol. Metab..

[154] Otu L.I., Otu A. (2021). Adiponectin and the Control of Metabolic Dysfunction: Is Exercise the Magic Bullet?. Front. Physiol..

[155] García-Hermoso A., Ramírez-Vélez R., Díez J., González A., Izquierdo M. (2023). Exercise training-induced changes in exerkine concentrations may be relevant to the metabolic control of type 2 diabetes mellitus patients: A systematic review and meta-analysis of randomized controlled trials. J. Sport. Health Sci..

[156] Polito R., Monda V., Nigro E., Messina A., Di Maio G., Giuliano M.T., Orrù S., Imperlini E., Calcagno G., Mosca L. (2020). The Important Role of Adiponectin and Orexin-A, Two Key Proteins Improving Healthy Status: Focus on Physical Activity. Front. Physiol..

[157] Chow L.S., Gerszten R.E., Taylor J.M., Pedersen B.K., van Praag H., Trappe S., Febbraio M.A., Galis Z.S., Gao Y., Haus J.M. (2022). Exerkines in health, resilience and disease. Nat. Rev. Endocrinol..

[158] Karapanagiotou E.M., Tsochatzis E.A., Dilana K.D., Tourkantonis I., Gratsias I., Syrigos K.N. (2008). The significance of leptin, adiponectin, and resistin serum levels in non-small cell lung cancer (NSCLC). Lung Cancer.

[159] Papagianni G., Panayiotou C., Vardas M., Balaskas N., Antonopoulos C., Tachmatzidis D., Didangelos T., Lambadiari V., Kadoglou N.P. (2023). The anti-inflammatory effects of aerobic exercise training in patients with type 2 diabetes: A systematic review and meta-analysis. Cytokine.

[160] Dalamaga M. (2013). Interplay of adipokines and myokines in cancer pathophysiology: Emerging therapeutic implications. World J. Exp. Med..

[161] Saeteaw M., Sanguanboonyaphong P., Yoodee J., Craft K., Sawangjit R., Ngamphaiboon N., Shantavasinkul P.C., Subongkot S., Chaiyakunapruk N. (2021). Efficacy and safety of pharmacological cachexia interventions: Systematic review and network meta-analysis. BMJ Support. Palliat. Care.

[162] Aoyagi T., Terracina K.P., Raza A., Matsubara H., Takabe K. (2015). Cancer cachexia, mechanism and treatment. World J. Gastrointest. Oncol..

[163] McTiernan A., Friedenreich C.M., Katzmarzyk P.T., Powell K.E., Macko R., Buchner D., Pescatello L.S., Bloodgood B., Tennant B., Vaux-Bjerke A. (2019). Physical Activity in Cancer Prevention and Survival: A Systematic Review. Med. Sci. Sports Exerc..

[164] Ehrman J.K., Gordon P.M., Visich P., Keteyian S.J. (2022). Clinical Exercise Physiology: Exercise Management for Chronic Diseases and Special Populations.

[165] Kamel F.H., Basha M.A., Alsharidah A.S., Salama A.B. (2020). Resistance Training Impact on Mobility, Muscle Strength and Lean Mass in Pancreatic Cancer Cachexia: A Randomized Controlled Trial. Clin. Rehabil..

[166] Solheim T.S., Laird B.J., Balstad T.R., Stene G.B., Baracos V., Bye A., Dajani O., Hendifar A.E., Strasser F., Chasen M.R. (2024). Results from a randomised, open-label trial of a multimodal intervention (exercise, nutrition and anti-inflammatory medication) plus standard care versus standard care alone to attenuate cachexia in patients with advanced cancer undergoing chemotherapy. J. Clin. Oncol..

[167] De Lazzari N., Gotte M., Kasper S., Meier E., Schuler M., Pogorzelski M., Siveke J.T., Tewes M. (2024). P-move: A randomized control trial of exercise in patients with advanced pancreatic or biliary tract cancer (aPBC) receiving beyond first-line chemotherapy. Support. Care Cancer.

[168] Hardee J.P., Counts B.R., Carson J.A. (2019). Understanding the Role of Exercise in Cancer Cachexia Therapy. Am. J. Lifestyle Med..

[169] Puppa M.J., White J.P., Velázquez K.T., Baltgalvis K.A., Sato S., Baynes J.W., Carson J.A. (2012). The effect of exercise on IL-6-induced cachexia in the ApcMin/+ mouse. J. Cachexia Sarcopenia Muscle.

[170] Lambert C.P. (2022). Resistance exercise to mitigate cancer cachexia: Molecular mechanisms and practical applications. J. Cancer Ther..

[171] Morinaga M., Sako N., Isobe M., Lee-Hotta S., Sugiura H., Kametaka S. (2021). Aerobic Exercise Ameliorates Cancer Cachexia-Induced Muscle Wasting through Adiponectin Signaling. Int. J. Mol. Sci..

[172] Pin F., Busquets S., Toledo M., Camperi A., Lopez-Soriano F.J., Costelli P., Argilés J.M., Penna F. (2015). Combination of exercise training and erythropoietin prevents cancer-induced muscle alterations. Oncotarget.

[173] Khamoui A.V., Park B.-S., Kim D.-H., Yeh M.-C., Oh S.-L., Elam M.L., Jo E., Arjmandi B.H., Salazar G., Grant S.C. (2016). Aerobic and resistance training dependent skeletal muscle plasticity in the colon-26 murine model of cancer cachexia. Metabolism.

[174] Ranjbar K., Ballarò R., Bover Q., Pin F., Beltrà M., Penna F., Costelli P. (2019). Combined exercise training positively affects muscle wasting in tumor-bearing mice. Med. Sci. Sports Exerc..

[175] Ballarò R., Penna F., Pin F., Gómez-Cabrera M.C., Viña J., Costelli P. (2019). Moderate Exercise Improves Experimental Cancer Cachexia by Modulating the Redox Homeostasis. Cancers.

[176] Wolin K.Y., Schwartz A.L., Matthews C.E., Courneya K.S., Schmitz K.H. (2012). Implementing the exercise guidelines for cancer survivors. J. Support. Oncol..

[177] Bland K.A. (2023). Evaluating the Role of Exercise as a Management Strategy to Counteract the Burden of Cancer Cachexia. Ph.D. Thesis.

[178] Bowen T.S., Schuler G., Adams V. (2015). Skeletal muscle wasting in cachexia and sarcopenia: Molecular pathophysiology and impact of exercise training. J. Cachexia Sarcopenia Muscle.

[179] Halle J.L., Counts B.R., Carson J.A. (2020). Exercise as a therapy for cancer-induced muscle wasting. Sports Med. Health Sci..

[180] Allan J., Buss L.A., Draper N., Currie M.J. (2022). Exercise in People With Cancer: A Spotlight on Energy Regulation and Cachexia. Front. Physiol..

[181] Gould D.W., Lahart I., Carmichael A.R., Koutedakis Y., Metsios G.S. (2013). Cancer cachexia prevention via physical exercise: Molecular mechanisms. J. Cachexia Sarcopenia Muscle.

[182] Arends J., Strasser F., Gonella S., Solheim T.S., Madeddu C., Ravasco P., Buonaccorso L., de van der Schueren M.A.E., Baldwin C., Chasen M. (2021). Cancer cachexia in adult patients: ESMO Clinical Practice Guidelines. ESMO Open.

[183] Muscaritoli M., Arends J., Bachmann P., Baracos V., Barthelemy N., Bertz H., Bozzetti F., Hütterer E., Isenring E., Kaasa S. (2021). ESPEN practical guideline: Clinical Nutrition in cancer. Clin. Nutr..

[184] Steindorf K., Clauss D., Wiskemann J., Schmidt M.E. (2015). Physical Activity and Gastrointestinal Cancers: Primary and Tertiary Preventive Effects and Possible Biological Mechanisms. Sports.

[185] Wiskemann J., Clauss D., Tjaden C., Hackert T., Schneider L., Ulrich C.M., Steindorf K. (2019). Progressive Resistance Training to Impact Physical Fitness and Body Weight in Pancreatic Cancer Patients: A Randomized Controlled Trial. Pancreas.

[186] Zhou X., Li S., Wang L., Wang J., Zhang P., Chen X. (2025). The emerging role of exercise preconditioning in preventing skeletal muscle atrophy. Front. Physiol..

[187] Hesketh S.J. (2024). Advancing cancer cachexia diagnosis with -omics technology and exercise as molecular medicine. Sports Med. Health Sci..

[188] Lira F.S., Yamashita A.S., Rosa J.C., Koyama C.H., Caperuto E.C., Batista M.L., Seelaender M.C. (2012). Exercise training decreases adipose tissue inflammation in cachectic rats. Horm. Metab. Res..

[189] Li J., Xie Q., Liu L., Cheng Y., Han Y., Chen X., Lin J., Li Z., Liu H., Zhang X. (2021). Swimming Attenuates Muscle Wasting and Mediates Multiple Signaling Pathways and Metabolites in CT-26 Bearing Mice. Front. Mol. Biosci..

[190] Molanouri Shamsi M., Chekachak S., Soudi S., Quinn L.S., Ranjbar K., Chenari J., Yazdi M.H., Mahdavi M. (2017). Combined effect of aerobic interval training and selenium nanoparticles on expression of IL-15 and IL-10/TNF-α ratio in skeletal muscle of 4T1 breast cancer mice with cachexia. Cytokine.

[191] Bordignon C., Dos Santos B.S., Rosa D.D. (2022). Impact of Cancer Cachexia on Cardiac and Skeletal Muscle: Role of Exercise Training. Cancers.

[192] Tsitkanou S., Koopmans P., Peterson C., Cabrera A.R., Muhyudin R., Morena F., Khadgi S., Schrems E.R., Washington T.A., Murach K.A. (2025). Myocellular adaptations to short-term weighted wheel-running exercise are largely conserved during C26-tumour induction in male and female mice. Exp. Physiol..

[193] Sato S., Gao S., Puppa M.J., Kostek M.C., Wilson L.B., Carson J.A. (2019). High-Frequency Stimulation on Skeletal Muscle Maintenance in Female Cachectic Mice. Med. Sci. Sports Exerc..

[194] Tanaka M., Sugimoto K., Fujimoto T., Xie K., Takahashi T., Akasaka H., Kurinami H., Yasunobe Y., Matsumoto T., Fujino H. (2019). Preventive effects of low-intensity exercise on cancer cachexia–induced muscle atrophy. FASEB J..

[195] Ábrigo J., Elorza A.A., Riedel C.A., Vilos C., Simon F., Cabrera D., Estrada L., Cabello-Verrugio C. (2018). Role of oxidative stress as key regulator of muscle wasting during cachexia. Oxidative Med. Cell. Longev..

[196] Leal L.G., Lopes M.A., Peres S.B., Batista M.L. (2020). Exercise Training as Therapeutic Approach in Cancer Cachexia: A Review of Potential Anti-inflammatory Effect on Muscle Wasting. Front. Physiol..

[197] Hong A.R., Kim S.W. (2018). Effects of Resistance Exercise on Bone Health. Endocrinol. Metab..

[198] Benedetti M.G., Furlini G., Zati A., Letizia Mauro G. (2018). The Effectiveness of Physical Exercise on Bone Density in Osteoporotic Patients. Biomed. Res. Int..

[199] Zhang L., Zheng Y.L., Wang R., Wang X.Q., Zhang H. (2022). Exercise for osteoporosis: A literature review of pathology and mechanism. Front. Immunol..

[200] Alves C.R., das Neves W., de Almeida N.R., Eichelberger E.J., Jannig P.R., Voltarelli V.A., Tobias G.C., Bechara L.R., de Paula Faria D., Alves M.J. (2020). Exercise training reverses cancer-induced oxidative stress and decrease in muscle COPS2/TRIP15/ALIEN. Mol. Metab..

[201] Powers S.K., Goldstein E., Schrager M., Ji L.L. (2022). Exercise Training and Skeletal Muscle Antioxidant Enzymes: An Update. Antioxidants.

[202] Linke A., Adams V., Schulze P.C., Erbs S., Gielen S., Fiehn E., Möbius-Winkler S., Schubert A., Schuler G., Hambrecht R. (2005). Antioxidative effects of exercise training in patients with chronic heart failure: Increase in radical scavenger enzyme activity in skeletal muscle. Circulation.

[203] Brinkmann C., Chung N., Schmidt U., Kreutz T., Lenzen E., Schiffer T., Geisler S., Graf C., Montiel-Garcia G., Renner R. (2012). Training alters the skeletal muscle antioxidative capacity in non-insulin-dependent type 2 diabetic men. Scand. J. Med. Sci. Sports.

[204] Powers S.K., Jackson M.J. (2008). Exercise-induced oxidative stress: Cellular mechanisms and impact on muscle force production. Physiol. Rev..

[205] Powers S.K., Criswell D., Lawler J., Ji L.L., Martin D., Herb R.A., Dudley G. (1994). Influence of exercise and fiber type on antioxidant enzyme activity in rat skeletal muscle. Am. J. Physiol..

[206] Gomes M.J., Pagan L.U., Lima A.R.R., Reyes D.R.A., Martinez P.F., Damatto F.C., Pontes T.H.D., Rodrigues E.A., Souza L.M., Tosta I.F. (2020). Effects of aerobic and resistance exercise on cardiac remodelling and skeletal muscle oxidative stress of infarcted rats. J. Cell Mol. Med..

[207] Scheffer D.L., Silva L.A., Tromm C.B., da Rosa G.L., Silveira P.C., de Souza C.T., Latini A., Pinho R.A. (2012). Impact of different resistance training protocols on muscular oxidative stress parameters. Appl. Physiol. Nutr. Metab..

[208] de Sousa C.V., Sales M.M., Rosa T.S., Lewis J.E., de Andrade R.V., Simões H.G. (2017). The antioxidant effect of exercise: A systematic review and meta-analysis. Sports Med..

[209] Aquila G., Re Cecconi A.D., Brault J.J., Corli O., Piccirillo R. (2020). Nutraceuticals and Exercise against Muscle Wasting during Cancer Cachexia. Cells.

[210] Ballarò R., Beltrà M., De Lucia S., Pin F., Ranjbar K., Hulmi J.J., Costelli P., Penna F. (2019). Moderate exercise in mice improves cancer plus chemotherapy-induced muscle wasting and mitochondrial alterations. FASEB J..

[211] Longobucco Y., Masini A., Marini S., Barone G., Fimognari C., Bragonzoni L., Dallolio L., Maffei F. (2022). Exercise and Oxidative Stress Biomarkers among Adult with Cancer: A Systematic Review. Oxidative Med. Cell. Longev..

[212] Novelli G., Calcaterra G., Casciani F., Pecorelli S., Mehta J.L. (2024). ‘Exerkines’: A Comprehensive Term for the Factors Produced in Response to Exercise. Biomedicines.

[213] Heo J., Noble E.E., Call J.A. (2023). The role of exerkines on brain mitochondria: A mini-review. J. Appl. Physiol..

[214] Watkins B.A., Smith B.J., Volpe S.L., Shen C.-L. (2024). Exerkines, Nutrition, and Systemic Metabolism. Nutrients.

[215] Brooks G.A., Osmond A.D., Arevalo J.A., Duong J.J., Curl C.C., Moreno-Santillan D.D., Leija R.G. (2023). Lactate as a myokine and exerkine: Drivers and signals of physiology and metabolism. J. Appl. Physiol..

[216] Safdar A., Saleem A., Tarnopolsky M.A. (2016). The potential of endurance exercise-derived exosomes to treat metabolic diseases. Nat. Rev. Endocrinol..

[217] Pedersen B.K., Steensberg A., Keller P., Keller C., Fischer C., Hiscock N., van Hall G., Plomgaard P., Febbraio M.A. (2003). Muscle-derived interleukin-6: Lipolytic, anti-inflammatory and immune regulatory effects. Pflügers Arch..

[218] Pedersen B.K., Febbraio M.A. (2012). Muscles, exercise and obesity: Skeletal muscle as a secretory organ. Nat. Rev. Endocrinol..

[219] Walzik D., Wences-Chirino T., Zimmer P., Joisten N. (2024). Molecular insights of exercise therapy in disease prevention and treatment. Signal Transduct. Target. Ther..

[220] Safdar A., Tarnopolsky M.A. (2018). Exosomes as Mediators of the Systemic Adaptations to Endurance Exercise. Cold Spring Harb. Perspect. Med..

[221] Nederveen J.P., Warnier G., Di Carlo A., Nilsson M.I., Tarnopolsky M.A. (2020). Extracellular Vesicles and Exosomes: Insights From Exercise Science. Front. Physiol..

[222] Liang Y.-Y., Zhang L.-D., Luo X., Wu L.-L., Chen Z.-W., Wei G.-H., Zhang K.-Q., Du Z.-A., Li R.-Z., So K.-F. (2022). All roads lead to Rome—A review of the potential mechanisms by which exerkines exhibit neuroprotective effects in Alzheimer’s disease. Neural Regen. Res..

[223] Zhou N., Gong L., Zhang E., Wang X. (2024). Exploring exercise-driven exerkines: Unraveling the regulation of metabolism and inflammation. PeerJ.

[224] Katz D.H., Lindholm M.E., Ashley E.A. (2025). Charting the Molecular Terrain of Exercise: Energetics, Exerkines, and the Future of Multiomic Mapping. Physiology.

[225] Goj T., Hoene M., Fritsche L., Schneeweiss P., Machann J., Petrera A., Hauck S.M., Fritsche A., Birkenfeld A.L., Peter A. (2022). The Acute Cytokine Response to 30-Minute Exercise Bouts Before and After 8-Week Endurance Training in Individuals With Obesity. J. Clin. Endocrinol. Metab..

[226] Nielsen S., Akerstrom T., Rinnov A., Yfanti C., Scheele C., Pedersen B.K., Laye M.J. (2014). The miRNA plasma signature in response to acute aerobic exercise and endurance training. PLoS ONE.

[227] Ren Y., Zhao H., Yin C., Lan X., Wu L., Du X., Griffiths H.R., Gao D. (2022). Adipokines, Hepatokines and Myokines: Focus on Their Role and Molecular Mechanisms in Adipose Tissue Inflammation. Front. Endocrinol..

[228] Zunner B.E.M., Wachsmuth N.B., Eckstein M.L., Scherl L., Schierbauer J.R., Haupt S., Stumpf C., Reusch L., Moser O. (2022). Myokines and Resistance Training: A Narrative Review. Int. J. Mol. Sci..

[229] Ercan Z., Deniz G., Yentur S.B., Arikan F.B., Karatas A., Alkan G., Koca S.S. (2023). Effects of acute aerobic exercise on cytokines, klotho, irisin, and vascular endothelial growth factor responses in rheumatoid arthritis patients. Ir. J. Med. Sci..

[230] Fang P., She Y., Yu M., Min W., Shang W., Zhang Z. (2023). Adipose-Muscle crosstalk in age-related metabolic disorders: The emerging roles of adipo-myokines. Ageing Res. Rev..

[231] Brenner D.R., Ruan Y., Adams S.C., Courneya K.S., Friedenreich C.M. (2019). The impact of exercise on growth factors (VEGF and FGF2): Results from a 12-month randomized intervention trial. Eur. Rev. Aging Phys. Act..

[232] Mathes S., Fahrner A., Ghoshdastider U., Rüdiger H.A., Leunig M., Wolfrum C., Krützfeldt J. (2021). FGF-2–dependent signaling activated in aged human skeletal muscle promotes intramuscular adipogenesis. Proc. Natl. Acad. Sci. USA.

[233] Bettariga F., Taaffe D.R., Galvão D.A., Lopez P., Bishop C., Markarian A.M., Natalucci V., Kim J.-S., Newton R.U. (2024). Exercise training mode effects on myokine expression in healthy adults: A systematic review with meta-analysis. J. Sport. Health Sci..

[234] Rodriguez A., Becerril S., Hernandez-Pardos A.W., Fruhbeck G. (2020). Adipose tissue depot differences in adipokines and effects on skeletal and cardiac muscle. Curr. Opin. Pharmacol..

[235] Al-Ibraheem A.M.T., Hameed A.T.A.Z., Marsool M.D.M., Jain H., Prajjwal P., Khazmi I., Nazzal R.S., AL-Najati H.M.H., Al-Zuhairi B.H.Y.K., Razzaq M. (2024). Exercise-Induced cytokines, diet, and inflammation and their role in adipose tissue metabolism. Health Sci. Rep..

[236] Mu W.-J., Zhu J.-Y., Chen M., Guo L. (2021). Exercise-mediated browning of white adipose tissue: Its significance, mechanism and effectiveness. Int. J. Mol. Sci..

[237] Dumond Bourie A., Potier J.-B., Pinget M., Bouzakri K. (2023). Myokines: Crosstalk and consequences on liver physiopathology. Nutrients.

[238] Jin L., Diaz-Canestro C., Wang Y., Tse M.A., Xu A. (2024). Exerkines and cardiometabolic benefits of exercise: From bench to clinic. EMBO Mol. Med..

[239] Wang Z., Sun T., Yu J., Li S., Gong L., Zhang Y. (2023). FGF21: A Sharp Weapon in the Process of Exercise to Improve NAFLD. FBL.

[240] Kirk B., Feehan J., Lombardi G., Duque G. (2020). Muscle, Bone, and Fat Crosstalk: The Biological Role of Myokines, Osteokines, and Adipokines. Curr. Osteoporos. Rep..

[241] Kornel A., Den Hartogh D.J., Klentrou P., Tsiani E. (2021). Role of the myokine irisin on bone homeostasis: Review of the current evidence. Int. J. Mol. Sci..

[242] Prideaux M., Smargiassi A., Peng G., Brotto M., Robling A.G., Bonewald L.F. (2023). L BAIBA Synergizes with Sub Optimal Mechanical Loading to Promote New Bone Formation. J. Bone Miner. Res. Plus.

[243] Shimonty A., Bonewald L.F., Huot J.R. (2023). Metabolic Health and Disease: A Role of Osteokines?. Calcif. Tissue Int..

[244] Agudelo L.Z., Femenia T., Orhan F., Porsmyr-Palmertz M., Goiny M., Martinez-Redondo V., Correia J.C., Izadi M., Bhat M., Schuppe-Koistinen I. (2014). Skeletal muscle PGC-1alpha1 modulates kynurenine metabolism and mediates resilience to stress-induced depression. Cell.

[245] Yu Q., Zhang Z., Herold F., Ludyga S., Kuang J., Chen Y., Liu Z., Erickson K.I., Goodpaster B.H., Cheval B. (2025). Physical activity, cathepsin B, and cognitive health. Trends Mol. Med..

[246] Schön M., Kovaničová Z., Košutzká Z., Nemec M., Tomková M., Jacková L., Máderová D., Slobodová L., Valkovič P., Ukropec J. (2019). Effects of running on adiponectin, insulin and cytokines in cerebrospinal fluid in healthy young individuals. Sci. Rep..

[247] Leiter O., Lowe J., Brici D., Walker T.L. (2024). Exerkines and brain rejuvenation. Alzheimer’s Dement..

[248] Wu Y.S., Zhu B., Luo A.L., Yang L., Yang C. (2018). The Role of Cardiokines in Heart Diseases: Beneficial or Detrimental?. Biomed. Res. Int..

[249] Wang Y., Tian M., Mi C., Chen K., Ji Y., Wang L., Zhang J., Cheng K. (2020). Exercise protects the heart against myocardial infarction through upregulation of miR-1192. Biochem. Biophys. Res. Commun..

[250] Ramírez-Vélez R., González A., García-Hermoso A., Amézqueta I.L., Izquierdo M., Díez J. (2023). Revisiting skeletal myopathy and exercise training in heart failure: Emerging role of myokines. Metabolism.

[251] Steensberg A., Van Hall G., Osada T., Sacchetti M., Saltin B., Pedersen B.K. (2020). Production of interleukin-6 in contracting human skeletal muscles can account for the exercise-induced increase in plasma interleukin-6. J. Physiol..

[252] Goussetis E., Spiropoulos A., Tsironi M., Skenderi K., Margeli A., Graphakos S., Baltopoulos P., Papassotiriou I. (2009). Spartathlon, a 246 kilometer foot race: Effects of acute inflammation induced by prolonged exercise on circulating progenitor reparative cells. Blood Cells Mol. Dis..

[253] Nash D., Hughes M.G., Butcher L., Aicheler R., Smith P., Cullen T., Webb R. (2023). IL-6 signaling in acute exercise and chronic training: Potential consequences for health and athletic performance. Scand. J. Med. Sci. Sports.

[254] Song M., Tang Y., Cao K., Qi L., Xie K. (2024). Unveiling the role of interleukin-6 in pancreatic cancer occurrence and progression. Front. Endocrinol..

[255] Poulia K.A., Sarantis P., Antoniadou D., Koustas E., Papadimitropoulou A., Papavassiliou A.G., Karamouzis M.V. (2020). Pancreatic Cancer and Cachexia-Metabolic Mechanisms and Novel Insights. Nutrients.

[256] van Duijneveldt G., Griffin M.D.W., Putoczki T.L. (2020). Emerging roles for the IL-6 family of cytokines in pancreatic cancer. Clin. Sci..

[257] Croft L., Bartlett J.D., MacLaren D.P., Reilly T., Evans L., Mattey D.L., Nixon N.B., Drust B., Morton J.P. (2009). High-intensity interval training attenuates the exercise-induced increase in plasma IL-6 in response to acute exercise. Appl. Physiol. Nutr. Metab..

[258] Zheng G., Qiu P., Xia R., Lin H., Ye B., Tao J., Chen L. (2019). Effect of aerobic exercise on inflammatory markers in healthy middle-aged and older adults: A systematic review and meta-analysis of randomized controlled trials. Front. Aging Neurosci..

[259] Hamer M., Sabia S., Batty G.D., Shipley M.J., Tabák A.G., Singh-Manoux A., Kivimaki M. (2012). Physical activity and inflammatory markers over 10 years: Follow-up in men and women from the Whitehall II cohort study. Circulation.

[260] Mera P., Laue K., Ferron M., Confavreux C., Wei J., Galán-Díez M., Lacampagne A., Mitchell S.J., Mattison J.A., Chen Y. (2016). Osteocalcin Signaling in Myofibers Is Necessary and Sufficient for Optimum Adaptation to Exercise. Cell Metab..

[261] Chowdhury S., Schulz L., Palmisano B., Singh P., Berger J.M., Yadav V.K., Mera P., Ellingsgaard H., Hidalgo J., Brüning J. (2020). Muscle-derived interleukin 6 increases exercise capacity by signaling in osteoblasts. J. Clin. Investig..

[262] Prystaz K., Kaiser K., Kovtun A., Haffner-Luntzer M., Fischer V., Rapp A.E., Liedert A., Strauss G., Waetzig G.H., Rose-John S. (2018). Distinct Effects of IL-6 Classic and Trans-Signaling in Bone Fracture Healing. Am. J. Pathol..

[263] Wang J., Chang C.Y., Yang X., Zhou F., Liu J., Feng Z., Hu W. (2023). Leukemia inhibitory factor, a double-edged sword with therapeutic implications in human diseases. Mol. Ther..

[264] Zeng R., Tong C., Xiong X. (2022). The Molecular Basis and Therapeutic Potential of Leukemia Inhibitory Factor in Cancer Cachexia. Cancers.

[265] Seto D.N., Kandarian S.C., Jackman R.W. (2015). A Key Role for Leukemia Inhibitory Factor in C26 Cancer Cachexia. J. Biol. Chem..

[266] Broholm C., Pedersen B.K. (2010). Leukaemia inhibitory factor--an exercise-induced myokine. Exerc. Immunol. Rev..

[267] Broholm C., Laye M.J., Brandt C., Vadalasetty R., Pilegaard H., Pedersen B.K., Scheele C. (2011). LIF is a contraction-induced myokine stimulating human myocyte proliferation. J. Appl. Physiol..

[268] Broholm C., Brandt C., Schultz N.S., Nielsen A.R., Pedersen B.K., Scheele C. (2012). Deficient leukemia inhibitory factor signaling in muscle precursor cells from patients with type 2 diabetes. Am. J. Physiol.—Endocrinol. Metab..

[269] Minniti G., Pescinini-Salzedas L.M., Minniti G., Laurindo L.F., Barbalho S.M., Vargas Sinatora R., Sloan L.A., Haber R.S.A., Araújo A.C., Quesada K. (2022). Organokines, Sarcopenia, and Metabolic Repercussions: The Vicious Cycle and the Interplay with Exercise. Int. J. Mol. Sci..

[270] Yang X., Wang J., Chang C.-Y., Zhou F., Liu J., Xu H., Ibrahim M., Gomez M., Guo G.L., Liu H. (2024). Leukemia inhibitory factor suppresses hepatic de novo lipogenesis and induces cachexia in mice. Nat. Commun..

[271] Agca S., Kir S. (2024). The role of interleukin-6 family cytokines in cancer cachexia. FEBS J..

[272] McPherron A.C., Lawler A.M., Lee S.-J. (1997). Regulation of skeletal muscle mass in mice by a new TGF-p superfamily member. Nature.

[273] Esposito P., Picciotto D., Battaglia Y., Costigliolo F., Viazzi F., Verzola D. (2022). Myostatin: Basic biology to clinical application. Adv. Clin. Chem..

[274] Elkina Y., von Haehling S., Anker S.D., Springer J. (2011). The role of myostatin in muscle wasting: An overview. J. Cachexia Sarcopenia Muscle.

[275] Parfenova O.K., Kukes V.G., Grishin D.V. (2021). Follistatin-Like Proteins: Structure, Functions and Biomedical Importance. Biomedicines.

[276] Suh J., Lee Y.S. (2020). Myostatin Inhibitors: Panacea or Predicament for Musculoskeletal Disorders?. J. Bone Metab..

[277] Lee S.J., Lehar A., Meir J.U., Koch C., Morgan A., Warren L.E., Rydzik R., Youngstrom D.W., Chandok H., George J. (2020). Targeting myostatin/activin A protects against skeletal muscle and bone loss during spaceflight. Proc. Natl. Acad. Sci. USA.

[278] Hittel D.S., Axelson M., Sarna N., Shearer J., Huffman K.M., Kraus W.E. (2010). Myostatin decreases with aerobic exercise and associates with insulin resistance. Med. Sci. Sports Exerc..

[279] Setiawan I., Sanjaya A., Lesmana R., Yen P.M., Goenawan H. (2021). Hippo pathway effectors YAP and TAZ and their association with skeletal muscle ageing. J. Physiol. Biochem..

[280] Kawaguchi Y., Watanabe A., Shiratori T., Kaku R., Ueda K., Okamoto K., Kataoka Y., Ohshio Y., Hanaoka J. (2024). Myostatin expression in lung cancer induces sarcopenia and promotes cancer progression. Gen. Thorac. Cardiovasc. Surg..

[281] Willoughby D.S., Cardaci T.D., Machek S.B., Wilburn D.T., Heileson J.L. (2022). Resistance Exercise-Induced Increases in Muscle Myostatin mRNA and Protein Expression Are Subsequently Decreased in Circulation in the Presence of Increased Levels of the Extracellular Matrix Stabilizing Protein Decorin. J. Sports Sci. Med..

[282] Khalafi M., Aria B., Symonds M.E., Rosenkranz S.K. (2023). The effects of resistance training on myostatin and follistatin in adults: A systematic review and meta-analysis. Physiol. Behav..

[283] Ayaz E.Y., Dincer B., Cinbaz G., Karacan E., Benli R.K., Mete E., Bilgiç H., Mesci B. (2025). The Effect of Exercise on Spexin and Follistatin in Elderly Individuals. J. Cachexia Sarcopenia Muscle.

[284] Hansen J., Brandt C., Nielsen A.R., Hojman P., Whitham M., Febbraio M.A., Pedersen B.K., Plomgaard P. (2011). Exercise induces a marked increase in plasma follistatin: Evidence that follistatin is a contraction-induced hepatokine. Endocrinology.

[285] Korzun T., Moses A.S., Kim J., Patel S., Schumann C., Levasseur P.R., Diba P., Olson B., Rebola K.G.O., Norgard M. (2022). Nanoparticle-Based Follistatin Messenger RNA Therapy for Reprogramming Metastatic Ovarian Cancer and Ameliorating Cancer-Associated Cachexia. Small.

[286] Ataeinosrat A., Saeidi A., Abednatanzi H., Rahmani H., Daloii A.A., Pashaei Z., Hojati V., Basati G., Mossayebi A., Laher I. (2022). Intensity Dependent Effects of Interval Resistance Training on Myokines and Cardiovascular Risk Factors in Males With Obesity. Front. Endocrinol..

[287] Broniec M.N., Norland K., Thomas J., Wang X., Harris R.A. (2024). The decorin and myostatin response to acute whole body vibration: Impact of adiposity, sex, and race. Int. J. Obes..

[288] Miura T., Kishioka Y., Wakamatsu J.-I., Hattori A., Hennebry A., Berry C., Sharma M., Kambadur R., Nishimura T. (2006). Decorin Binds Myostatin and Modulates Its Activity to Muscle Cells. Biochem. Biophys. Res. Commun..

[289] Xie C., Mondal D.K., Ulas M., Neill T., Iozzo R.V. (2022). Oncosuppressive roles of decorin through regulation of multiple receptors and diverse signaling pathways. Am. J. Physiol. Cell Physiol..

[290] Appunni S., Saxena A., Ramamoorthy V., Zhang Y., Doke M., Nair S.S., Khosla A.A., Rubens M. (2025). Decorin: Matrix-based pan-cancer tumor suppressor. Mol. Cell Biochem..

[291] Kawaguchi T., Yoshio S., Sakamoto Y., Hashida R., Koya S., Hirota K., Nakano D., Yamamura S., Niizeki T., Matsuse H. (2020). Impact of Decorin on the Physical Function and Prognosis of Patients with Hepatocellular Carcinoma. J. Clin. Med..

[292] Xu W., Neill T., Yang Y., Hu Z., Cleveland E., Wu Y., Hutten R., Xiao X., Stock S.R., Shevrin D. (2015). The systemic delivery of an oncolytic adenovirus expressing decorin inhibits bone metastasis in a mouse model of human prostate cancer. Gene Ther..

[293] Adams G.R. (2002). Invited Review: Autocrine/paracrine IGF-I and skeletal muscle adaptation. J. Appl. Physiol..

[294] Frystyk J. (2010). Exercise and the Growth Hormone-Insulin-Like Growth Factor Axis. Med. Sci. Sports Exerc..

[295] Philippou A., Maridaki M., Halapas A., Koutsilieris M. (2007). The role of the insulin-like growth factor 1 (IGF-1) in skeletal muscle physiology. In Vivo.

[296] Wall B.T., Gorissen S.H., Pennings B., Koopman R., Groen B.B., Verdijk L.B., van Loon L.J. (2015). Aging Is Accompanied by a Blunted Muscle Protein Synthetic Response to Protein Ingestion. PLoS ONE.

[297] Arazi H., Babaei P., Moghimi M., Asadi A. (2021). Acute effects of strength and endurance exercise on serum BDNF and IGF-1 levels in older men. BMC Geriatr..

[298] Moore D.R., McKay B.R., Tarnopolsky M.A., Parise G. (2018). Blunted satellite cell response is associated with dysregulated IGF-1 expression after exercise with age. Eur. J. Appl. Physiol..

[299] Philippou A., Papageorgiou E., Bogdanis G., Halapas A., Sourla A., Maridaki M., Pissimissis N., Koutsilieris M. (2009). Expression of IGF-1 isoforms after exercise-induced muscle damage in humans: Characterization of the MGF E peptide actions in vitro. In Vivo.

[300] Yang S., Alnaqeeb M., Simpson H., Goldspink G. (1996). Cloning and characterization of an IGF-1 isoform expressed in skeletal muscle subjected to stretch. J. Muscle Res. Cell Motil..

[301] Kawai M., Rosen C.J. (2012). The insulin-like growth factor system in bone: Basic and clinical implications. Endocrinol. Metab. Clin. N. Am..

[302] de Alcantara Borba D., da Silva Alves E., Rosa J.P.P., Facundo L.A., Costa C.M.A., Silva A.C., Narciso F.V., Silva A., de Mello M.T. (2020). Can IGF-1 Serum Levels Really be Changed by Acute Physical Exercise? A Systematic Review and Meta-Analysis. J. Phys. Act. Health.

[303] Colleluori G., Aguirre L., Phadnis U., Fowler K., Armamento-Villareal R., Sun Z., Brunetti L., Hyoung Park J., Kaipparettu B.A., Putluri N. (2019). Aerobic Plus Resistance Exercise in Obese Older Adults Improves Muscle Protein Synthesis and Preserves Myocellular Quality Despite Weight Loss. Cell Metab..

[304] Dreher S.I., Grubba P., von Toerne C., Moruzzi A., Maurer J., Goj T., Birkenfeld A.L., Peter A., Loskill P., Hauck S.M. (2024). IGF1 promotes human myotube differentiation toward a mature metabolic and contractile phenotype. Am. J. Physiol.—Cell Physiol..

[305] Yoshida T., Delafontaine P. (2020). Mechanisms of IGF-1-Mediated Regulation of Skeletal Muscle Hypertrophy and Atrophy. Cells.

[306] Costelli P., Muscaritoli M., Bossola M., Penna F., Reffo P., Bonetto A., Busquets S., Bonelli G., Lopez-Soriano F.J., Doglietto G.B. (2006). IGF-1 is downregulated in experimental cancer cachexia. Am. J. Physiol.—Regul. Integr. Comp. Physiol..

[307] Schmidt K., von Haehling S., Doehner W., Palus S., Anker S.D., Springer J. (2011). IGF-1 treatment reduces weight loss and improves outcome in a rat model of cancer cachexia. J. Cachexia Sarcopenia Muscle.

[308] Khalafi M., Maleki A.H., Symonds M.E., Sakhaei M.H., Rosenkranz S.K., Ehsanifar M., Korivi M., Liu Y. (2023). Interleukin-15 responses to acute and chronic exercise in adults: A systematic review and meta-analysis. Front. Immunol..

[309] Nielsen A.R., Pedersen B.K. (2007). The biological roles of exercise-induced cytokines: IL-6, IL-8, and IL-15. Appl. Physiol. Nutr. Metab..

[310] Kim H.J., Park J.Y., Oh S.L., Kim Y.A., So B., Seong J.K., Song W. (2013). Effect of treadmill exercise on interleukin-15 expression and glucose tolerance in zucker diabetic Fatty rats. Diabetes Metab. J..

[311] Martínez-Hernández P.L., Hernanz-Macías Á., Gómez-Candela C., Grande-Aragón C., Feliu-Batlle J., Castro-Carpeño J., Martínez-Muñoz I., Zurita-Rosa L., Villarino-Sanz M., Prados-Sánchez C. (2012). Serum interleukin-15 levels in cancer patients with cachexia. Oncol. Rep..

[312] Carbó N., López-Soriano J., Costelli P., Busquets S., Alvarez B., Baccino F.M., Quinn L.S., López-Soriano F.J., Argilés J.M. (2000). Interleukin-15 antagonizes muscle protein waste in tumour-bearing rats. Br. J. Cancer.

[313] Duan Z., Yang Y., Qin M., Yi X. (2024). Interleukin 15: A new intermediary in the effects of exercise and training on skeletal muscle and bone function. J. Cell Mol. Med..

[314] Bostrom P., Wu J., Jedrychowski M.P., Korde A., Ye L., Lo J.C., Rasbach K.A., Bostrom E.A., Choi J.H., Long J.Z. (2012). A PGC1-alpha-dependent myokine that drives brown-fat-like development of white fat and thermogenesis. Nature.

[315] Maak S., Norheim F., Drevon C.A., Erickson H.P. (2021). Progress and Challenges in the Biology of FNDC5 and Irisin. Endocr. Rev..

[316] Liu S., Cui F., Ning K., Wang Z., Fu P., Wang D., Xu H. (2022). Role of irisin in physiology and pathology. Front. Endocrinol..

[317] Us Altay D., Keha E.E., Ozer Yaman S., Ince I., Alver A., Erdogan B., Canpolat S., Cobanoglu U., Mentese A. (2016). Investigation of the expression of irisin and some cachectic factors in mice with experimentally induced gastric cancer. Qjm.

[318] Kim H., Wrann C.D., Jedrychowski M., Vidoni S., Kitase Y., Nagano K., Zhou C., Chou J., Parkman V.A., Novick S.J. (2019). Irisin Mediates Effects on Bone and Fat via αV Integrin Receptors. Cell.

[319] Graca F.A., Rai M., Hunt L.C., Stephan A., Wang Y.-D., Gordon B., Wang R., Quarato G., Xu B., Fan Y. (2022). The myokine Fibcd1 is an endogenous determinant of myofiber size and mitigates cancer-induced myofiber atrophy. Nat. Commun..

[320] Luo J., Liu W., Feng F., Chen L. (2021). Apelin/APJ system: A novel therapeutic target for locomotor system diseases. Eur. J. Pharmacol..

[321] Ligetvári R., Szokodi I., Far G., Csöndör É., Móra Á., Komka Z., Tóth M., Oláh A., Ács P. (2023). Apelin as a Potential Regulator of Peak Athletic Performance. Int. J. Mol. Sci..

[322] Son J.S., Zhao L., Chen Y., Chen K., Chae S.A., de Avila J.M., Wang H., Zhu M.J., Jiang Z., Du M. (2020). Maternal exercise via exerkine apelin enhances brown adipogenesis and prevents metabolic dysfunction in offspring mice. Sci. Adv..

[323] Kim K.H., Lee M.S. (2014). FGF21 as a Stress Hormone: The Roles of FGF21 in Stress Adaptation and the Treatment of Metabolic Diseases. Diabetes Metab. J..

[324] Cuevas-Ramos D., Almeda-Valdes P., Meza-Arana C.E., Brito-Cordova G., Gomez-Perez F.J., Mehta R., Oseguera-Moguel J., Aguilar-Salinas C.A. (2012). Exercise increases serum fibroblast growth factor 21 (FGF21) levels. PLoS ONE.

[325] Jin L., Geng L., Ying L., Lingling S., Ye K., Yang R., Liu Y., Wang Y., Cai Y., Jiang X. (2022). FGF21-Sirtuin 3 Axis Confers the Protective Effects of Exercise Against Diabetic Cardiomyopathy by Governing Mitochondrial Integrity. Circulation.

[326] Liu C., Yan X., Zong Y., He Y., Yang G., Xiao Y., Wang S. (2024). The effects of exercise on FGF21 in adults: A systematic review and meta-analysis. PeerJ.

[327] Franz K., Ost M., Otten L., Herpich C., Coleman V., Endres A.-S., Klaus S., Müller-Werdan U., Norman K. (2019). Higher serum levels of fibroblast growth factor 21 in old patients with cachexia. Nutrition.

[328] Oost L.J., Kustermann M., Armani A., Blaauw B., Romanello V. (2019). Fibroblast growth factor 21 controls mitophagy and muscle mass. J. Cachexia Sarcopenia Muscle.

[329] Kleinert M., Clemmensen C., Sjøberg K.A., Carl C.S., Jeppesen J.F., Wojtaszewski J.F.P., Kiens B., Richter E.A. (2018). Exercise increases circulating GDF15 in humans. Mol. Metab..

[330] Klein A.B., Nicolaisen T.S., Ørtenblad N., Gejl K.D., Jensen R., Fritzen A.M., Larsen E.L., Karstoft K., Poulsen H.E., Morville T. (2021). Pharmacological but not physiological GDF15 suppresses feeding and the motivation to exercise. Nat. Commun..

[331] Klein A.B., Kleinert M., Richter E.A., Clemmensen C. (2022). GDF15 in Appetite and Exercise: Essential Player or Coincidental Bystander?. Endocrinology.

[332] Ling T., Zhang J., Ding F., Ma L. (2023). Role of growth differentiation factor 15 in cancer cachexia (Review). Oncol. Lett..

[333] Ramos C.C., Pires J., Gonzalez E., Garcia-Vallicrosa C., Reis C.A., Falcon-Perez J.M., Freitas D. (2024). Extracellular vesicles in tumor-adipose tissue crosstalk: Key drivers and therapeutic targets in cancer cachexia. Extracell. Vesicles Circ. Nucleic Acids.

[334] Greenhill C. (2016). Osteocalcin in the adaptation to exercise. Nat. Rev. Endocrinol..

[335] Cariati I., Bonanni R., Onorato F., Mastrogregori A., Rossi D., Iundusi R., Gasbarra E., Tancredi V., Tarantino U. (2021). Role of Physical Activity in Bone-Muscle Crosstalk: Biological Aspects and Clinical Implications. J. Funct. Morphol. Kinesiol..

[336] Mohammad Rahimi G.R., Niyazi A., Alaee S. (2021). The effect of exercise training on osteocalcin, adipocytokines, and insulin resistance: A systematic review and meta-analysis of randomized controlled trials. Osteoporos. Int..

[337] Mera P., Laue K., Wei J., Berger J.M., Karsenty G. (2016). Osteocalcin is necessary and sufficient to maintain muscle mass in older mice. Mol. Metab..

[338] Colucci-D’Amato L., Speranza L., Volpicelli F. (2020). Neurotrophic Factor BDNF, Physiological Functions and Therapeutic Potential in Depression, Neurodegeneration and Brain Cancer. Int. J. Mol. Sci..

[339] McKay B.R., Nederveen J.P., Fortino S.A., Snijders T., Joanisse S., Kumbhare D.A., Parise G. (2020). Brain-derived neurotrophic factor is associated with human muscle satellite cell differentiation in response to muscle-damaging exercise. Appl. Physiol. Nutr. Metab..

[340] Yu T., Chang Y., Gao X.L., Li H., Zhao P. (2017). Dynamic Expression and the Role of BDNF in Exercise-induced Skeletal Muscle Regeneration. Int. J. Sports Med..

[341] Matthews V.B., Astrom M.B., Chan M.H., Bruce C.R., Krabbe K.S., Prelovsek O., Akerstrom T., Yfanti C., Broholm C., Mortensen O.H. (2009). Brain-derived neurotrophic factor is produced by skeletal muscle cells in response to contraction and enhances fat oxidation via activation of AMP-activated protein kinase. Diabetologia.

[342] Chan W.S., Ng C.F., Pang B.P.S., Hang M., Tse M.C.L., Iu E.C.Y., Ooi X.C., Yang X., Kim J.K., Lee C.W. (2024). Exercise-induced BDNF promotes PPARδ-dependent reprogramming of lipid metabolism in skeletal muscle during exercise recovery. Sci. Signal.

[343] Chang H., Kwon O., Shin M.-S., Kang G.M., Leem Y.H., Lee C.H., Kim S.J., Roh E., Kim H.-K., Youn B.-S. (2018). Role of Angptl4/Fiaf in exercise-induced skeletal muscle AMPK activation. J. Appl. Physiol..

[344] Ingerslev B., Schiøler Hansen J., Hoffmann C., Clemmesen J., Secher N., Scheler M., Angelis M., Häring H., Pedersen B., Weigert C. (2017). Angiopoietin-like Protein 4 Is an Exercise-induced Hepatokine in Humans, Regulated by Glucagon and cAMP. Mol. Metab..

[345] Neto N.I.P., Boldarine V.T., Hachul A.C.L., Oyama L.M., Lima J.D.C.C., Fernandez E.S., Otoch J.P., de Alcântara P.S.M., Tokeshi F., Seelaender M.C.L. (2019). Association between ANGPTL-4 and the proinflammatory process in cancer cachexia patients. Oncotarget.

[346] Han J., Wang Y., Qiu Y., Sun D., Liu Y., Li Z., Zhou B., Zhang H., Xiao Y., Wu G. (2022). Single-cell sequencing unveils key contributions of immune cell populations in cancer-associated adipose wasting. Cell Discov..

[347] Park M.S., Kim S.E., Lee P., Lee J.H., Jung K.H., Hong S.S. (2024). Potential role of ANGPTL4 in cancer progression, metastasis, and metabolism: A brief review. BMB Rep..

[348] Li L., Foo B.J.W., Kwok K.W., Sakamoto N., Mukae H., Izumikawa K., Mandard S., Quenot J.P., Lagrost L., Teh W.K. (2019). Antibody Treatment against Angiopoietin-Like 4 Reduces Pulmonary Edema and Injury in Secondary Pneumococcal Pneumonia. mBio.

[349] Zuo Y., He Z., Chen Y., Dai L. (2023). Dual role of ANGPTL4 in inflammation. Inflamm. Res..

[350] Mandadzhiev N. (2025). The contemporary role of lactate in exercise physiology and exercise prescription—A review of the literature. Folia Medica.

[351] Liu X., Li S., Cui Q., Guo B., Ding W., Liu J., Quan L., Li X., Xie P., Jin L. (2024). Activation of GPR81 by lactate drives tumour-induced cachexia. Nat. Metab..

[352] Maurer J., Hoene M., Weigert C. (2021). Signals from the Circle: Tricarboxylic Acid Cycle Intermediates as Myometabokines. Metabolites.

[353] Shi Y., Zhou D., Wang H., Huang L., Gao X., Maitiabula G., Zhang L., Wang X. (2025). Succinate Regulates Exercise-Induced Muscle Remodelling by Boosting Satellite Cell Differentiation Through Succinate Receptor 1. J. Cachexia Sarcopenia Muscle.

[354] Huang H., Li G., He Y., Chen J., Yan J., Zhang Q., Li L., Cai X. (2024). Cellular succinate metabolism and signaling in inflammation: Implications for therapeutic intervention. Front. Immunol..

[355] Roberts L.D., Boström P., O’Sullivan J.F., Schinzel R.T., Lewis G.D., Dejam A., Lee Y.-K., Palma M.J., Calhoun S., Georgiadi A. (2014). β-Aminoisobutyric acid induces browning of white fat and hepatic β-oxidation and is inversely correlated with cardiometabolic risk factors. Cell Metab..

[356] Kitase Y., Vallejo J.A., Gutheil W., Vemula H., Jähn K., Yi J., Zhou J., Brotto M., Bonewald L.F. (2018). β-aminoisobutyric Acid, l-BAIBA, Is a Muscle-Derived Osteocyte Survival Factor. Cell Rep..

[357] Martin K.S., Azzolini M., Lira Ruas J. (2020). The kynurenine connection: How exercise shifts muscle tryptophan metabolism and affects energy homeostasis, the immune system, and the brain. Am. J. Physiol. Cell Physiol..

[358] Joisten N., Walzik D., Metcalfe A.J., Bloch W., Zimmer P. (2020). Physical Exercise as Kynurenine Pathway Modulator in Chronic Diseases: Implications for Immune and Energy Homeostasis. Int. J. Tryptophan Res..

[359] Zimmer P., Schmidt M.E., Prentzell M.T., Berdel B., Wiskemann J., Kellner K.H., Debus J., Ulrich C., Opitz C.A., Steindorf K. (2019). Resistance Exercise Reduces Kynurenine Pathway Metabolites in Breast Cancer Patients Undergoing Radiotherapy. Front. Oncol..

[360] Saran T., Turska M., Kocki T., Zawadka M., Zieliński G., Turski W.A., Gawda P. (2021). Effect of 4-week physical exercises on tryptophan, kynurenine and kynurenic acid content in human sweat. Sci. Rep..

[361] Stone T.W., Clanchy F.I.L., Huang Y.S., Chiang N.Y., Darlington L.G., Williams R.O. (2022). An integrated cytokine and kynurenine network as the basis of neuroimmune communication. Front. Neurosci..

[362] Stanford K.I., Lynes M.D., Takahashi H., Baer L.A., Arts P.J., May F.J., Lehnig A.C., Middelbeek R.J.W., Richard J.J., So K. (2018). 12,13-diHOME: An Exercise-Induced Lipokine that Increases Skeletal Muscle Fatty Acid Uptake. Cell Metab..

[363] Yin X., Chen Y., Ruze R., Xu R., Song J., Wang C., Xu Q. (2022). The evolving view of thermogenic fat and its implications in cancer and metabolic diseases. Signal Transduct. Target. Ther..

[364] Gurup A., Yakal S., Tarçın G., Şahinler Ayla S., Turan H., Toprak M.S., Gungor Z.B., Ercan O. (2023). Effect of acute exercise on 12,13-dihydroxy-9Z-octadecenoic acid (12,13-diHOME) levels in obese male adolescents. Clin. Endocrinol..

[365] Wang Z., Yu X., Chen Y. (2021). Recruitment of Thermogenic Fat: Trigger of Fat Burning. Front. Endocrinol..

[366] Hou Z., Qin X., Hu Y., Zhang X., Li G., Wu J., Li J., Sha J., Chen J., Xia J. (2019). Longterm exercise-derived exosomal miR-342-5p: A novel exerkine for cardioprotection. Circ. Res..

[367] Doncheva A.I., Romero S., Ramirez-Garrastacho M., Lee S., Kolnes K.J., Tangen D.S., Olsen T., Drevon C.A., Llorente A., Dalen K.T. (2022). Extracellular vesicles and microRNAs are altered in response to exercise, insulin sensitivity and overweight. Acta Physiol..

[368] Wang Y., Ding S. (2024). Extracellular vesicles in cancer cachexia: Deciphering pathogenic roles and exploring therapeutic horizons. J. Transl. Med..

[369] Hu W., Xiong H., Ru Z., Zhao Y., Zhou Y., Xie K., Xiao W., Xiong Z., Wang C., Yuan C. (2021). Extracellular vesicles-released parathyroid hormone-related protein from Lewis lung carcinoma induces lipolysis and adipose tissue browning in cancer cachexia. Cell Death Dis..

[370] Marzan A.L., Chitti S.V. (2023). Unravelling the Role of Cancer Cell-Derived Extracellular Vesicles in Muscle Atrophy, Lipolysis, and Cancer-Associated Cachexia. Cells.

[371] Di Felice V., Barone R., Trovato E., D’Amico D., Macaluso F., Campanella C., Marino Gammazza A., Muccilli V., Cunsolo V., Cancemi P. (2022). Physiactisome: A New Nanovesicle Drug Containing Heat Shock Protein 60 for Treating Muscle Wasting and Cachexia. Cells.

[372] Peng B., Yang Y., Wu Z., Tan R., Pham T.T., Yeo E.Y.M., Pirisinu M., Jayasinghe M.K., Pham T.C., Liang K. (2023). Red blood cell extracellular vesicles deliver therapeutic siRNAs to skeletal muscles for treatment of cancer cachexia. Mol. Ther..

[373] Roeland E.J., Bohlke K., Baracos V.E., Bruera E., Del Fabbro E., Dixon S., Fallon M., Herrstedt J., Lau H., Platek M. (2020). Management of Cancer Cachexia: ASCO Guideline. J. Clin. Oncol..

[374] Bland K.A., Krishnasamy M., Parr E.B., Mulder S., Martin P., van Loon L.J.C., Cormie P., Michael N., Zopf E.M. (2022). “I want to get myself as fit as I can and not die just yet”—Perceptions of exercise in people with advanced cancer and cachexia: A qualitative study. BMC Palliat. Care.

[375] Moreira V.M., da Silva Franco C.C., Prates K.V., Gomes R.M., de Moraes A.M.P., Ribeiro T.A., Martins I.P., Previate C., Pavanello A., Matiusso C.C.I. (2018). Aerobic Exercise Training Attenuates Tumor Growth and Reduces Insulin Secretion in Walker 256 Tumor-Bearing Rats. Front. Physiol..

[376] Niels T., Tomanek A., Freitag N., Schumann M. (2020). Can Exercise Counteract Cancer Cachexia? A Systematic Literature Review and Meta-Analysis. Integr. Cancer Ther..

[377] Orange S.T., Leslie J., Ross M., Mann D.A., Wackerhage H. (2023). The exercise IL-6 enigma in cancer. Trends Endocrinol. Metab..

